# Design, Synthesis, and Antiviral Evaluation of Sialic Acid Derivatives as Inhibitors of Newcastle Disease Virus Hemagglutinin-Neuraminidase: A Translational Study on Human Parainfluenza Viruses

**DOI:** 10.1021/acsinfecdis.2c00576

**Published:** 2023-02-27

**Authors:** Paola Rota, Paolo La Rocca, Francesco Bonfante, Matteo Pagliari, Marco Piccoli, Federica Cirillo, Andrea Ghiroldi, Valentina Franco, Carlo Pappone, Pietro Allevi, Luigi Anastasia

**Affiliations:** † Department of Biomedical, Surgical and Dental Sciences, Università degli Studi di Milano, 20133 Milan, Italy; ‡ Institute for Molecular and Translational Cardiology (IMTC), San Donato Milanese, 20097 Milan, Italy; § Department of Biomedical Sciences for Health, Università degli Studi di Milano, 20133 Milan, Italy; ∥ Division of Comparative Biomedical Sciences, Istituto Zooprofilattico Sperimentale delle Venezie, 35020 Legnaro, Italy; ⊥ Laboratory of Stem Cells for Tissue Engineering, IRCCS Policlinico San Donato, San Donato Milanese, 20097 Milan, Italy; # Division of Clinical and Experimental Pharmacology, Department of Internal Medicine and Therapeutics, University of Pavia, 27100 Pavia, Italy; ∇ IRCCS, Mondino Foundation, 27100 Pavia, Italy; ○ Arrhythmology Department, IRCCS Policlinico San Donato, Piazza Malan 2, San Donato Milanese, 20097 Milan, Italy; ◆ Faculty of Medicine, University of Vita-Salute San Raffaele, 20132 Milan, Italy

**Keywords:** Newcastle disease virus, sialic acid, antiviral inhibitor, human parainfluenza viruses, hemagglutinin-neuraminidase, viral infection

## Abstract

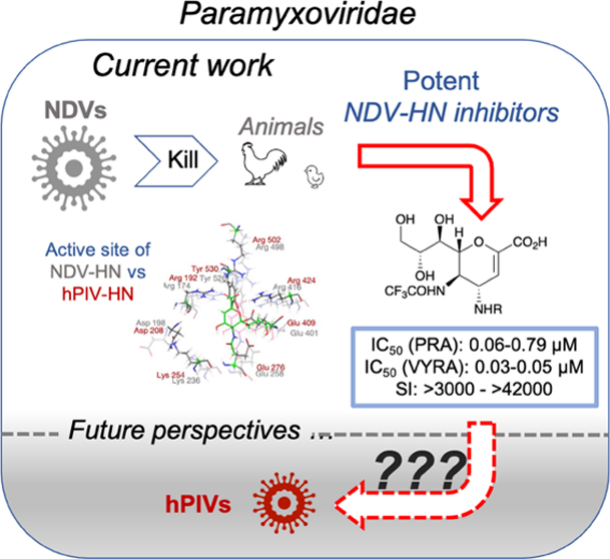

Global infections with viruses belonging to the *Paramyxoviridae*, such as Newcastle disease virus (NDV) or human parainfluenza viruses (hPIVs), pose a serious threat to animal and human health. NDV-HN and hPIVs-HN (HN hemagglutinin-neuraminidase) share a high degree of similarity in catalytic site structures; therefore, the development of an efficient experimental NDV host model (chicken) may be informative for evaluating the efficacy of hPIVs-HN inhibitors. As part of the broad research in pursuit of this goal and as an extension of our published work on antiviral drug development, we report here the biological results obtained with some newly synthesized C4- and C5-substituted 2,3-unsaturated sialic acid derivatives against NDV. All developed compounds showed high neuraminidase inhibitory activity (IC_50_ 0.03–13 μM). Four molecules (**9**, **10**, **23**, **24**) confirmed their high *in vitro* inhibitory activity, which caused a significant reduction of NDV infection in *Vero cells*, accompanied by very low toxicity.

The development of new therapies against infections caused by *Orthomyxoviridae*, such as influenza viruses, and *Paramyxoviridae*, such as Newcastle disease virus (NDV) and human parainfluenza viruses (hPIVs), is of critical importance to veterinary and human medicine.^1−3^ In recent years, the 2,3-unsaturated sialic acid compound **1** (DANA or Neu5Ac2en, Figure 1) has emerged as an effective scaffold for the construction of potent and selective viral neuraminidase (N)^4−7^ and hemagglutinin-neuraminidase (HN)^8−16^ inhibitors (**2**–**8**), as demonstrated by the discovery of commercially available influenza drugs such as Zanamivir **2**, Laninamivir **3**, and their cyclopentane (Peramivir **6**) and cyclohexyl derivatives (Oseltamivir **7**).^2,4−7^ Thus, while drugs are available against influenza A and B viruses, there are currently no approved antiviral agents against NDV and hPIVs, despite a large number of molecules that have been developed.^8−11,14−16^

**Figure 1:**
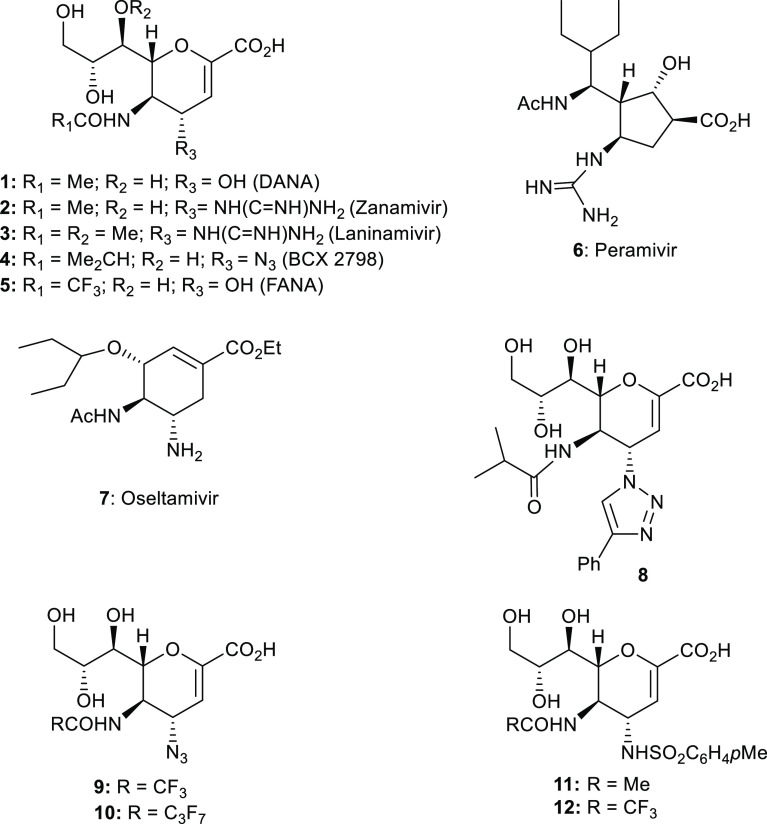
Reported inhibitors against *Orthomyxoviridae* (as influenza viruses) and *Paramyxoviridae* (hPIVs and NDV). DANA **1** and FANA **5** are pan-selective inhibitors.

The HN is an ideal drug target due to its diverse regulatory functions at various stages of the *Paramyxoviridae* life cycle^8−17^ The availability of the crystal structure of NDV-HN^18^ and later hPIV3-HN,^19^ as well as advances in molecular modeling, led to the development of potent hPIV-HN inhibitors.^8−11,14−16^ Currently, BCX 2798 **4**,^14,15^ developed on the scaffold NDV-HN is the most potent inhibitor against hPIV1-HN *in vitro* to date. Otherwise, the C4-modified phenyltriazole derivative of BCX 2798, compound **8**,^9^ showed the lowest IC_50_ values for hPIV3-HN. Despite the good *in vitro* results obtained with these hPIVs-HN inhibitors, the treatment of choice to date is the combined use of general drugs such as ribavirin with corticosteroids and/or epinephrine.

It is therefore important that new drug candidates are continually developed in this field, and their biological and virological efficacy must be thoroughly evaluated using appropriate experimental models. In this context, we have recently developed very potent *Paramyxoviridae* neuraminidase activity inhibitors directed against Newcastle disease virus, namely, azido compounds **9** and **10** and the *p*-toluensulfonamido derivatives **11** and **12** (Figure 1).^13^ NDV is the causative agent of one of the most devastating poultry diseases, which represents an enormous burden to the world economy.^20^ The catalytic site of NDV-HN exhibits a high degree of sequence similarity^19^ to that of hPIVs-HN, and was initially used as a template for the development of some inhibitors, such as BCX 2798.^14^ In addition, the well-conserved active site structure of these enzymes^19^ (Figure 2) suggests the possibility of using NDV-HN not only for virtual screening for drug discovery but also for building an experimental model to predict the inhibitory effect of the synthesized compounds on hPIVs-HN.

**Figure 2:**
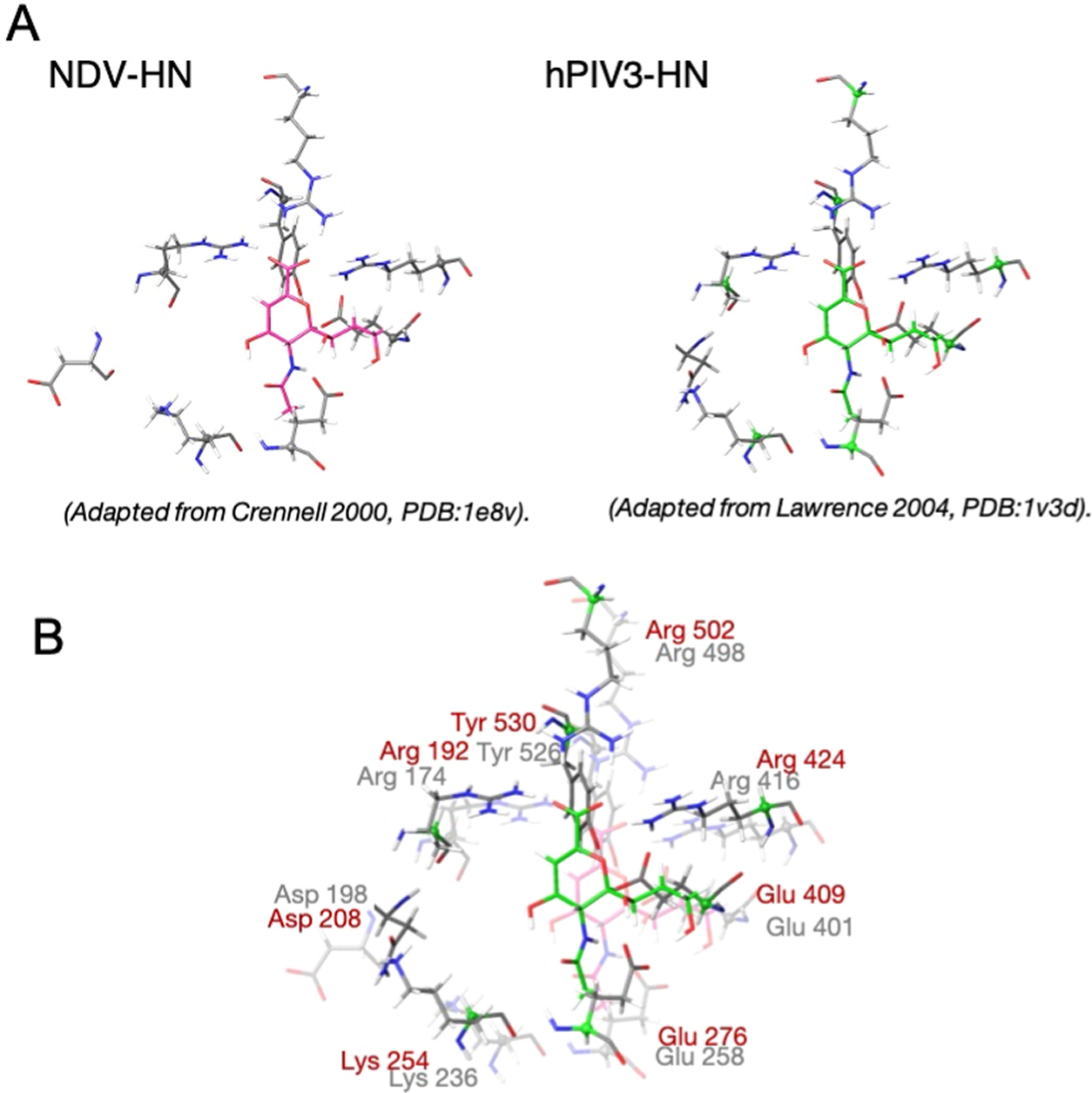
(A) Representation of amino acid residues around the NDV-HN and hPIV3-HN binding sites (based on NDV-HN or hPIV3-HN co-crystal structure in complex with **DANA 1**, adapted from PDB ID: 1e8v and 1v3d, respectively).^18,19^ (B) Overlap of NDV (purple) and hPIV3 (green) catalytic sites shows highly conserved catalytic amino acids.

As part of a larger body of work^21−23^ on the development of inhibitors against the *Paramyxoviridae* family and as a natural extension of our earlier studies,^12,13,21−27^ we report here the synthesis and biological evaluation of several new inhibitors against NDV-HN. Remarkably, the synthesis of some new potent C4- and C5-modified NDV-HN inhibitors succeeded starting from the structure of compound **11** previously selected by docking studies.^13^ Moreover, the most active synthesized compounds were first tested on three different strains of NDV-HN to confirm their efficacy, and then an in-depth biological and virological evaluation was performed. In particular, by setting up some specific assays, we have shown that the synthesized molecules act mainly by inhibiting the neuraminidase activity of NDV-HN. Furthermore, we reported for the first time the inhibitory effect of Zanamivir **2** and BCX 2798 **4** on NDV-HN and used them for comparison with our inhibitors.

This study provides promising results that will allow us to test these inhibitors on hPIVs and gain new insights into the potential of using NDV-HN as a predictive and translational model.

## Results and Discussion

### Structural Optimization of Compound 11: Synthesis and Biological Neuraminidase Activity Evaluation of New C4- and C5-Substituted Derivatives

**Figure 3:**
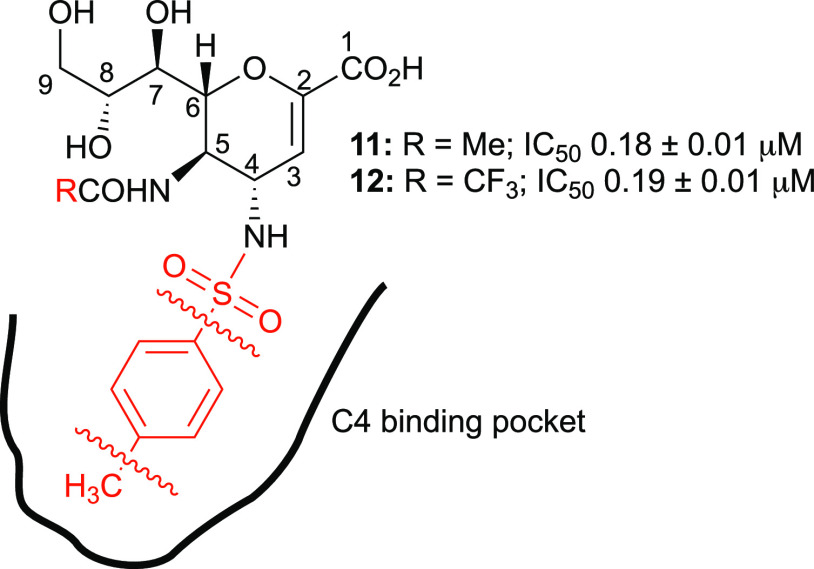
Representation of inhibitors **11** and **12** having a C4 *p*-toluenesulfonamido substituent placed in the adjacent binding pocket. The three portions (methyl-phenyl-sulfonamido) of the large C4 sulfonamido substituent are depicted in red, and the carbon atom numbering for the DANA scaffold is reported.

In the previous study,^13^ we found that the C4 sulfonamido derivative **11**, selected by docking screening, and its C5 trifluoroacetamido analogue **12** exhibited an impressive neuraminidase inhibitory activity against the NDV-HN (**11**, IC_50_ = 0.18 μM; **12**, IC_50_ = 0.19 μM), showing that the C4 binding pocket can accommodate large groups (Figure 3). In addition, azido derivative **9** with a smaller substituent at the C4 position also showed high inhibitory activity (IC_50_ 0.17 μM). This observation prompted us to investigate the individual influence of the different smaller portions of the large C4 *p*-toluenesulfonamido substituent on the IC_50_ value.

**Scheme 1:**
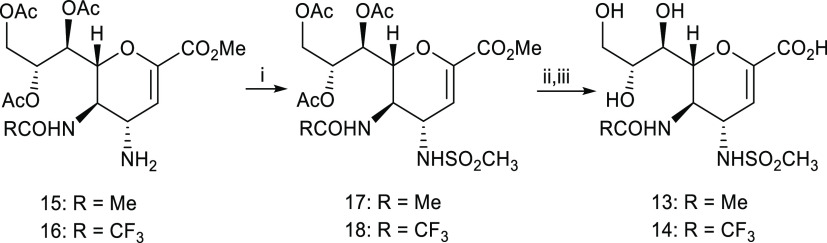
Synthesis of Inhibitors **13** and **14** i) Methanesulfonyl chloride, Et_3_N, CH_2_Cl_2_, 0–23 °C, 1–2 h, 63–65%; (ii) NaOMe, MeOH, 23 °C, 1 h; (iii) Et_3_N, MeOH/H_2_O (2:1, v/v), 23 °C, overnight, overall two-step yields 66–69%.

As we demonstrated previously,^12,13^ replacing the C5 acetamido group with the trifluoroacetylated one improved the inhibitory activity against NDV-HN, so we first decided to test this effect on molecules with a small C4 substituent. Therefore, we planned to synthesize compounds **13** and **14**, with methanesulfonamido groups at C4 and an acetyl or trifluoroacetylamido functions at C5. The synthesis of compounds **13**(28) and **14** began with the previously reported protected C4 amino DANA derivatives **15**(8) and **16**(13) (Scheme 1). Sulfonylamides **17** and **18** were prepared with methanesulfonyl chloride in the presence of Et_3_N. Subsequent deprotection of both the *O*-acetyl groups with standard Zemplén conditions and the C1-methyl ester by selective hydrolysis with Et_3_N in aqueous methanol afforded the final compounds **13**(28) and **14**, in good yields (66–69%).

Then, both derivatives **13** and **14** were tested for their inhibitory activity against NDV-HN by performing a neuraminidase inhibition (NI) assay using the fluorogenic neuraminidase substrate 2′-(4-methylumbelliferyl)-α-d-*N*-acetylneuraminic acid (4-MUNeu5Ac) and using *in-toto* purified NDV La Sota (Clone 30). The IC_50_ values of compounds **13** and **14** (13 and 6.3 μM, respectively) confirmed that the substitution of the acetamido group at C5 with the trifluoroacetamido group provided a statistically significant improvement in inhibitory activity. However, the IC_50s_ of compounds **13** and **14** were higher than those of *p*-toluensulfonamido derivatives **11** and **12**, demonstrating the important role of the aromatic moiety in ligand-active site interactions.

In view of these results, to complete our study, we planned to synthesize a pool of new C4-substituted molecules, leaving the C5 trifluoroacetylamido substituent unchanged. We examined the effect of C4 substituents containing phenyl and trifluoromethylsulfonamido groups or an acylamide function in place of the sulfonamide group on the inhibitory activity of neuraminidase to evaluate the influence of replacing the sulfonyl group with the carbonyl group. To this end, we planned to synthesize the new compounds C4-benzylsulfonamido **19** and C4-trifluoromethylsulfonamido **20** to complete the series of sulfonamides and that of their corresponding acylamide analogues **21**–**24**, which have not been described previously.

**Scheme 2:**
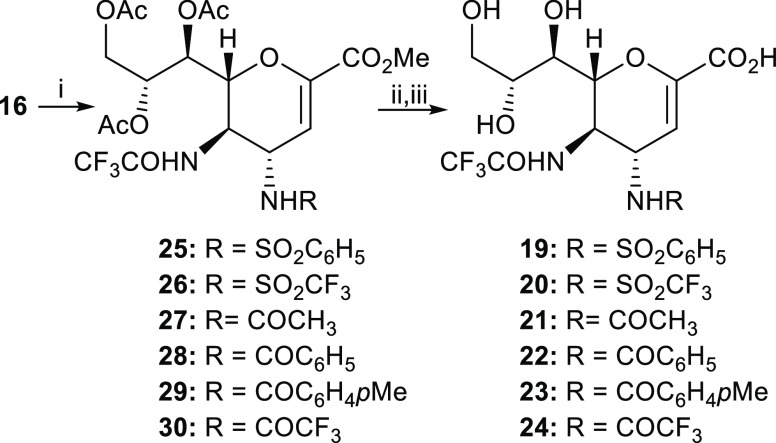
Synthesis of Inhibitors **19**–**24** (i) Appropriate sulfonylating or acylating agent, Et_3_N, CH_2_Cl_2_, 0–23 °C (−78 °C for 26), 1–3 h, 60–80%; (ii) NaOMe, MeOH, 23 °C, 1 h; (iii) Et_3_N, MeOH/H_2_O (2:1, v/v), 23 °C, overnight, overall two-step yields 55–66%.

The synthetic pathway (Scheme 2) for all compounds began with the common precursor **16** and was similar to that reported for the synthesis of compound **14**. Sulfonylation with benzenesulfonyl chloride or trifluoromethanesulfonic anhydride afforded the protected derivatives **25** and **26**, respectively, in good yields (62–70%). Due to the high reactivity of trifluoromethanesulfonic anhydride, we modified the classical acylation conditions for the synthesis of compound **26** and carried out the reaction at −78 °C instead of 23 °C.

Acylamido derivatives **27**–**30** were obtained in high yields (71–80%) using the appropriate acylating agent: acetyl chloride, benzoyl chloride, *p*-toluoyl chloride, and trifluoroacetic anhydride, respectively. Finally, the free derivatives **19**–**24** were obtained by the two-step deprotection described above.

**Figure 4:**
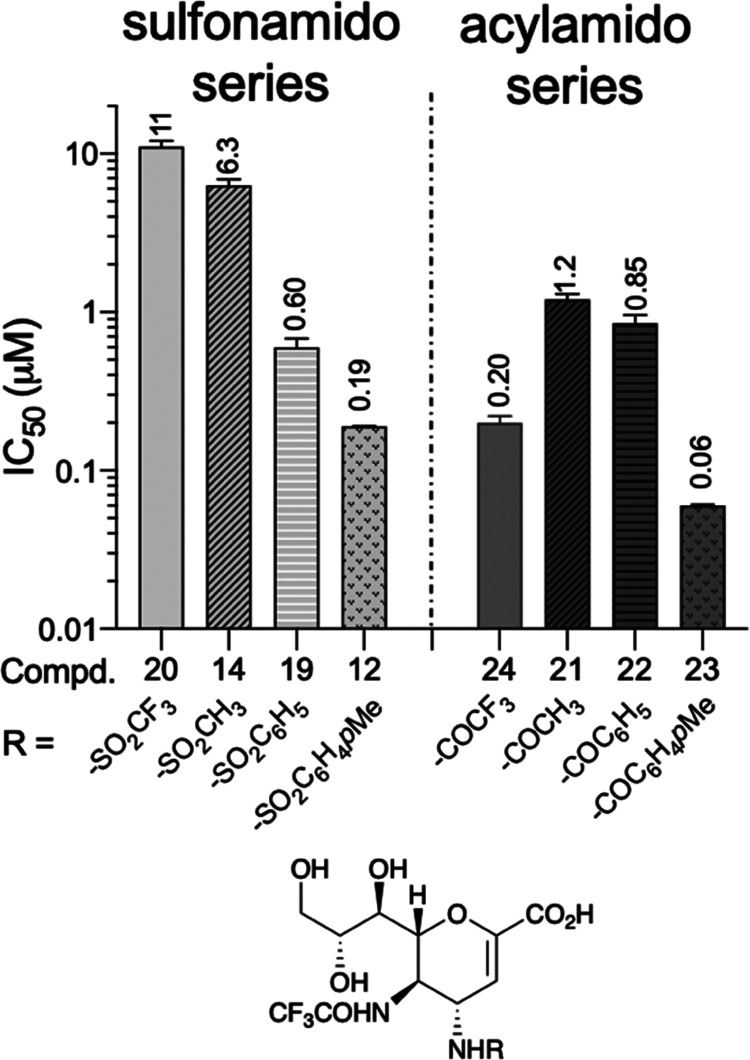
IC_50_ values for FANA **5** analogues modified at C4 position obtained on La Sota Clone 30 NDV-HN. Each value represents the mean of three independent experiments carried out in triplicate (see also Table S1 in the Supporting Information for the standard deviations, SD values).

All final free acidic compounds **19**–**24** were evaluated for their inhibitory activity against NDV-HN by performing the neuraminidase inhibition assay (NI) with in-toto purified NDV La Sota (clone 30). Their IC_50_ values are shown together with those of compounds **12** and **14** in Figure 4 (see also Table S1 in the Supporting Information).

The positive contribution that fluorine atoms generally make to ligand-active site interactions prompted us to investigate the substitution of the methylsulfonamido group present in compound **14** by the trifluoromethylsulfonamido group of inhibitor **20**. Unexpectedly, compound **20** showed as little activity as methylsulfonamido **14** (IC_50_ 11 and 6.3 μM, respectively). Interestingly, substitution of the sulfonyl group of **20** with the carbonyl group to form the trifluoroacetamido **24** resulted in a significant increase in activity with an IC_50_ value in the nanomolar range (0.20 μM). However, this significant improvement in activity was lost with the nonfluorinated C4 acetamido analogue **21** (IC_50_ 1.2 μM). Taken together, these results confirm that the presence of both the carbonyl group and the fluorine atoms at C4 is essential for enhancing the inhibitory effect. Thus, the trifluorocetyl group represents a good alternative among low-hindered substituents to the azido group.

In the sulfonamide series, we observed a 10-fold increase in potency from the methylsulfonamido derivative **14** (IC_50_ 6.3 μM) to the benzenesulfonamido analogue **19** (0.60 μM), supporting the positive contribution of the aromatic ring to the inhibitor-active site interactions. A further increase in potency was observed upon transition to *p*-toluenesulfonamide **12** (IC_50_ 0.19 μM). These data underscore the essential role of the *p*-methyl group in accommodating the hindering substituent in the large hydrophobic C4 binding cavity, as correctly predicted in the previously published docking simulation study.^13^

It is also noteworthy that replacement of the *p*-toluenesulfonamido group in **12** with a *p*-toluoylamido group in **23** resulted in a 3-fold increase in inhibitory activity, confirming that a carbonyl group is probably better able to accommodate the large substituent in the C4 pocket than the sulfonyl group.

**Scheme 3:**
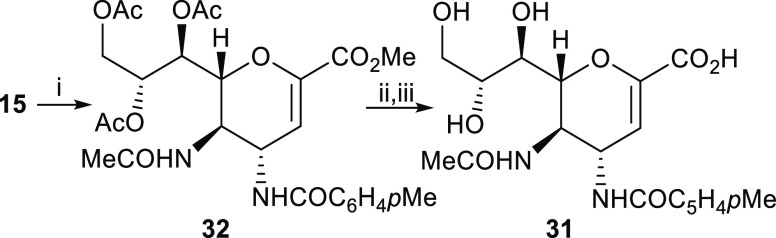
Synthesis of Inhibitor **31** (i) *p*-Toluoyl chloride, Et_3_N, CH_2_Cl_2_, 0–23 °C, 2–3 h, 79%; (ii) NaOMe, MeOH, 23 °C, 1 h, (iii) Et_3_N, MeOH/H_2_O (2:1, v/v), 23 °C, overnight, overall two-step yield 52%.

Derivative **23** was clearly the most potent inhibitor of NDV-HN ever reported and it was 40 times more active than FANA **5**.^12^ To complete this structural optimization study, we decided to confirm that the presence of the trifluoroacetamido group at C5 in compound **23** was an essential element for maintaining the observed high potency. To this end, we synthesized and tested the corresponding C5 acetamido derivative **31**. As shown in Scheme 3, acylation of compound **15**(8) with *p*-toluoyl chloride afforded intermediate **32**. Subsequent two-step deprotection of this intermediate provided the deprotected free derivative **31**. The NI assay performed with this compound gave an IC_50_ value of 1.1 μM, confirming the assumption that the trifluoroacetamido group at C5 is essential for enhancing the inhibitory activity.

### Neuraminidase Inhibitory Activity Evaluation against Selected Lentogenic and Velogenic NDV Strains

**Figure 5:**
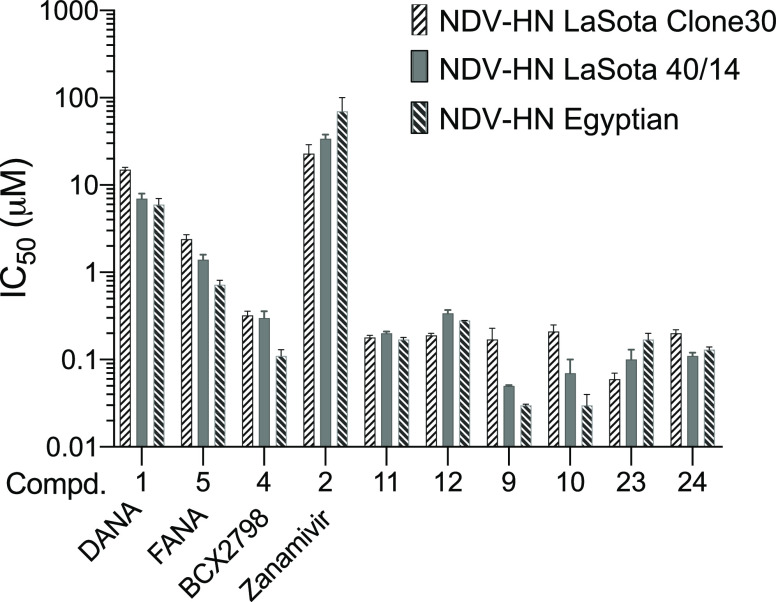
IC_50_ values of neuraminidase inhibitors against three NDV strains (two NDV La Sota and the velogenic strain APMV-1/chicken/Egypt/13VIR-5009-2/2013). Each value represents the mean of three independent experiments carried out in triplicate.

To rule out strain dependence of the observed biological inhibitory values, derivatives **23** and **24**, together with four previously published potent inhibitors **9**–**12**,^13^ were selected for further evaluation of inhibitory activity against two additional NDV strains (a lentogenic vaccine strain La Sota and a velogenic strain APMV-1/chicken/Egypt/13VIR-5009-2/2013), using DANA **1** and FANA **5** as reference standards. A gap in the literature was also addressed by synthesizing and testing the known influenza agent Zanamivir **2** and the most potent inhibitor of hPIV1-HN, BCX 2798 **4**,^1^ against NDV-HN. In fact, while the IC_50_ values of Zanamivir **2** and BCX 2798 **4** are known for hPIVs, the inhibitory activity for NDV has not yet been reported. The results showed that the inhibitory activity values of all tested compounds were in a similar range for the three tested strains, although some statistically significant variations were observed (see Figure 5). These results are compatible with the alignments of the head domain sequences, which show no variation in the amino acids surrounding the active site among the three different strains (see Figure S1 in the Supporting Information). In particular, DANA **1** and FANA **5** confirmed their IC_50_ values in the micromolar range (6.2–14 μM), while Zanamivir was shown to have worse inhibitory activity (IC_50_ 23–70 μM).^1^ According to these results, this commercial drug is not very effective against paramyxoviruses. Otherwise, BCX 2798 showed low IC_50_ values against all three different strains of NDV-HN (IC_50_ between 0.11 and 0.32 μM), confirming a comparable inhibitory effect to hPIV1-HN (literature IC_50_ 0.04,^14^ 0.32^16^ and 0.50^8^ μM) but higher than hPIV3-HN (literature IC_50_ 20^14^ and 21.5^9^ μM). Based on these results, computational studies at NDV-HN are well suited to develop inhibitors against hPIV1-HN for which no crystal structure is available.

Interestingly, it was also observed that our previously synthesized,^13^ C4 azido derivatives **9** and **10**, which differ from BCX 2798 only for the C5 moiety, were 2- to 6-fold more active against all three strains (IC_50_ values around 0.03 μM). In summary, the C4 azido group appears to exert a considerable effect (particularly in velogenic strains like BCX 2798), so we selected derivatives **9** and **10** to be tested biologically. Based on their high *in vitro* inhibitory activity (IC_50_ ≤ 0.20 μM), which was maintained against all three strains, we also selected the newly synthesized C4 *p*-tolyl analogue **23** (more active than *p*-toluenesulfonyl derivatives **11** and **12**) and the unprocessed C4 trifluoroacetamido analogue **24**.

### Activity of the Compounds against Newcastle Disease Virus Replication

**Figure 6:**
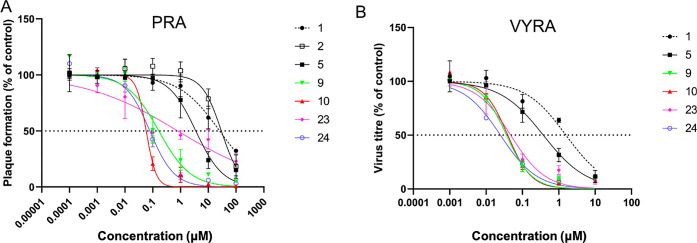
Antiviral activity of compounds **1**, **2**, **5**, **9**, **10**, **23**, and **24** in *Vero cells* infected with NDV La Sota. (A) Inhibition curve of the tested compounds by performing the plaque reduction assay (PRA); (B) inhibition curve of the tested compounds by performing the virus yield reduction assay (VYRA). Data is graphed as the mean, and error bars represent the standard error. IC_50_ values were calculated using Prism software and are representative of three independent experiments.

We examined the antiviral effects of selected compounds in *Vero cells* infected with NDV. First, we tested the compounds in plaque reduction assays (PRA) using the NDV La Sota strain (see Table 1 and Figure 6). Reference compounds **1** and **5** had IC_50_ values in the micromolar range, consistent with previous data obtained *in vitro*.

**Table 1:** Antiviral Activities against NDV-HN and Cytotoxicity of Selected Compounds **1**, **2**, **5**, **9**, **10**, **23**, and **24** in *Vero Cells*

cmpd	plaque reduction assay (IC_50_, μM)	viral yield reduction assay 24 h (IC_50_, μM)	cytotoxicity assay (CC_50_, μM)	selectivity index^a^ (CC_50_/IC_50_)
1	26 ± 13	1.6 ± 0.6	>2500	>96
2	30 ± 7		>2500	>83
5	3.5 ± 0.9	0.37 ± 0.08	>2500	>714
9	0.17 ± 0.06	0.04 ± 0.01	>2500	>14,705
10	0.06 ± 0.02	0.04 ± 0.01	>2500	>41,667
23	0.79 ± 0.57	0.05 ± 0.02	>2500	>3164
24	0.08 ± 0.01	0.03 ± 0.01	>2500	>32,051

a Selectivity index (SI): ratio between CC_50_ and IC_50_ observed in PRA.

Compounds **9**, **10**, **23**, and **24** inhibited plaque formation with IC_50_ values in the submicromolar range; compounds **10** and **24** in particular proved to be the most potent molecules. No cytotoxic effects were observed with any of the compounds, even at very high concentrations (concentrations up to 2.5 mM were tested). Based on these results, we calculated the selectivity index (SI, see Table 1) for all compounds as the ratio between the cytotoxic concentration 50 (CC_50_) and IC_50_. This value is important to evaluate the efficacy of antivirals. The high values observed for compounds **9**, **10**, **23**, and **24** indicate that these molecules are not only very effective against viral growth but also theoretically very safe when used for in vivo experiments.

To further characterize the anti-neuraminidase activity of the compounds, we examined the effects of the different concentrations on the production of infectious progeny using the virus yield reduction assay (VYRA, see Table 1 and Figure 6). At 24 h post-infection (p.i.), the supernatants of infected cells were harvested and titrated using the microplaque assay. A dose-dependent reduction in viral progeny was observed for all compounds tested.

Notably, compounds **9**, **10**, **23**, and **24** exhibited IC_50_ values in the low nanomolar range, confirming a 10- to 100-fold reduction in IC_50_ compared with compounds **1** and **5**, which served as references. For all compounds, IC_50_ values in VYRA were lower than those obtained in PRA, probably due to the different conditions of the assays in terms of multiplicity of infection (MOI) and time of virus growth. We noticed that compound **23** recorded an unexpected low inhibitory activity in the PRA assay compared to the structurally similar compounds **9**, **10**, and **24**, especially considering the comparable molarity range observed for these compounds in the neuraminidase inhibition assay and the VYRA assay. We speculate that this compound might suffer from suboptimal availability/solubility when suspended in the Avicel overlay medium used in the PRA assay, as opposed to the liquid culture medium used for the VYRA assay. Avicel is a colloidal form of water-insoluble cellulose microparticles with relatively low viscosity. Nonetheless, we did not explore the solubility profile of all compounds in this medium; hence, our observation is merely speculative in its nature and will require further investigation.

### Inhibition Mechanisms Evaluation: Attachment or Release?

To better understand the mechanism responsible for the inhibitory effect of the synthesized compounds and their influence on the activity of the HN protein during infection, we planned and optimized two different assays to distinguish whether our molecules could affect the entry or release of virus particles. To test the effect on virus entry, the antiviral compounds were added before the virus docked onto the cells. Conversely, to test the effect on the release of the virus, the molecules were administered to the cells after the virus had entered.

**Figure 7:**
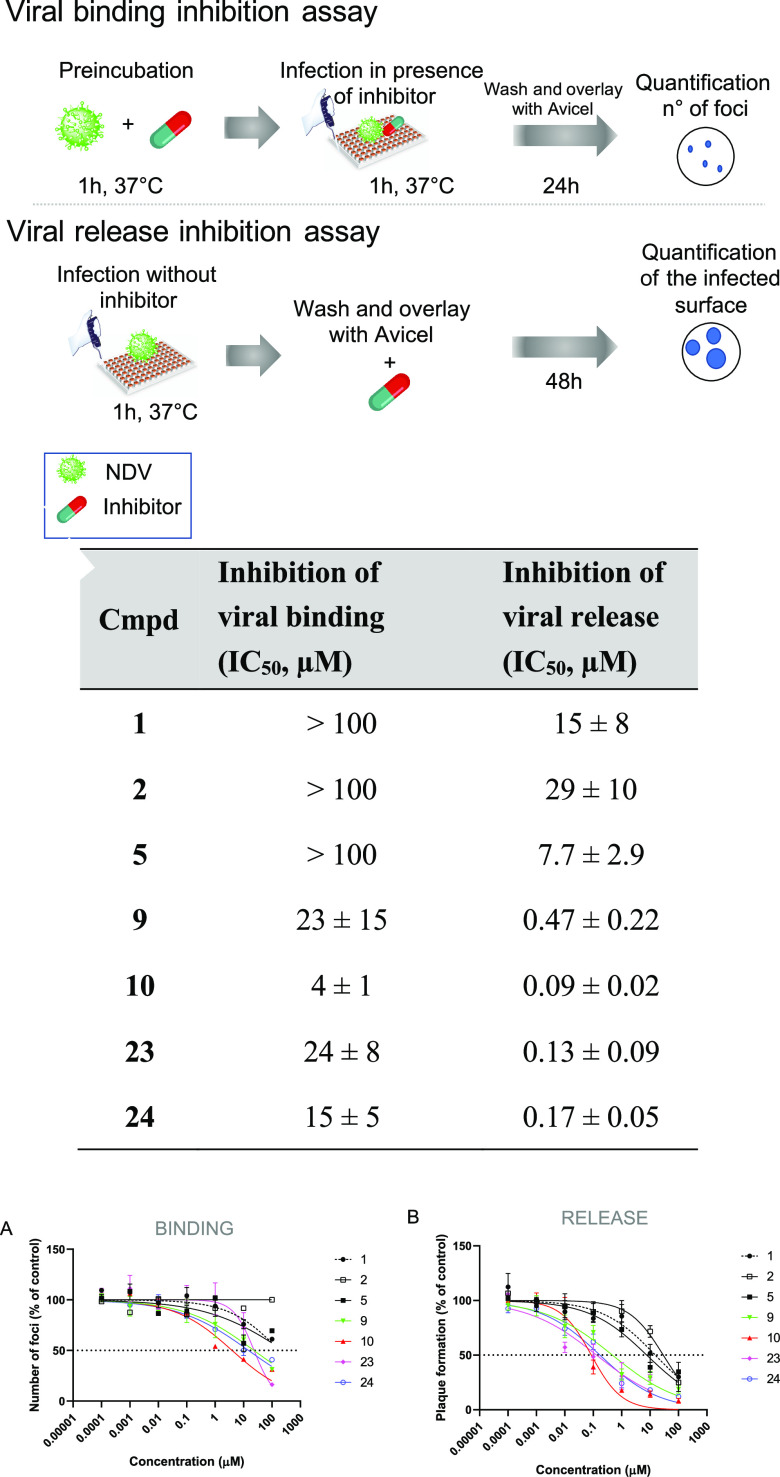
Inhibition of viral binding and viral release by compounds in *Vero cells* infected with NDV La Sota. (A) Viral binding inhibition assay performed with compounds **1**, **2**, **5**, **9**, **10**, **23**, and **24** in *Vero cells* infected with NDV La Sota (see the Methods section). Data are graphed as the mean, and error bars represent the standard deviation. IC_50_ values were calculated using Prism software and are representative of three independent experiments. (B) Viral release inhibition assay performed with compounds **1**, **2**, **5**, **9**, **10**, **23**, and **24** in *Vero cells* infected with NDV La Sota (see the Methods section). Data are graphed as the mean, and error bars represent the standard error. IC_50_ values were calculated using Prism software and are representative of three independent experiments.

Treatment of *Vero cells* with the selected compounds (**9**, **10**, **23**, **24**) prior to virus docking showed that the newly synthesized molecules inhibited virus binding more efficiently than the antiviral drugs used as reference controls (**1**, **2**, **5**). While the IC_50_s of **1**, **2**, and **5** were well above 100 μM, our molecules **9**, **10**, **23**, and **24** showed lower IC_50_ values corresponding to 23, 4, 24, and 15 μM, respectively. By contrast, when antiviral drugs were administered after virus entry, their inhibitory effect on virus release was significantly stronger than their binding inhibition, as demonstrated by lower IC_50_ concentrations. Nevertheless, even in this case, the new compounds showed increased efficacy over the reference antiviral drugs, with IC_50_ values in the nanomolar range (**9**: 0.47 μM; **10**: 0.09 μM; **23**: 0.13 μM and **24**: 0.17 μM), as shown in Figure 7. Remarkably, the IC_50_ values measured by this assay were very similar to those measured by the plaque reduction assay, confirming the inhibitory activity of all compounds on neuraminidase activity, as it is responsible for the release of viral particles from infected cells.

Based on these results, it appears that the new compounds exert their antiviral properties by inhibiting neuraminidase activity rather than hemagglutinin activity.

## Conclusions

This study describes the design and synthesis of several high-activity (IC_50_ 0.03–13 μM) neuraminidase inhibitors, with no apparent cell toxicity. Four selected compounds (**9**, **10**, **23**, **24**) were able to significantly inhibit NDV infection of *Vero cells* by preventing the release of virus particles from infected cells. As for Zanamivir, the commercial drug was found to be ineffective against paramyxoviruses. These preliminary results lay the foundations for future studies of these molecules on hPIV1.

## Methods

### General Chemistry

All chemicals and solvents used were of analytical grade and purchased from Sigma-Aldrich (St. Louis, MO). Deionized water was prepared by filtering water on a Milli-Q Simplicity 185 filtration system from Millipore (Bedford, MA). Solvents were dried using standard methods and distilled before use. The progress of all reactions was monitored by thin-layer chromatography (TLC) carried out on 0.25 mm Sigma-Aldrich silica gel plates (60 F254) using UV light, anisaldehyde/H_2_SO_4_/EtOH solution or 0.2% ninhydrin in ethanol and heat as the developing agent. Flash chromatography was performed with normal-phase silica gel (Sigma-Aldrich 230–400 mesh silica gel). Nuclear magnetic resonance spectra were recorded at 298 K on a Bruker AM-500 spectrometer equipped with a 5 mm inverse-geometry broadband probe and operating at 500.13 MHz for ^1^H and 125.76 MHz for ^13^C. Chemical shifts are reported in parts per million and are referenced for ^1^H spectra to a solvent residue proton signal (δ = 7.26, 3.31, and 2.50 ppm, respectively, for CDCl_3_, CD_3_OD, and DMSO-*d*_6_) and for ^13^C spectra, to solvent carbon signal (central line at δ = 77.00, 49.05 and 39.43 ppm, respectively, for CDCl_3_, CD_3_OD and DMSO-d_6_). The chemical shifts for the spectra collected in CD_3_OD-D_2_O (1:1, v/v or 3:1, v/v) are referenced to the internal CH_3_OH residue proton signal (δ = 3.31 ppm for ^1^H spectra and δ = 49.05 ppm for ^13^C spectra). The ^1^H and ^13^C resonances were assigned by ^1^H–^1^H (COSY) and ^1^H-^13^C (HSQC and HMBC) correlation two-dimensional (2D) experiments. The ^1^H NMR data are tabulated in the following order: multiplicity (s = singlet, d = doublet, t = triplet, br = broad, m = multiplet, app = apparent), coupling constant(s) are given in hertz, number of protons, and assignment of proton(s). Optical rotations were taken on a PerkinElmer 241 polarimeter equipped with a 1 dm tube and the [α]_D_ values are given in 10^–1^ deg cm^2^/g and the concentrations are given in g per 100 mL. Mass spectrometry spectra were obtained on an ABSciex 4000Qtrap mass spectrometer equipped with an electrospray ionization (ESI) ion source. The spectra were collected in a continuous flow mode by connecting the infusion pump directly to the ESI source. Solutions of the compounds were infused at a flow rate of 0.01 mL/min, the spray voltage was set at 4.5 kV in the negative ion mode with a capillary temperature of 550 °C. Full-scan mass spectra were recorded by scanning an *m*/*z* range of 100–2000. The preparative high-performance liquid chromatography purifications were performed on a Dionex Ultimate 3000 instrument equipped with a Dionex RS variable wavelength detector, using an Atlantis C-18-Preper T3 ODB (5 μm, 19 mm × 10 mm) column and starting from 100% aqueous 0.1% (v/v) formic acid to 100% CH_3_CN as the eluent. The crude product was dissolved in water and the solution was filtered (polypropylene, 0.45 μm, 13 mm ø, PK/100) and injected into the HPLC, affording purified products. Purity was evaluated by the analytical HPLC system Thermo Scientific Dionex UltiMate 3000 coupled to UV detector VWD-3100 (Thermo Fisher Scientific, San Jose, CA). LC elution was performed using a Hypersil GOLD aQ 3 μM, 150 mm × 3 mm HPLC C8 column (Thermo Fisher Scientific, San Jose, CA). Chromatography was carried out at 25 °C using as mobile phase A water + 0.1% formic acid and as mobile phase B acetonitrile + 0.1% formic at a constant flow rate of 0.250 mL/min All synthesized compounds showed a purity > 95%.

The protected precursors **15** and **16** have been synthesized as previously described and all of their chemical–physical properties were superimposable with those reported in the literature.^8,13^

#### General Procedure for the Synthesis of **17**, **18**, **25**–**30**, and **32**

To a solution of the selected C4 amino glycal **15**(8) or **16**(13) (0.50 mmol) in dichloromethane (DCM) (6.7 mL) containing Et_3_N (246 μL, 2.50 mmol), cooled at 0 °C, the opportune acylating agent (0.75 mmol) was added. The mixture was stirred at 23 °C until the disappearance of the starting material (1–3 h), then quenched with NH_4_Cl and extracted with AcOEt. The organic layer was washed with NaHCO_3_ and, then, with brine and dried over anhydrous Na_2_SO_4_. The organic solvent was concentrated under reduced pressure and then purified by silica gel chromatography, using an appropriate eluent solvent system, to achieve the desired compound.

#### Preparation of Methyl-5-acetamido-7,8,9-tri-*O*-acetyl-2,6-anhydro-4-methylsulfonamido-3,4,5-trideoxy-d-glycero-d-galacto-non-2-enonate (**17**)

Starting from protected glycal **15**(8) (215 mg, 0.50 mmol), according to the general procedure and using methanesulfonyl chloride (58 μL, 0.75 mmol), compound **17** was obtained (160 mg, 63%), after flash chromatographic purification (eluting with AcOEt), as a white amorphous solid. Compound **17** showed: [α]_D_^23^ = +57.7 (*c* = 1.0 in chloroform); ^1^H NMR (500 MHz, CDCl_3_): δ = 6.62 (d, *J*_NH,5_ = 9.6 Hz, 1H; NHCOCH_3_), 6.10 (d, *J*_NH,4_ = 9.0 Hz, 1H; NHSO_2_CH_3_), 5.94 (d, *J*_3,4_ = 2.2 Hz, 1H; H-3), 5.46 (dd, *J*_7,6_ = 1.8, *J*_7,8_ = 4.3 Hz, 1H; H-7), 5.26 (ddd, *J*_8,9a_ = 2.4, *J*_8,7_ = 4.3, *J*_8,9b_ = 7.8 Hz, 1H; H-8), 4.67 (dd, *J*_9a,8_ = 2.4, *J*_9a,9b_ = 12.4 Hz, 1H; H-9a), 4.37 (dd, *J*_6,7_ = 1.8, *J*_6,5_ = 10.3 Hz, 1H; H-6), 4.28 (ddd, *J*_4,3_ = 2.2, *J*_4,5_ = *J*_4,NH_ = 9.0 Hz, 1H; H-4), 4.18–4.09 (overlapping, 2H; H-9b and H-5), 3.77 (s, 3H; COOCH_3_), 2.98 (s, 3H; NHSO_2_CH_3_), 2.06–2.01 (overlapping, 9H; 3 × OCOCH_3_), 1.95 ppm (s, 3H; NHCOCH_3_); ^13^C NMR (125 MHz, CDCl_3_): δ = 172.0, 170.6, 170.5, 169.9 (4C; NHCOCH_3_ and 3 × OCOCH_3_), 161.7 (C-1), 144.4 (C-2), 111.2 (C-3), 77.2 (C-6), 71.4 (C-8), 68.1 (C-7), 62.1 (C-9), 52.4 (COOCH_3_ and C-4), 47.4 (C-5), 42.3 (NHSO_2_CH_3_), 23.1 (NHCOCH_3_), 20.8 (OCOCH_3_), 20.7 (OCOCH_3_), 20.6 ppm (OCOCH_3_); MS (ESI negative): *m*/*z* 507.1 [M – H]^−^; elemental analysis calcd (%) for C_19_H_28_N_2_O_12_S: C 44.88, H 5.55, N 5.51; found: C 44.71, H 5.35, N 5.55.

#### Preparation of Methyl-7,8,9-tri-*O*-acetyl-2,6-anhydro-4-methylsulfonamido-3,4,5-trideoxy-5-(2,2,2-trifluoroacetamido)-d-glycero-d-galacto-non-2-enonate (**18**)

Starting from protected glycal **16**(13) (242 mg, 0.50 mmol), according to the general procedure and using methanesulfonyl chloride (58 μL, 0.75 mmol), compound **18** was obtained (183 mg, 65%), after flash chromatographic purification (eluting with AcOEt/hexane, 6:4 v/v), as a white amorphous solid. Compound **18** showed: [α]_D_^23^ = +75.4 (*c* = 1.0 in chloroform); ^1^H NMR (500 MHz, CDCl_3_): δ = 7.78 (d, *J*_NH,5_ = 9.6 Hz, 1H; NHCOCF_3_), 5.94 (d, *J*_3,4_ = 2.3 Hz, 1H; H-3), 5.48 (d, *J*_NH,4_ = 9.7 Hz, 1H; NHSO_2_CH_3_), 5.42 (dd, *J*_7,6_ = 1.8, *J*_7,8_ = 4.6 Hz, 1H; H-7), 5.27 (ddd, *J*_8,9a_ = 2.5, *J*_8,7_ = 4.6, *J*_8,9b_ = 7.3 Hz, 1H; H-8), 4.65 (dd, *J*_9a,8_ = 2.5, *J*_9a,9b_ = 12.4 Hz, 1H; H-9a), 4.51 (dd, *J*_6,7_ = 1.8, *J*_6,5_ = 10.3 Hz, 1H; H-6), 4.41 (ddd, *J*_4,3_ = 2.3, *J*_4,5_ = *J*_4,NH_ = 9.7 Hz, 1H; H-4), 4.14 (dd, *J*_9b,8_ = 7.3, *J*_9b,9a_ = 12.4 Hz, 1H; H-9b), 4.11 (m, 1H; H-5), 3.79 (s, 3H; COOCH_3_), 3.01 (s, 3H; NHSO_2_CH_3_), 2.06 (s, 3H; OCOCH_3_), 2.05 (s, 3H; OCOCH_3_), 2.03 ppm (s, 3H; OCOCH_3_); ^13^C NMR (125 MHz, CDCl_3_): δ = 170.8, 170.7, 169.8 (3C; OCOCH_3_), 161.5 (C-1), 158.3 (q, *J*_C,F_ = 38.0 Hz, 1C; COCF_3_), 144.8 (C-2), 115.5 (q, *J*_C,F_ = 288.0 Hz, 1C; COCF_3_), 110.4 (C-3), 76.4 (C-6), 71.1 (C-8), 67.8 (C-7), 62.0 (C-9), 52.6 (COOCH_3_), 51.7 (C-4), 48.4 (C-5), 42.1 (NHSO_2_CH_3_), 20.7 (OCOCH_3_), 20.6 (OCOCH_3_), 20.4 ppm (OCOCH_3_); MS (ESI negative): *m*/*z* 561.2 [M – H]^−^; elemental analysis calcd (%) for C_19_H_25_F_3_N_2_O_12_S: C 40.57, H 4.48, N 4.98; found: C 40.65, H 4.43, N 5.09.

#### Preparation of Methyl-7,8,9-tri-*O*-acetyl-2,6-anhydro-4-(phenylsulfonamido)-3,4,5-trideoxy-5-(2,2,2-trifluoroacetamido)-d-glycero-d-galacto-non-2-enonate (**25**)

Starting from protected glycal **16**(13) (242 mg, 0.50 mmol), according to the general procedure and using benzenesulfonyl chloride (96 μL, 0.75 mmol), compound **25** was obtained (219 mg, 70%), after flash chromatographic purification (eluting with AcOEt/hexane, 4:6 v/v), as a white amorphous solid. Compound **25** showed: [α]_D_^23^ = +66.5 (*c* = 1.0 in chloroform); ^1^H NMR (500 MHz, CDCl_3_): δ = 7.84–7.79 (overlapping, 2H; Ph), 7.60 (m, 1H; Ph), 7.54–7.48 (overlapping, 2H; Ph), 7.24 (d, *J*_NH,5_ = 9.1 Hz, 1H; NHCOCF_3_), 5.68 (d, *J*_3,4_ = 2.5 Hz, 1H; H-3), 5.39 (dd, *J*_7,6_ = 2.0, *J*_7,8_ = 5.2 Hz, 1H; H-7), 5.33 (d, *J*_NH,4_ = 9.3 Hz, 1H; NHSO_2_C_6_H_5_), 5.27 (ddd, *J*_8,9a_ = 2.8, *J*_8,7_ = 5.2, *J*_8,9b_ = 6.8 Hz, 1H; H-8), 4.64 (dd, *J*_9a,8_ = 2.8, *J*_9a,9b_ = 12.5 Hz, 1H; H-9a), 4.53 (dd, *J*_6,7_ = 2.0, *J*_6,5_ = 10.2 Hz, 1H; H-6), 4.38 (ddd, *J*_4,3_ = 2.5, *J*_4,5_ = *J*_4,NH_ = 9.3 Hz, 1H; H-4), 4.15 (dd, *J*_9b,8_ = 6.8, *J*_9b,9a_ = 12.5 Hz, 1H; H-9b), 3.94 (m, 1H; H-5), 3.75 (s, 3H; COOCH_3_), 2.07 (s, 3H; OCOCH_3_), 2.04 (s, 3H; OCOCH_3_), 2.02 ppm (s, 3H; OCOCH_3_); ^13^C NMR (125 MHz, CDCl_3_): δ = 170.9, 170.6, 169.9 (3C; 3 × OCOCH_3_), 161.4 (C-1), 158.2 (q, *J*_C,F_ = 38.2 Hz, 1C; COCF_3_), 144.8 (C-2), 140.0, 133.3, 129.5, 126.9 (6C; Ph), 115.3 (q, *J*_C,F_ = 288.0 Hz, 1C; COCF_3_), 109.6 (C-3), 75.8 (C-6), 70.7 (C-8), 67.6 (C-7), 61.9 (C-9), 52.7 (COOCH_3_), 51.1, 49.0 (C-4 and C-5), 20.8 (OCOCH_3_), 20.7 (OCOCH_3_), 20.5 ppm (OCOCH_3_); MS (ESI negative): *m*/*z* 623.4 [M – H]^−^; elemental analysis calcd (%) for C_24_H_27_F_3_N_2_O_12_S: C 46.16, H 4.36, N 4.49; found: C 46.30, H 4.28, N 4.55.

#### Preparation of Methyl-7,8,9-tri-*O*-acetyl-2,6-anhydro-3,4,5-trideoxy-5-(2,2,2-trifluoroacetamido)-4-((trifluoromethyl)sulfonamido)-d-glycero-d-galacto-non-2-enonate (**26**)

Starting from protected glycal **16**(13) (242 mg, 0.50 mmol), according to the general procedure, adding trifluoromethanesulfonyl anhydride (126 μL, 0.75 mmol) at −78 °C and stirring for 1 h at the same temperature (instead of 23 °C), compound **26** was obtained (191 mg, 62%), after flash chromatographic purification (eluting with AcOEt/hexane, 3:7 v/v), as an amorphous white solid. Compound **26** showed: [α]_D_^23^ = +65.0 (*c* = 1.0 in chloroform); ^1^H NMR (500 MHz, CDCl_3_): δ = 7.65 (br s, 1H; NHCOCF_3_), 5.91 (d, *J*_3,4_ = 2.3 Hz, 1H; H-3), 5.45 (dd, *J*_7,6_ = 2.0, *J*_7,8_ = 4.5 Hz, 1H; H-7), 5.26 (ddd, *J*_8,9a_ = 2.3, *J*_8,7_ = 4.5, *J*_8,9b_ = 7.3 Hz, 1H; H-8), 4.71 (dd, *J*_9a,8_ = 2.3, *J*_9a,9b_ = 12.4 Hz, 1H; H-9a), 4.52 (dd, *J*_6,7_ = 2.0, *J*_6,5_ = 10.3 Hz, 1H; H-6), 4.47 (dd, *J*_4,3_ = 2.3, *J*_4,5_ = 9.6 Hz, 1H; H-4), 4.22–4.13 (overlapping, *J*_9b,8_ = 7.3, *J*_9b,9a_ = 12.4 Hz, 2H; H-9b and H-5), 3.82 (s, 3H; COOCH_3_), 2.09 (s, 3H; OCOCH_3_) 2.07 (s, 3H; OCOCH_3_), 2.05 ppm (s, 3H; OCOCH_3_); ^13^C NMR (125 MHz, CDCl_3_): δ = 171.5, 171.3, 169.8 (4C; 3 × OCOCH_3_ and NHCOCH_3_), 161.4 (C-1), 158.7 (q, *J*_C,F_ = 38.4 Hz, 1C; COCF_3_), 145.2 (C-2), 119.5 (q, *J*_C,F_ = 320.1 Hz, 1C; SO_2_CF_3_), 115.3 (q, *J*_C,F_ = 287.0 Hz, 1C; COCF_3_), 109.1 (C-3), 76.2 (C-6), 71.2 (C-8), 67.6 (C-7), 62.1 (C-9), 53.5 (C-4), 52.8 (COOCH_3_), 48.2 (C-5), 20.7 (OCOCH_3_), 20.5 (OCOCH_3_), 20.1 ppm (OCOCH_3_); MS (ESI negative): *m*/*z* 615.3 [M – H]^−^; elemental analysis calcd (%) for C_19_H_22_F_6_N_2_O_12_S: C 37.02, H 3.60, N 4.54; found: C 37.15, H 3.44, N 4.63.

#### Preparation of Methyl-4-acetamido-7,8,9-tri-*O*-acetyl-2,6-anhydro-3,4,5-trideoxy-5-(2,2,2-trifluoroacetamido)-d-glycero-d-galacto-non-2-enonate (**27**)

Starting from protected glycal **16**(13) (242 mg, 0.50 mmol), according to the general procedure and using acetyl chloride (53 μL, 0.75 mmol), compound **27** was obtained (190 mg, 72%), after flash chromatographic purification (eluting with AcOEt/hexane, 9:1 v/v), as a white amorphous solid. Compound **27** showed: [α]_D_^23^ = +30.0 (*c* = 1.0 in chloroform); ^1^H NMR (500 MHz, CDCl_3_): δ = 8.57 (d, *J*_NH,5_ = 9.9 Hz, 1H; NHCOCF_3_), 6.43 (d, *J*_NH,4_ = 9.7 Hz, 1H; NHCOCH_3_), 5.86 (d, *J*_3,4_ = 2.3 Hz, 1H; H-3), 5.47 (dd, *J*_7,6_ = 1.9, *J*_7,8_ = 4.5 Hz, 1H; H-7), 5.28 (ddd, *J*_8,9a_ = 2.6, *J*_8,7_ = 4.5, *J*_8,9b_ = 7.5 Hz, 1H; H-8), 5.19 (ddd, *J*_4,3_ = 2.3, *J*_4,5_ = *J*_4,NH_ = 9.7 Hz, 1H; H-4), 4.69 (dd, *J*_9a,8_ = 2.6, *J*_9a,9b_ = 12.4 Hz, 1H; H-9a), 4.49 (dd, *J*_6,7_ = 1.9, *J*_6,5_ = 10.2 Hz, 1H; H-6), 4.18 (m, 1H; H-5), 4.14 (dd, *J*_9b,8_ = 7.5, *J*_9b,9a_ = 12.4 Hz, 1H; H-9b), 3.78 (s, 3H; COOCH_3_), 2.04–2.03 (overlapping, 6H; 2 × OCOCH_3_), 2.03 (s, 3H; OCOCH_3_), 1.93 ppm (s, 3H; NHCOCH_3_); ^13^C NMR (125 MHz, CDCl_3_): δ = 172.0, 170.6, 170.3, 169.5 (4C; NHCOCH_3_ and 3 × OCOCH_3_), 161.5 (C-1), 158.4 (q, *J*_C,F_ = 38.0 Hz, 1C; COCF_3_), 144.9 (C-2), 115.5 (q, *J*_C,F_ = 287.2 Hz, 1C; COCF_3_), 110.3 (C-3), 76.8 (C-6), 71.3 (C-8), 67.8 (C-7), 62.1 (C-9), 52.5 (COOCH_3_), 47.7, 47.5 (C-4 and C-5), 22.6 (NHCOCH_3_), 20.8 (OCOCH_3_), 20.6 (OCOCH_3_), 20.4 ppm (OCOCH_3_). Other chemical–physical properties were superimposable with those previously reported in the literature.^29^

#### Preparation of Methyl-7,8,9-tri-*O*-acetyl-2,6-anhydro-4-benzamido-3,4,5-trideoxy-5-(2,2,2-trifluoroacetamido)-d-glycero-d-galacto-non-2-enonate (**28**)

Starting from protected glycal **16**(13) (242 mg, 0.50 mmol), according to the general procedure and using benzoyl chloride (87 μL, 0.75 mmol), compound **28** was obtained (209 mg, 71%), after flash chromatographic purification (eluting with AcOEt/hexane, 4:6 v/v), as a white amorphous solid. Compound **28** showed: [α]_D_^23^ = +79.0 (*c* = 1.0 in chloroform); ^1^H NMR (500 MHz, CDCl_3_): δ = 8.58 (d, *J*_NH,5_ = 9.9 Hz, 1H; NHCOCF_3_), 7.69–7.63 (overlapping, 2H; Ph), 7.49 (m, 1H; Ph), 7.42–7.35 (overlapping, 2H; Ph), 6.72 (d, *J*_NH,4_ = 9.6 Hz, 1H; NHCOC_6_H_5_), 5.99 (d, *J*_3,4_ = 2.2 Hz, 1H; H-3), 5.62 (dd, *J*_7,6_ = 1.7, *J*_7,8_ = 4.9 Hz, 1H; H-7), 5.52 (ddd, *J*_4,3_ = 2.2, *J*_4,5_ = *J*_4,NH_ = 9.6 Hz, 1H; H-4), 5.38 (ddd, *J*_8,9a_ = 2.4, *J*_8,7_ = 4.9, *J*_8,9b_ = 7.2 Hz, 1H; H-8), 4.73 (dd, *J*_9a,8_ = 2.4, *J*_9a,9b_ = 12.5 Hz, 1H; H-9a), 4.69 (dd, *J*_6,7_ = 1.7, *J*_6,5_ = 10.4 Hz, 1H; H-6), 4.41 (m, 1H; H-5), 4.19 (dd, *J*_9b,8_ = 7.2, *J*_9b,9a_ = 12.5 Hz, 1H; H-9b), 3.79 (s, 3H; COOCH_3_), 2.08 (s, 3H; OCOCH_3_), 2.04 (s, 3H; OCOCH_3_), 2.02 ppm (s, 3H; OCOCH_3_); ^13^C NMR (125 MHz, CDCl_3_): δ = 170.7, 170.2, 169.5 (4C; 3 × OCOCH_3_ and NHCOC_6_H_5_), 161.6 (C-1), 158.7 (q, *J*_C,F_ = 38.1 Hz, 1C; COCF_3_), 145.0 (C-2), 132.8, 132.4, 128.8, 127.0 (6C; Ph), 115.3 (q, *J*_C,F_ = 287.3 Hz, 1C; COCF_3_), 110.2 (C-3), 76.7 (C-6), 71.1 (C-8), 67.7 (C-7), 62.1 (C-9), 52.5 (COOCH_3_), 48.6, 47.4 (C-4 and C-5), 20.8 (OCOCH_3_), 20.7 (OCOCH_3_), 20.4 ppm (OCOCH_3_); MS (ESI negative): *m*/*z* 587.1 [M – H]^−^; elemental analysis calcd (%) for C_25_H_27_F_3_N_2_O_11_: C 51.02, H 4.62, N 4.76; found: C 51.17, H 4.66, N 4.64.

#### Preparation of Methyl-7,8,9-tri-*O*-acetyl-2,6-anhydro-4-(4-methylbenzamido)-3,4,5-trideoxy-5-(2,2,2-trifluoroacetamido)-d-glycero-d-galacto-non-2-enonate (**29**)

Starting from protected glycal **16**(13) (242 mg, 0.50 mmol), according to the general procedure and using *p*-toluoyl chloride (100 μL, 0.75 mmol), compound **29** was obtained (241 mg, 80%), after flash chromatographic purification (eluting with AcOEt/hexane, 4:6 v/v), as a white amorphous solid. Compound **29** showed: [α]_D_^23^ = +86.8 (*c* = 1.0 in chloroform); ^1^H NMR (500 MHz, CDCl_3_): δ = 8.51 (d, *J*_NH,5_ = 9.9 Hz, 1H; NHCOCF_3_), 7.57–7.55 (overlapping, 2H; Ph), 7.21–7.19 (overlapping, 2H; Ph), 6.47 (d, *J*_NH,4_ = 9.6 Hz, 1H; NHCOC_6_H_4_CH_3_), 5.99 (d, *J*_3,4_ = 2.2 Hz, 1H; H-3), 5.62 (dd, *J*_7,6_ = 1.7, *J*_7,8_ = 5.0 Hz, 1H; H-7), 5.50 (ddd, *J*_4,3_ = 2.2, *J*_4,5_ = *J*_4,NH_ = 9.6 Hz, 1H; H-4), 5.39 (ddd, *J*_8,9a_ = 2.5, *J*_8,7_ = 5.0, *J*_8,9b_ = 7.2 Hz, 1H; H-8), 4.74 (dd, *J*_9a,8_ = 2.5, *J*_9a,9b_ = 12.5 Hz, 1H; H-9a), 4.70 (dd, *J*_6,7_ = 1.7, *J*_6,5_ = 10.3 Hz, 1H; H-6), 4.39 (m, 1H; H-5), 4.20 (dd, *J*_9b,8_ = 7.2, *J*_9b,9a_ = 12.5 Hz, 1H; H-9b), 3.81 (s, 3H; COOCH_3_), 2.37 (s, 3H; PhCH_3_), 2.10 (s, 3H; OCOCH_3_), 2.05 (s, 3H; OCOCH_3_), 2.04 ppm (s, 3H; OCOCH_3_);^13^C NMR (125 MHz, CDCl_3_): δ = 170.6, 170.2, 169.6, 169.4 (4C; 3 × OCOCH_3_ and NHCOC_6_H_4_CH_3_), 161.6 (C-1), 158.7 (q, *J*_C,F_ = 38.0 Hz, 1C; COCF_3_), 145.1 (C-2), 143.1, 129.9, 129.5, 127.0 (6C; Ph), 115.3 (q, *J*_C,F_ = 287.2 Hz, 1C; COCF_3_), 110.1 (C-3), 76.8 (C-6), 71.1 (C-8), 67.8 (C-7), 62.1 (C-9), 52.6 (COOCH_3_), 48.5, 47.6 (C-4 and C-5), 21.5 (PhCH_3_), 20.8 (OCOCH_3_), 20.7 (OCOCH_3_), 20.4 ppm (OCOCH_3_); MS (ESI negative): *m*/*z* 601.3 [M – H]^−^; elemental analysis calcd (%) for C_26_H_29_F_3_N_2_O_11_: C 51.83, H 4.85, N 4.65; found: C 51.64, H 4.80, N 4.74.

#### Preparation of Methyl-7,8,9-tri-*O*-acetyl-2,6-anhydro-3,4,5-trideoxy-4,5-di-(2,2,2-trifluoroacetamido)-d-glycero-d-galacto-non-2-enonate (**30**)

Starting from protected glycal **16**(13) (242 mg, 0.50 mmol), according to the general procedure and using trifluoroacetic anhydride (104 μL, 0.75 mmol), compound **30** was obtained (209 mg, 72%), after flash chromatographic purification (eluting with AcOEt/hexane, 6:4 v/v), as a white amorphous solid. Compound **30** showed: [α]_D_^23^ = +14.1 (*c* = 1.0 in chloroform); ^1^H NMR (500 MHz, CDCl_3_): δ = 7.94 (d, *J*_NH,H_ = 9.8 Hz, 1H; NHCOCF_3_ at C-4 or C-5), 7.43 (d, *J*_NH,H_ = 9.2 Hz, 1H; NHCOCF_3_ at C-4 or C-5), 5.89 (d, *J*_3,4_ = 2.3 Hz, 1H; H-3), 5.48 (dd, *J*_7,6_ = 1.9, *J*_7,8_ = 4.7 Hz, 1H; H-7), 5.29 (ddd, *J*_8,9a_ = 2.5, *J*_8,7_ = 4.7, *J*_8,9b_ = 7.4 Hz, 1H; H-8), 5.05 (dd, *J*_4,3_ = 2.3, *J*_4,5_ = 9.6 Hz, 1H; H-4), 4.70 (dd, *J*_9a,8_ = 2.5, *J*_9a,9b_ = 12.5 Hz, 1H; H-9a), 4.51 (dd, *J*_6,7_ = 1.9, *J*_6,5_ = 10.4 Hz, 1H; H-6), 4.33 (m, 1H; H-5), 4.13 (dd, *J*_9b,8_ = 7.4, *J*_9b,9a_ = 12.5 Hz, 1H; H-9b), 3.80 (s, 3H; COOCH_3_), 2.07–2.05 (overlapping, 6H; 2 × OCOCH_3_) 2.04 ppm (s, 3H; OCOCH_3_); ^13^C NMR (125 MHz, CDCl_3_): δ = 171.0, 170.9, 169.7 (3C; 3 × OCOCH_3_), 161.3 (C-1), 158.5 (q, *J*_C,F_ = 38.3 Hz, 1C; COCF_3_ at C-4 or C-5), 158.4 (q, *J*_C,F_ = 38.1 Hz, 1C; COCF_3_ at C-4 or C-5), 145.5 (C-2), 115.4 (q, *J*_C,F_ = 287.2 Hz, 1C; COCF_3_ at C-4 or C-5), 115.2 (q, *J*_C,F_ = 287.5 Hz, 1C; COCF_3_ at C-4 or C-5), 108.1 (C-3), 76.4 (C-6), 71.2 (C-8), 67.5 (C-7), 62.0 (C-9), 52.7 (COOCH_3_), 48.7, 47.4 (C-4 and C-5), 20.7 (OCOCH_3_), 20.6 (OCOCH_3_), 20.2 ppm (OCOCH_3_); MS (ESI negative): *m*/*z* 579.2 [M – H]^−^; elemental analysis calcd (%) for C_20_H_22_F_6_N_2_O_11_: C 41.39, H 3.82, N 4.83; found: C 41.22, H 3.88, N 4.74.

#### Preparation of Methyl-5-acetamido-7,8,9-tri-*O*-acetyl-2,6-anhydro-4-(4-methylbenzamido)-3,4,5-trideoxy-d-glycero-d-galacto-non-2-enonate (**32**)

Starting from protected glycal **15**(8) (215 mg, 0.50 mmol), according to the general procedure and using *p*-toluoyl chloride (100 μL, 0.75 mmol), compound **32** was obtained (217 mg, 79%), after flash chromatographic purification (eluting with AcOEt/hexane, 8:2 v/v), as a white amorphous solid. Compound **32** showed: [α]_D_^23^ = +113.6 (*c* = 1.0 in chloroform); ^1^H NMR (500 MHz, CDCl_3_): δ = 7.65–7.60 (overlapping, 2H; Ph), 7.21–7.14 (overlapping, 3H; Ph and NHCOCH_3_ or NHCOC_6_H_4_CH_3_), 6.83 (d, *J*_NH,4_ = 8.5 Hz, 1H; NHCOCH_3_ or NHCOC_6_H_4_CH_3_), 6.01 (d, *J*_3,4_ = 2.2 Hz, 1H; H-3), 5.61 (dd, *J*_7,6_ = 1.8, *J*_7,8_ = 4.7 Hz, 1H; H-7), 5.36 (ddd, *J*_8,9a_ = 2.5, *J*_8,7_ = 4.7, *J*_8,9b_ = 7.4 Hz, 1H; H-8), 5.08 (m, 1H; H-4), 4.72 (dd, *J*_9a,8_ = 2.5, *J*_9a,9b_ = 12.4 Hz, 1H; H-9a), 4.46 (dd, *J*_6,7_ = 1.8, *J*_6,5_ = 10.4 Hz, 1H; H-6), 4.36 (m, 1H; H-5), 4.20 (dd, *J*_9b,8_ = 7.4, J_9b,9a_ = 12.4 Hz, 1H; H-9b), 3.77 (s, 3H; COOCH_3_), 2.34 (s, 3H; PhCH_3_), 2.09 (s, 3H; OCOCH_3_), 2.05 (s, 6H; 2 × OCOCH_3_), 1.80 ppm (s, 3H; NHCOOCH3); ^13^C NMR (125 MHz, CDCl_3_): δ = 171.6, 170.6, 170.3, 169.9, 168.5 (5C; 3 × OCOCH_3_, NHCOCH_3_ and NHCOC_6_H_4_CH_3_), 161.8 (C-1), 144.4 (C-2), 142.6, 130.5, 129.4, 127.0 (6C; Ph), 110.9 (C-3), 77.2 (C-6), 71.4 (C-8), 68.1 (C-7), 62.3 (C-9), 52.4 (COOCH_3_), 49.7, 46.5 (C-4 and C-5), 22.7 (NHCOCH_3_), 21.4 (PhCH_3_), 20.8 (OCOCH_3_), 20.7 (OCOCH_3_), 20.6 ppm (OCOCH_3_); MS (ESI negative): *m*/*z* 547.5 [M – H]^−^; elemental analysis calcd (%) for C_26_H_32_N_2_O_11_: C 56.93, H 5.88, N 5.11; found: C 56.99, H 5.73, N 5.26.

#### General Procedure of Deacetylation and Selective Removal of Esteric Function

Each glycal **17**, **18**, **25**–**30**, **32** (0.20 mmol) was treated with a methanolic solution of NaOMe, freshly prepared by dissolving sodium metal (5 mg, 0.22 mmol) in anhydrous MeOH (2 mL). Each reaction mixture was stirred at 23 °C for 1 h and then quenched with acidic resin (Dowex 50WX8, H^+^). Then, the resin was filtered off and washed with MeOH (2 mL × 3) and the organic phase was evaporated under reduced pressure. The crude compound was purified by flash chromatography and directly subjected to hydrolysis in a methanol–water solution (1.5 mL, 2:1 v/v) containing Et_3_N (0.90 mL), at 23 °C overnight. Then, the mixture was treated with acidic resin (Dowex 50WX8, H^+^) until acidic pH and the resin was filtered off and washed with MeOH (2 mL × 3). Finally, the solvent was removed under reduced pressure and the residue was purified by preparative HPLC, and the desired free glycal was achieved after lyophilization.

#### Preparation of 5-Acetamido-2,6-anhydro-4-methylsulfonamido-3,4,5-trideoxy-d-glycero-d-galacto-non-2-enoic Acid (**13**)

Starting from protected glycal **17** (102 mg, 0.20 mmol), according to the general two-step procedure Zemplén reaction followed by hydrolysis, compound **13** was obtained (49 mg, 66%) as a white amorphous solid, showing: ^1^H NMR (500 MHz, MeOD–D_2_O 1:1): δ = 5.96 (d, *J*_3,4_ = 2.4 Hz, 1H; H-3), 4.41–4.33 (overlapping, 2H; H-6 and H-4), 4.06 (m, 1H; H-5), 3.89 (ddd, *J*_8,9a_ = 2.6, *J*_8,9b_ = 5.6, *J*_8,7_ = 9.6, Hz, 1H; H-8), 3.86 (dd, *J*_9a,8_ = 2.6, *J*_9a,9b_ = 11.8 Hz, 1H; H-9a), 3.69–3.63 (overlapping, 2H; H-9b and H-7), 3.09 (s, 3H; NHSO_2_C*H*_3_), 2.06 ppm (s, 3H; NHCOC*H*_3_); ^13^C NMR (125 MHz, MeOD–D_2_O 1:1): δ = 175.3 (NH*C*OCH_3_), 166.0 (C-1), 145.9 (C-2), 111.7 (C-3), 77.8 (C-6), 71.0 (C-8), 69.4 (C-7), 64.3 (C-9), 52.7, 49.3 (C-4 and C-5), 42.1 (NHSO_2_*C*H_3_), 23.1 ppm (N*H*COCH_3_); MS (ESI negative): *m*/*z* 367.1 [M – H]^−^. MS spectrum value is in agreement with that previously reported in the literature.^28^

#### Preparation of 2,6-Anhydro-4-methylsulfonamido-3,4,5-trideoxy-5-(2,2,2-trifluoroacetamido)-d-glycero-d-galacto-non-2-enoic Acid (**14**)

Starting from protected glycal **18** (112 mg, 0.20 mmol), according to the general two-step procedure Zemplén reaction followed by hydrolysis, compound **14** was obtained (58 mg, 69%) as a white amorphous solid, showing: [α]_D_^23^ = +37.2 (*c* = 1.0 in methanol–water 1:1); ^1^H NMR (500 MHz, MeOD–D_2_O 1:1): δ = 6.01 (d, *J*_3,4_ = 2.2 Hz, 1H; H-3), 4.55 (d, *J*_6,5_ = 10.8 Hz, 1H; H-6), 4.45 (dd, *J*_4,3_ = 2.2, *J*_4,5_ = 9.7 Hz, 1H; H-4), 4.22 (m, 1H; H-5), 3.91 (ddd, *J*_8,9a_ = 2.6, *J*_8,9b_ = 6.0, *J*_8,7_ = 9.7 Hz, 1H; H-8), 3.86 (dd, *J*_9a,8_ = 2.6, *J*_9a,9b_ = 11.9 Hz, 1H; H-9a), 3.69–3.61 (overlapping, 2H; H-9b and H-7), 3.10 ppm (s, 3H; NHSO_2_CH_3_); ^13^C NMR (125 MHz, MeOD–D_2_O 1:1): δ = 165.9 (C-1), 159.9 (q, *J*_C,F_ = 38.0 Hz, 1C; COCF_3_), 145.9 (C-2), 116.7 (q, *J*_C,F_ = 287.0 Hz, 1C; COCF_3_), 111.4 (C-3), 77.1 (C-6), 71.0 (C-8), 69.2 (C-7), 64.1 (C-9), 52.3, 49.9 (C-4 and C-5), 42.0 (NHSO_2_CH_3_); MS (ESI negative): *m*/*z* 421.1 [M – H]^−^; elemental analysis calcd (%) for C_12_H_17_F_3_N_2_O_9_S: C 34.13, H 4.06, N 6.63; found: C 34.01, H 4.22, N 6.55.

#### Preparation of 2,6-Anhydro-4-(phenylsulfonamido)-3,4,5-trideoxy-5-(2,2,2-trifluoroacetamido)-d-glycero-d-galacto-non-2-enoic Acid (**19**)

Starting from protected glycal **25** (125 mg, 0.20 mmol), according to the general two-step procedure Zemplén reaction followed by hydrolysis, compound **19** was obtained (64 mg, 66%) as a white amorphous solid, showing: [α]_D_^23^ = +24.0 (*c* = 1.0 in dimethyl sulfoxide); ^1^H NMR (500 MHz, MeOD–D_2_O 1:1): δ = 7.90–7.86 (overlapping, 2H; Ph), 7.72 (m, 1H; Ph), 7.66–7.61 (overlapping, 2H; Ph), 5.43 (d, *J*_3,4_ = 2.3 Hz, 1H; H-3), 4.47 (br d, *J*_6,5_ = 10.7 Hz, 1H; H-6), 4.31 (d, *J*_4,3_ = 2.3, *J*_4,5_ = 9.7 Hz, 1H; H-4), 4.18 (m, 1H; H-5), 3.87 (ddd, *J*_8,9a_ = 2.6, *J*_8,9b_ = 5.8, *J*_8,7_ = 9.4 Hz, 1H; H-8), 3.84 (dd, *J*_9a,8_ = 2.6, *J*_9a,9b_ = 11.8 Hz, 1H; H-9a), 3.63 (dd, *J*_9b,8_ = 5.8, *J*_9b,9a_ = 11.8 Hz, 1H; H-9b), 3.58 ppm (dd, *J*_7,6_ = 1.0, *J*_7,8_ = 9.4 Hz, 1H; H-7); ^13^C NMR (125 MHz, MeOD–D_2_O 1:1): δ = 165.0 (C-1), 146.3 (C-2), 143.1, 133.8, 130.4, 127.9 (6C; Ph), 110.6 (C-3), 77.7 (C-6), 71.3 (C-8), 69.9 (C-7), 64.7 (C-9), 52.5, 49.9 ppm (C-4 and C-5); MS (ESI negative): *m*/*z* 483.2 [M – H]^−^; elemental analysis calcd (%) for C_17_H_19_F_3_N_2_O_9_S: C 42.15, H 3.95, N 5.78; found: C 42.32, H 3.87, N 5.89.

#### Preparation of 2,6-Anhydro-3,4,5-trideoxy-5-(2,2,2-trifluoroacetamido)-4-((trifluoromethyl)sulfonamido)-d-glycero-d-galacto-non-2-enoic Acid (**20**)

Starting from protected glycal **26** (123 mg, 0.20 mmol), according to the general two-step procedure Zemplén reaction followed by hydrolysis, compound **20** was obtained (53 mg, 56%) as a white amorphous solid, showing: [α]_D_^23^ = +33.0 (*c* = 1.0 in dimethyl sulfoxide); ^1^H NMR (500 MHz, MeOD): δ = 5.83 (d, *J*_3,4_ = 2.2 Hz, 1H; H-3), 4.49 (d app, *J*_6,5_ = 10.7 Hz, 1H; H-6), 4.45 (d app, *J*_4,5_ = 9.5 Hz, 1H; H-4), 4.32 (m, 1H; H-5), 3.88 (ddd, *J*_8,9a_ = 2.8, *J*_8,9b_ = 5.4, *J*_8,7_ = 9.4 Hz, 1H; H-8), 3.82 (dd, *J*_9a,8_ = 2.8, *J*_9a,9b_ = 11.5 Hz, 1H; H-9a), 3.66 (dd, *J*_9b,8_ = 5.4, *J*_9b,9a_ = 11.5 Hz, 1H; H-9b) 3.59 ppm (d, *J*_7,8_ = 9.4 Hz, 1H; H-7); ^13^C NMR (125 MHz, MeOD): δ = 164.8 (C-1), 159.3 (q, *J*_C,F_ = 37.5 Hz, 1C; COCF_3_), 147.1 (C-2), 121.2 (q, *J*_C,F_ = 320.0 Hz, 1C; SO_2_CF_3_), 117.4 (q, *J*_C,F_ = 287.0 Hz, 1C; COCF_3_), 109.7 (C-3), 77.8 (C-6), 71.3 (C-8), 69.9 (C-7), 64.7 (C-9), 54.4, 49.7 ppm (C-4 and C-5); MS (ESI negative): *m*/*z* 475.2 [M – H]^−^; elemental analysis calcd (%) for C_12_H_14_F_6_N_2_O_9_S: C 30.26, H 2.96, N 5.88; found: C 30.18, H 2.99, N 5.69.

#### Preparation of 4-Acetamido-2,6-anhydro-3,4,5-trideoxy-5-(2,2,2-trifluoroacetamido)-d-glycero-d-galacto-non-2-enoic Acid (**21**)

Starting from protected glycal **27** (105 mg, 0.20 mmol), according to the general two-step procedure Zemplén reaction followed by hydrolysis, compound **21** was obtained (45 mg, 58%) as a white amorphous solid, showing: [α]_D_^23^ = −6.4 (*c* = 1.0 in methanol–water 1:1); ^1^H NMR (500 MHz, MeOD): δ = 5.88 (d, *J*_3,4_ = 2.5 Hz, 1H; H-3), 4.94 (dd, *J*_4,3_ = 2.5, *J*_4,5_ = 9.8 Hz, 1H; H-4), 4.55 (dd, *J*_6,7_ = 1.2, *J*_6,5_ = 10.7 Hz, 1H; H-6), 4.29 (m, 1H; H-5), 3.92 (ddd, *J*_8,9a_ = 2.7, *J*_8,9b_ = 6.0, *J*_8,7_ = 9.4, Hz, 1H; H-8), 3.86 (dd, *J*_9a,8_ = 2.7, *J*_9a,9b_ = 11.9 Hz, 1H; H-9a), 3.68–3.61 (overlapping, 2H; H-9b and H-7), 1.96 ppm (s, 3H; NHCOC*H*_3_); ^13^C NMR (125 MHz, MeOD): δ = 174.6 (NH*C*OCH_3_), 165.9 (C-1), 159.9 (q, *J*_C,F_ = 37.6 Hz, 1C; *C*OCF_3_), 145.9 (C-2), 116.8 (q, *J*_C,F_ = 286.6 Hz, 1C; CO*C*F_3_), 111.4 (C-3), 76.9 (C-6), 71.1 (C-8), 69.1 (C-7), 64.2 (C-9), 49.6, 48.6 (C-4 and C-5), 22.7 ppm (NHCO*C*H_3_); MS (ESI negative): *m*/*z* 385.1 [M – H]^−^; elemental analysis calcd (%) for C_13_H_17_F_3_N_2_O_8_: C 40.42, H 4.44, N 7.25; found: C 40.54, H 4.60, N 7.17.

#### Preparation of 2,6-Anhydro-4-benzamido-3,4,5-trideoxy-5-(2,2,2-trifluoroacetamido)-d-glycero-d-galacto-non-2-enoic Acid (**22**)

Starting from protected glycal **28** (118 mg, 0.20 mmol), according to the general two-step procedure Zemplén reaction followed by hydrolysis, compound **22** was obtained (49 mg, 55%) as a white amorphous solid, showing: [α]_D_^23^ = +65.0 (*c* = 1.0 in methanol–water 3:1); ^1^H NMR (500 MHz, MeOD–D_2_O 3:1): δ = 7.73–7.69 (overlapping, 2H; Ph), 7.59 (m, 1H; Ph), 7.53–7.47 (overlapping, 2H; Ph), 5.95 (d, *J*_3,4_ = 2.4 Hz, 1H; H-3), 5.18 (dd, *J*_4,3_ = 2.4, *J*_4,5_ = 9.8 Hz, 1H; H-4), 4.60 (d app, *J*_6,5_ = 10.8 Hz, 1H; H-6), 4.51 (m, 1H; H-5), 3.95 (ddd, *J*_8,9a_ = 2.7, *J*_8,9b_ = 5.9, *J*_8,7_ = 9.3, Hz, 1H; H-8), 3.88 (dd, *J*_9a,8_ = 2.7, *J*_9a,9b_ = 11.9 Hz, 1H; H-9a), 3.70–3.64 ppm (overlapping, 2H; H-9b and H-7); ^13^C NMR (125 MHz, MeOD–D_2_O 3:1): δ = 171.5 (NH*C*OC_6_H_5_), 166.5 (C-1), 159.9 (q, *J*_C,F_ = 37.7 Hz, 1C; *C*OCF_3_), 146.5 (C-2), 134.5, 133.2, 129.7, 128.1 (6C; Ph), 116.8 (q, *J*_C,F_ = 286.6 Hz, 1C; CO*C*F_3_), 110.8 (C-3), 76.9 (C-6), 71.1 (C-8), 69.2 (C-7), 64.2 (C-9), 49.9–48.3 ppm (2C overlapping to solvent; C-4 and C-5); MS (ESI negative): *m*/*z* 447.1 [M – H]^−^; elemental analysis calcd (%) for C_18_H_19_F_3_N_2_O_8_: C 48.22, H 4.27, N 6.25; found: C 48.16, H 4.33, N 6.37.

#### Preparation of 2,6-Anhydro-4-(4-methylbenzamido)-3,4,5-trideoxy-5-(2,2,2-trifluoroacetamido)-d-glycero-d-galacto-non-2-enoic Acid (**23**)

Starting from protected glycal **29** (121 mg, 0.20 mmol), according to the general two-step procedure Zemplén reaction followed by hydrolysis, compound **23** was obtained (53 mg, 57%) as a white amorphous solid, showing: [α]_D_^23^ = +69.8 (*c* = 1.0 in methanol–water 2:1); ^1^H NMR (500 MHz, DMSO-d_6_): δ = 9.39 (d, *J*_NH,5_ = 8.7 Hz, 1H; NHCOCF_3_), 8.52 (d, *J*_NH,4_ = 9.0 Hz, 1H; NHCOC_6_H_4_CH_3_), 7.71–7.65 (overlapping, 2H; Ph), 7.27–7.22 (overlapping, 2H; Ph), 5.67 (d, *J*_3,4_ = 2.4 Hz, 1H; H-3), 5.05 (ddd, *J*_4,3_ = 2.4, *J*_4,5_ = *J*_4,NH_ = 9.0 Hz, 1H; H-4), 4.84–4.50 (overlapping, 2H; 2 × OH), 4.48–4.33 (overlapping, 2H; H-5 and H-6), 4.08 (br s, 1H; OH), 3.69 (ddd, *J*_8,9a_ = 2.8, *J*_8,9b_ = 5.6, *J*_8,7_ = 9.3 Hz, 1H; H-8), 3.64 (dd, *J*_9a,8_ = 2.8, J_9a,9b_ = 11.3 Hz, 1H; H-9a), 3.46–3.39 (overlapping, 2H; H-9b and H-7), 2.34 ppm (s, 3H; PhCH_3_); ^13^C NMR (125 MHz, DMSO-d_6_): δ = 166.5 (NHCOC_6_H_4_CH_3_), 162.9 (C-1), 156.1 (q, *J*_C,F_ = 36.1 Hz, 1C; COCF_3_), 145.0 (C-2), 140.9, 131.5, 128.5, 127.2 (6C; Ph), 115.6 (q, *J*_C,F_ = 288.9 Hz, 1C; COCF_3_), 109.9 (C-3), 76.0 (C-6), 69.7 (C-8), 68.2 (C-7), 63.4 (C-9), 47.8 and 47.5 (C-4 and C-5), 20.8 ppm (1C; PhCH_3_); MS (ESI negative): *m*/*z* 461.2 [M – H]^−^; elemental analysis calcd (%) for C_19_H_21_F_3_N_2_O_8_: C 49.36, H 4.58, N 6.06; found: C 49.22, H 4.52, N 6.01.

#### Preparation of 2,6-Anhydro-3,4,5-trideoxy-4,5-di-(2,2,2-trifluoroacetamido)-d-glycero-d-galacto-non-2-enoic Acid (**24**)

Starting from protected glycal **30** (116 mg, 0.20 mmol), according to the general two-step procedure Zemplén reaction followed by hydrolysis, compound **24** was obtained (54 mg, 61%) as a white amorphous solid, showing: [α]_D_^23^ = −23.0 (*c* = 1.0 in methanol); ^1^H NMR (500 MHz, MeOD): δ = 5.82 (d, *J*_3,4_ = 2.1 Hz, 1H; H-3), 5.03 (dd, *J*_4,3_ = 2.1, *J*_4,5_ = 9.5 Hz, 1H; H-4), 4.54 (d app, *J*_6,5_ = 10.7 Hz, 1H; H-6), 4.43 (m, 1H; H-5), 3.88 (ddd, *J*_8,9a_ = 2.6, *J*_8,9b_ = 5.4, *J*_8,7_ = 9.4 Hz, 1H; H-8), 3.82 (dd, *J*_9a,8_ = 2.6, *J*_9a,9b_ = 11.5 Hz, 1H; H-9a), 3.66 (dd, *J*_9b,8_ = 5.4, *J*_9b,9a_ = 11.5 Hz, 1H; H-9b), 3.57 ppm (d app, *J*_7,8_ = 9.4 Hz, 1H; H-7); ^13^C NMR (125 MHz, MeOD): δ = 165.1 (C-1), 159.4 (q, *J*_C,F_ = 37.5 Hz, 1C; *C*OCF_3_ at C-4 or at C-5), 159.3 (q, *J*_C,F_ = 37.5 Hz, 1C; *C*OCF_3_ at C-4 or at C-5), 147.1 (C-2), 117.2 (overlapping, *J*_C,F_ = 287.0 Hz, 2C; CO*C*F_3_ at C-4 and at C-5_)_, 109.4 (C-3), 77.6 (C-6), 71.4 (C-8), 69.7 (C-7), 64.8 (C-9), 49.5, 49.3 ppm (C-4 and C-5); MS (ESI negative): *m*/*z* 439.2 [M – H]^−^; elemental analysis calcd (%) for C_13_H_14_F_6_N_2_O_8_: C 35.47, H 3.21, N 6.36; found: C 35.61, H 3.27, N 6.22.

#### Preparation of 5-Acetamido-2,6-anhydro-4-(4-methylbenzamido)-3,4,5-trideoxy-d-glycero-d-galacto-non-2-enoic Acid (**31**)

Starting from protected glycal **32** (110 mg, 0.20 mmol), according to the general two-step procedure Zemplén reaction followed by hydrolysis, compound **31** was obtained (45 mg, 52%) as a white amorphous solid, showing: ^1^H NMR (500 MHz, MeOD–D_2_O 1:1): δ = 7.70–7.58 (overlapping, 2H; Ph), 7.37–7.26 (overlapping, 2H; Ph), 5.91 (m, 1H; H-3), 5.04 (m, 1H; H-4), 4.42 (m, 1H; H-6), 4.35 (m, 1H; H-5), 3.93 (m, 1H; H-8), 3.87 (m, 1H; H-9a), 3.73–3.63 (overlapping, 2H; H-7 and H-9b), 2.38 (s, 3H; PhCH_3_), 1.96 ppm (s, 3H; NHCOC*H*_3_); ^1^H NMR (500 MHz, DMSO-d_6_): δ = 8.50 (d, *J*_NH,4_ = 8.1 Hz, 1H; N*H*COC_6_H_4_CH_3_ or N*H*COCH_3_), 8.14 (d, *J*_NH,5_ = 8.4 Hz, 1H; N*H*COC_6_H_4_CH_3_ or N*H*COCH_3_), 7.75–7.69 (overlapping, 2H; Ph), 7.28–7.22 (overlapping, 2H; Ph), 5.67 (d, *J*_3,4_ = 2.1 Hz, 1H; H-3), 4.89 (m, 1H; H-4), 4.16 (m, 1H; H-5), 4.08 (d, *J*_6,5_ = 10.7 Hz, 1H; H-6), 3.73–3.39 (overlapping, 4H; H-7, H-8, H-9a, H-9b and residual water), 2.34 (s, 3H; PhCH_3_), 1.83 ppm (s, 3H; NHCOC*H*_3_); ^13^C NMR (125 MHz, DMSO-d_6_): δ = 171.6, 166.8, 163.3 (3C; NH*C*OCH_3_, NH*C*OC_6_H_4_CH_3_ and C-1), 145.3 (C-2), 141.3, 131.4, 128.8, 127.3, 127.2 (6C; Ph), 109.7 (C-3), 77.1 (C-6), 69.5 (C-8), 68.5 (C-7), 63.6 (C-9), 47.9, 47.0 (C-4 and C-5), 22.6 (NHCO*C*H_3_), 20.9 ppm (Ph*C*H_3_); MS (ESI negative): *m*/*z* 407.1 [M – H]^−^; elemental analysis calcd (%) for C_19_H_24_N_2_O_8_: C 55.88, H 5.92, N 6.86; found: C 55.96, H 5.80, N 6.81.

### Cells and Viruses

African green monkey kidney (Vero) cells were grown in minimal essential medium (MEM) (Thermo Fisher Scientific) supplemented with 10% (v/v) fetal calf serum (FCS) (Sigma) and antibiotics (1% v/v penicillin/streptomycin, EuroClone) and were maintained at 37 °C in a humidified atmosphere supplemented with 5% CO_2_.

NDV La Sota “Clone 30” was grown and purified as described previously in the literature,^30^ La Sota 40/14 (inactivated) and the velogenic (inactivated) strain APMV-1/chicken/Egypt/13VIR-5009–2/2013 were obtained from Istituto Zooprofilattico Sperimentale delle Venezie. Stock viruses were harvested, titrated, and stored at −80 °C until use.

### Neuraminidase Activity Assay

Neuraminidase activity inhibition (NI) assay was performed, according to Venerando et al.,^31^ using 4-MUNeu5Ac as the artificial substrate. Briefly, the incubation mixture (final volume of 100 μL) contained 0.2 μg of NDV, different amounts of the inhibitors (0–2.0 mM), 0.12 mM 4-MU-Neu5Ac, 600 μg of bovine serum albumin (BSA) and 200 mM sodium citrate/phosphate buffer pH 6.8. After incubation at 37 °C for 15 min, the reactions were stopped by the addition of 1.5 mL of 0.2 M glycine buffered with NaOH at pH 10.8, and the neuraminidase activity was determined by spectrofluorometric measurement (Varioskan LUX Multimode Microplate reader, Thermo Fisher Scientific) of the 4-methylumbelliferone released (λ excitation 365 nm, λ emission 448 nm). One mU of neuraminidase activity is defined as the amount of enzyme releasing 1 nmol of *N*-acetylneuraminic acid per minute at 37 °C. Eight concentrations of each inhibitor were used to determine the IC_50_ with a fixed concentration (0.12 mM) of 4-MUNeu5Ac.

### Cytotoxicity Assay

The cytotoxicity of the compounds was evaluated by AlamarBlue reduction assay. *Vero cells* were seeded in 96-well plates at an initial density of 2 × 10^4^ cells per well. The cells were incubated with increasing concentrations of the compounds for 48 h at 37 °C and 5% CO_2_. AlamarBlue HS Cell Viability Reagent (Thermo Fisher Scientific) was added to the cells, which were further incubated for 2 h. The optical density was measured at 570 nm and 600 nm and the percentage of viable cells was calculated according to manufacturer’s instructions.

### Virus Titration by Focus Forming Assay (FFA)

Supernatants of *Vero cells* cultures collected in the VYRA were serially diluted in MEM plus 1 μg/mL l-1-tosylamido-2-phenylethyl chloromethyl ketone (TPCK)-treated trypsin and incubated on confluent monolayers of *Vero cells*, in 96-well plates, for 1 h at 37 °C. After infection, the inoculum was removed and an overlay of 1.25% Avicel microcrystalline cellulose and 1 μg/mL TPCK-treated trypsin was added. After 24 h, the overlay medium was removed and cells were washed once with phosphate-buffered saline (PBS) and fixed in PBS 4% paraformaldehyde (PFA), for 30 min at 4 °C. Upon removal, the cells were permeabilized by incubation with a 0.5% Triton X-100 solution for 10 min.

### Microplaque Staining

Immunostaining of infected cells was performed by incubation of monoclonal antibody against NDV (1:4000; MyBioSource) for 1 h, followed by 1 h incubation with peroxidase-labeled goat anti-mouse antibodies (1:2000; DAKO) and a 5 min incubation with the True Blue (KPL) peroxidase substrate. Solution of 1% bovine serum albumin and 0.1% Tween-20 in PBS was used for the preparation of working dilutions of immuno-reagents. After each antibody incubation, the cells were washed four times through a 5 min incubation with a 0.1% Tween-20 PBS solution. Microplaques were counted and measured with the software Fiji after acquisition of pictures at a high resolution of 4800 × 9400 dpi, on a flatbed scanner.

### Plaque Reduction Assay (PRA)

Confluent monolayer of *Vero cells* in 96-well plates was first washed with PBS and then infected with 15–20 plaque-forming units (PFU) per well of NDV La Sota virus in MEM supplemented with 1 μg/mL TPCK-treated trypsin in the presence of different concentrations of test compounds. After 1 h of incubation at 37 °C, the cells were incubated with a medium containing 1.25% Avicel microcrystalline cellulose and 1 μg/mL TPCK-treated trypsin. At 48 h post-infection (p.i.), cell monolayers were fixed with 4% formaldehyde solution and stained for microplaque.

### Viral Binding Inhibition Assay

Different concentrations of test compounds were preincubated with 15–20 PFU per well of NDV La Sota virus for 1 h at 37 °C. After this time, confluent monolayers of *Vero cells* in 96-well plates were first washed with PBS and then infected with the virus/compounds mix s. After 1 h of incubation at 37 °C, the inoculum was washed out and replaced with MEM supplemented with 1.25% Avicel microcrystalline cellulose and 1 μg/mL TPCK-treated trypsin. At 24 h p.i., cell monolayers were fixed with 4% formaldehyde solution and stained for microplaque assay.

### Viral Release Inhibition Assay

Confluent monolayer of *Vero cells* in 96-well plates was first washed with PBS and then infected with 15–20 PFU per well of NDV La Sota virus in MEM supplemented with 1 μg/mL TPCK-treated trypsin. After 1 h of incubation at 37 °C, the inoculum was washed out and replaced with medium containing different concentrations of test compounds, 1.25% Avicel microcrystalline cellulose, and 1 μg/mL TPCK-treated trypsin. At 48 h p.i., cell monolayers were fixed with 4% formaldehyde solution and stained for microplaque assay.

### Virus Yield Reduction Assay (VYRA)

*Vero cells* were seeded at a density of 2.5 × 10^5^ cells per well in 24-well plates and incubated overnight at 37 °C. The cells were then infected with the NDV La Sota strain at a multiplicity of infection (MOI) of 0.001 in MEM plus 1 μg/mL TPCK-treated trypsin for 1 h at 37 °C. After 1 h, media were replaced with fresh MEM containing 1 μg/mL TPCK-treated trypsin and compounds at various concentrations. The culture supernatants were collected at 24 h p.i. and viral progeny was titrated by the FFA in fresh *Vero cells*.

### Statistical Analysis

Statistical analysis was carried out using GraphPad Prism version 8.0 (GraphPad Software, San Diego, CA). Data are presented as the mean ± standard deviation (SD) of at least two experiments in duplicate. The IC_50_ and CC_50_ values for the different assays were determined by nonlinear regression curve fitting (inhibitor versus normalized response with variable slope equation) using GraphPad Prism 8.0 Software.

## Supporting Information Available

IC_50_ values for all of the final compounds; multiple sequence alignment; supplementary methods and references; and copies of NMR spectra for the new compounds (PDF)

## Abbreviations

hPIVs: human parainfluenza viruses
NDV: Newcastle disease virus
HN: hemagglutinin-neuraminidase
IC_50_: half-maximal inhibitory concentration
DANA or Neu5Ac2en: *N*-acetyl-2,3-dehydro-2-deoxyneuraminic acid
N: neuraminidase
4-MUNeu5Ac: 2′-(4-methylumbelliferyl)-α-d-*N*-acetylneuraminic acid
NI: neuraminidase inhibition
FANA: *N*-trifluoroacetyl-2,3-dehydro-2-deoxyneuraminic acid
PRA: plaque reduction assay
CC_50_: half-maximal cytotoxicity concentration
SI: selectivity index
VYRA: viral yield reduction assay
MOI: multiplicity of infection
PFU: plaque-forming unit
