# Supplementary material for: Design, Synthesis, and Antiviral Evaluation of Sialic Acid Derivatives as Inhibitors of Newcastle Disease Virus Hemagglutinin-Neuraminidase: A Translational Study on Human Parainfluenza Viruses
Source: ACS Infect Dis. 2023 Feb 27;9(3):617–30. doi: 10.1021/acsinfecdis.2c00576 (PMC10012260; doi:10.1021/acsinfecdis.2c00576)
Supplement: Supplementary file 1 — id2c00576_si_001.pdf [file id2c00576_si_001.pdf]

Supporting Information

Design, Synthesis, and Antiviral Evaluation of Sialic Acid Derivatives as Inhibitors of Newcastle Disease Virus Hemagglutinin-Neuraminidase: A Translational Study on Human Parainfluenza Viruses

Paola Rota,[*,†,°] Paolo La Rocca,[#,°] Francesco Bonfante,[*,§] Matteo Pagliari,[§] Marco Piccoli,[ǁ,°] Federica Cirillo,[ǁ,°] Andrea Ghiroldi,[ǁ,°] Valentina Franco,[+,©] Carlo Pappone,[°,&,‡] Pietro Allevi[†] and Luigi Anastasia[*ǁ,‡,°]

[†] Department of Biomedical, Surgical and Dental Sciences, Università degli Studi di Milano, 20133 Milan, Italy. E-mail: *paola.rota@unimi.it

[°] Institute for Molecular and Translational Cardiology (IMTC), 20097 San Donato Milanese, Milan, Italy

[#] Department of Biomedical Sciences for Health, Università degli Studi di Milano, 20133 Milan, Italy.

[§] Division of Comparative Biomedical Sciences, Istituto Zooprofilattico Sperimentale delle Venezie, 35020 Legnaro, Italy. E-mail: *FBonfante@izsvenezie.it

[ǁ] Laboratory of Stem Cells for Tissue Engineering, IRCCS Policlinico San Donato, 20097 San Donato Milanese, Milan, Italy.

[+] Division of Clinical and Experimental Pharmacology, Department of Internal Medicine and Therapeutics, University of Pavia, 27100 Pavia, Italy.

[©] IRCCS, Mondino Foundation, 27100 Pavia, Italy.

[&] Arrhythmology Department, IRCCS Policlinico San Donato, Piazza Malan 2, 20097 San Donato Milanese, Milan, Italy.

[‡] Faculty of Medicine, University of Vita-Salute San Raffaele, 20132 Milan, Italy E-mail: *anastasia.luigi@hsr.it

Contents	Page Number
Table S1: IC_{50} values for compounds **1**, **2**, **4**, **5**, **9**-**14**, **19**-**24**	S2
Figure S1: Multiple sequence alignment	S3
Supplementary methods	S4
Bibliography	S4
^{1}H and ^{13}C NMR of the protected compounds **17**, **18**, **25**-**30** and **32**	S5-S13
^{1}H and ^{13}C NMR of the free final compounds **13**, **14**, **19**-**24** and **31**	S14-S22

Table S1 IC_{50} values for the different inedited or known DANA derivatives tested on NDV-HN of La Sota Clone 30, La Sota 40/14 or the velogenic Egyptian strain. Each value represents the mean of three independent experiments carried out in triplicate.

Neuraminidase Inhibition Assay			
**Compound**	**IC_{50} (μM)**		
	**La Sota Clone 30**	**La Sota 40/14**	**Egyptian**
**DANA 1**	15 ± 1	7.0 ± 1.0	6.0 ± 1.0
**FANA 5**	2.4 ± 0.3	1.4 ± 0.2	0.72 ± 0.09
**BCX-2798 4**	0.32 ± 0.04	0.30 ± 0.06	0.11 ± 0.02
**Zanamivir 2**	23 ± 6	34 ± 4	70 ± 30
**13**	13 ± 2	N.D.	N.D.
**14**	6.3 ± 0.6	N.D.	N.D.
**20**	11 ± 1	N.D.	N.D.
**19**	0.60 ± 0.08	N.D.	N.D.
**12**	0.19 ± 0.01	0.34 ± 0.03	0.28 ± 0.01
**24**	0.20 ± 0.02	0.11 ± 0.01	0.13 ± 0.01
**21**	1.2 ± 0.1	N.D.	N.D.
**22**	0.85 ± 0.11	N.D.	N.D.
**23**	0.06 ± 0.01	0.10 ± 0.03	0.17 ± 0.03
**11**	0.18 ± 0.01	0.20 ± 0.01	0.17 ± 0.01
**9**	0.17 ± 0.06	0.05 ± 0.01	0.03 ± 0.01
**10**	0.21 ± 0.04	0.07 ± 0.03	0.03 ± 0.01

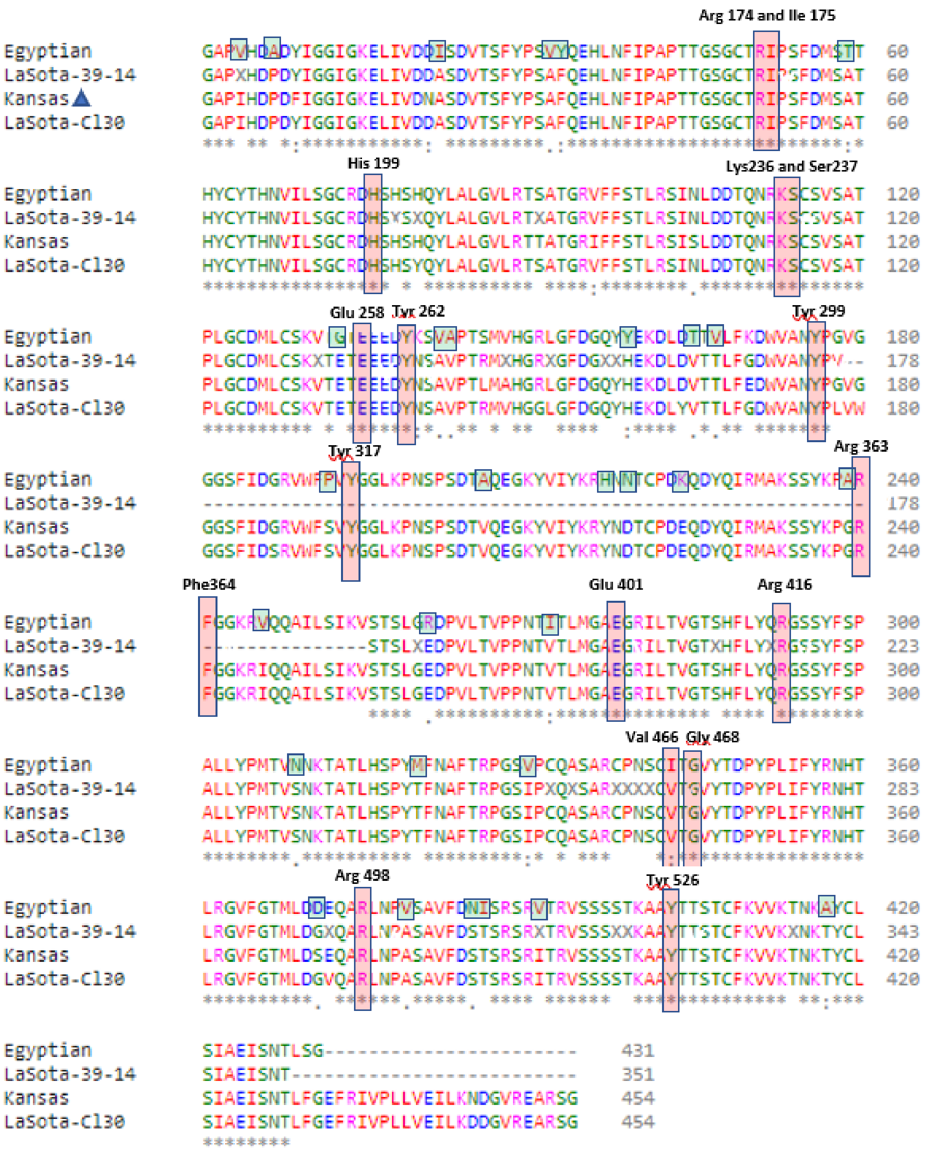

Figure S1 Multiple sequence alignment of HN head domains from different NDV strains performed with Clustal Omega software (see Supplementary Methods). The aminoacids are coloured in relation to their chemical R-group nature (red: hydrophobic, blue: acidic, purple: basic and green: polar). Asterisks indicate identical aminoacids, dots indicate similar aminoacids, empty space indicates not conserved aminoacids. The catalytic site aminoacids are squared in red. Aminoacids not conserved in velogenic Egyptian strain (respect to the two La Sota strains) are squared in green.

SUPPLEMENTARY METHODS

HN gene sequencing. The HN nucleotidic sequences belonging to La Sota 39/14 and the velogenic Egyptian strain APMV-1/chicken/Egypt/13VIR-5009-2/2013 were obtained from Istituto Zooprofilattico Sperimentale delle Venezie. The sequence deriving from the La Sota 39/14 strain is incomplete due to problems during the sequencing process.

Protein sequence collection. HN head domain aminoacidic sequence (from residue 124 to 570) of NDV Kansas strain (used as model in docking simulation studies[1]) was directly derived from the crystal structure (PDB 1e8v).[2] HN head domain sequence of La Sota Clone 30 was obtained from GenBank.[3] The aminoacidic sequences of the HN head domain of La Sota 39/14 and the velogenic Egyptian strain APMV-1/chicken/Egypt/13VIR-5009-2/2013 were obtained by translating the nucleotidic sequence corresponding to the whole protein using the ExPASy Translate Tool (https://web.expasy.org/translate/)[4] and, successively selecting only the head domain aminoacids.

Protein alignments. Protein alignments of the four proteic sequences were performed using Clustal Omega software (https://www.ebi.ac.uk/Tools/msa/clustalo/)[5] maintaining default settings.

REFERENCES

1. Rota, P., La Rocca, P., Piccoli, M., Montefiori, M., Cirillo, F., Olsen, L., Orioli, M., Allevi, P., and Anastasia, L. (2018) Potent inhibitors against Newcastle disease virus hemagglutinin-neuraminidase, *ChemMedChem 13(3)*, 236-240.
2. Crennell, S., Takimoto, T., Portner, A., and Taylor, G. (2000) Crystal structure of the multifunctional paramyxovirus hemagglutinin-neuraminidase, *Nat. Struct. Biol. 7(11)*, 1068-1074.
3. Sagrera, A., Cobaleda, C., Gonzalez De Buitrago, J. M., Garcia-Sastre, A., and Villar, E. (2001) Membrane glycoproteins of Newcastle disease virus: Nucleotide sequence of the hemagglutinin-neuraminidase cloned gene and structure/function relationship of predicted amino acid sequence, *Glycoconjugate J. 18(4)*, 283-289.
4. Gasteiger, E., Gattiker, A., Hoogland, C., Ivanyi, I., Appel, R. D., and Bairoch, A. (2003) ExPASy: the proteomics server for in-depth protein knowledge and analysis, *Nucleic Acids Res. 31(13)*, 3784-3788.
5. Sievers, F., Wilm, A., Dineen, D., Gibson, T. J., Karplus, K., Li, W. Z., Lopez, R., McWilliam, H., Remmert, M., Soding, J., Thompson, J. D., and Higgins, D. G. (2011) Fast, scalable generation of high-quality protein multiple sequence alignments using Clustal Omega, *Mol. Syst. Biol. 7*, 539.

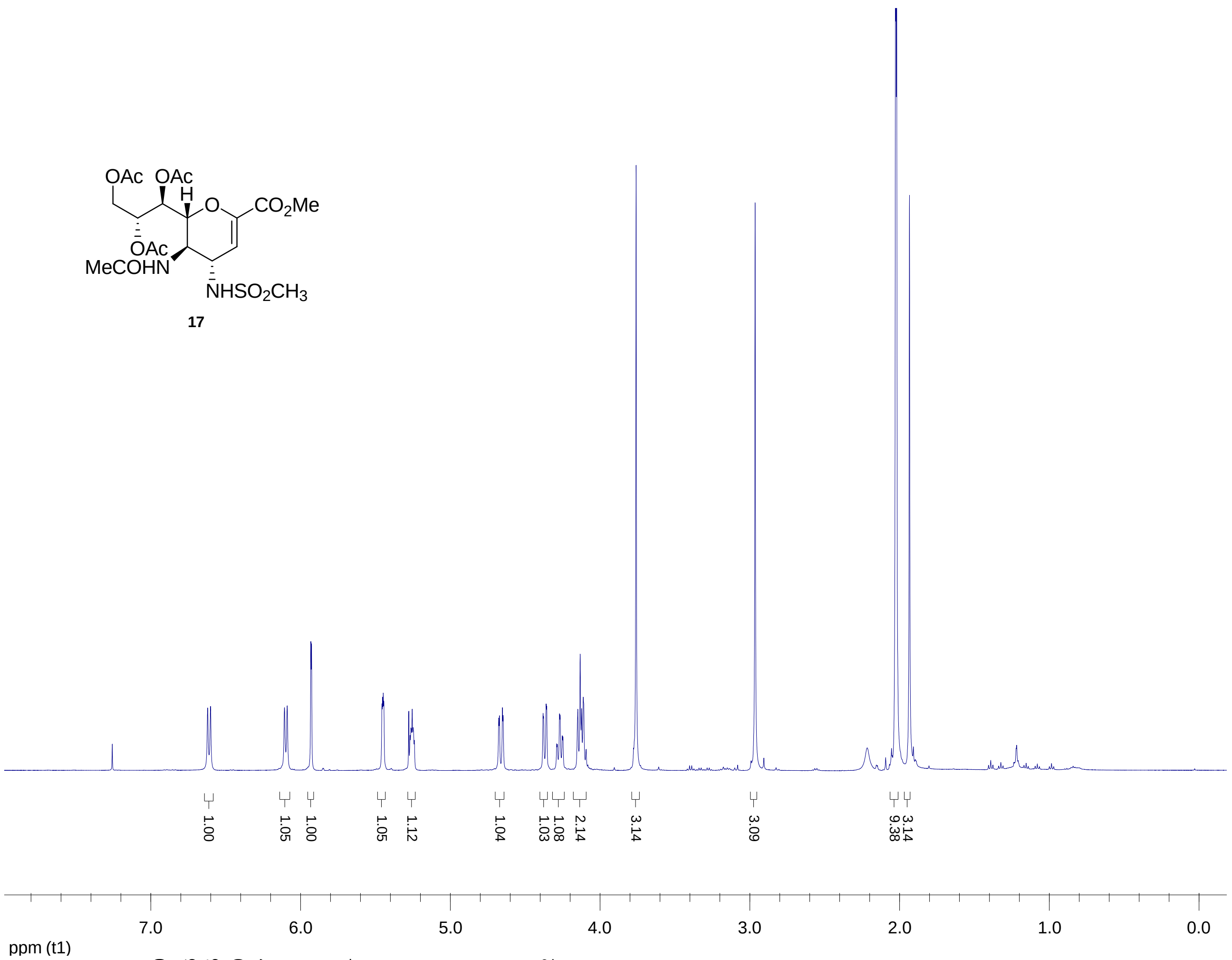
OAc OAc
H
O
CO2Me
OAc
MeCOHN
NHSO2CH3
17
1.00
1.05
1.00
1.05
1.12
1.04
1.03
1.08
2.14
3.14
3.09
9.38
3.14
7.0
6.0
5.0
4.0
3.0
2.0
1.0
0.0
ppm (t1)

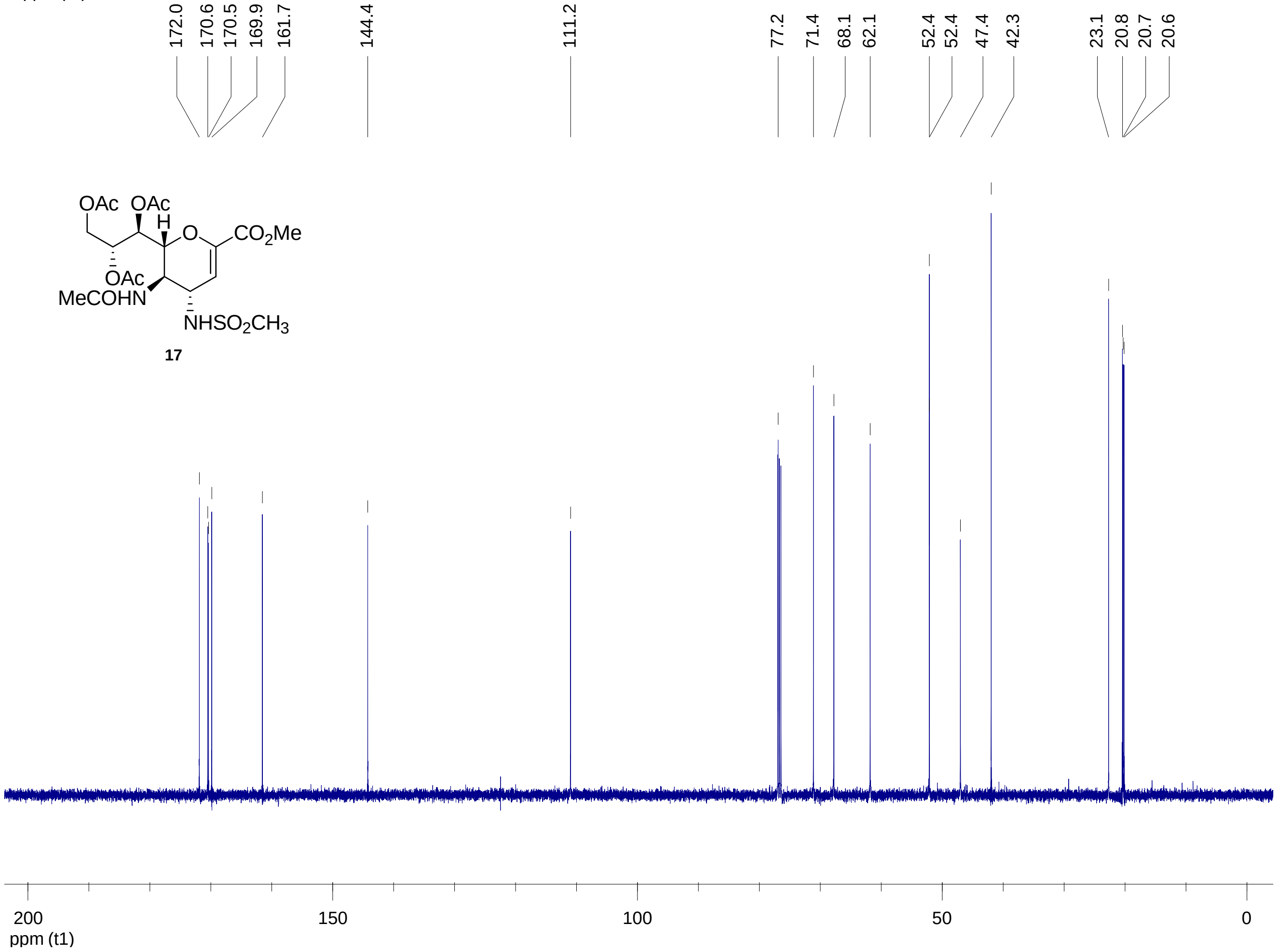
172.0
170.6
170.5
169.9
161.7
144.4
111.2
77.2
71.4
68.1
62.1
52.4
52.4
47.4
42.3
23.1
20.8
20.7
20.6
OAc OAc
H
O
CO2Me
OAc
MeCOHN
NHSO2CH3
17
200
150
100
50
0
ppm (t1)

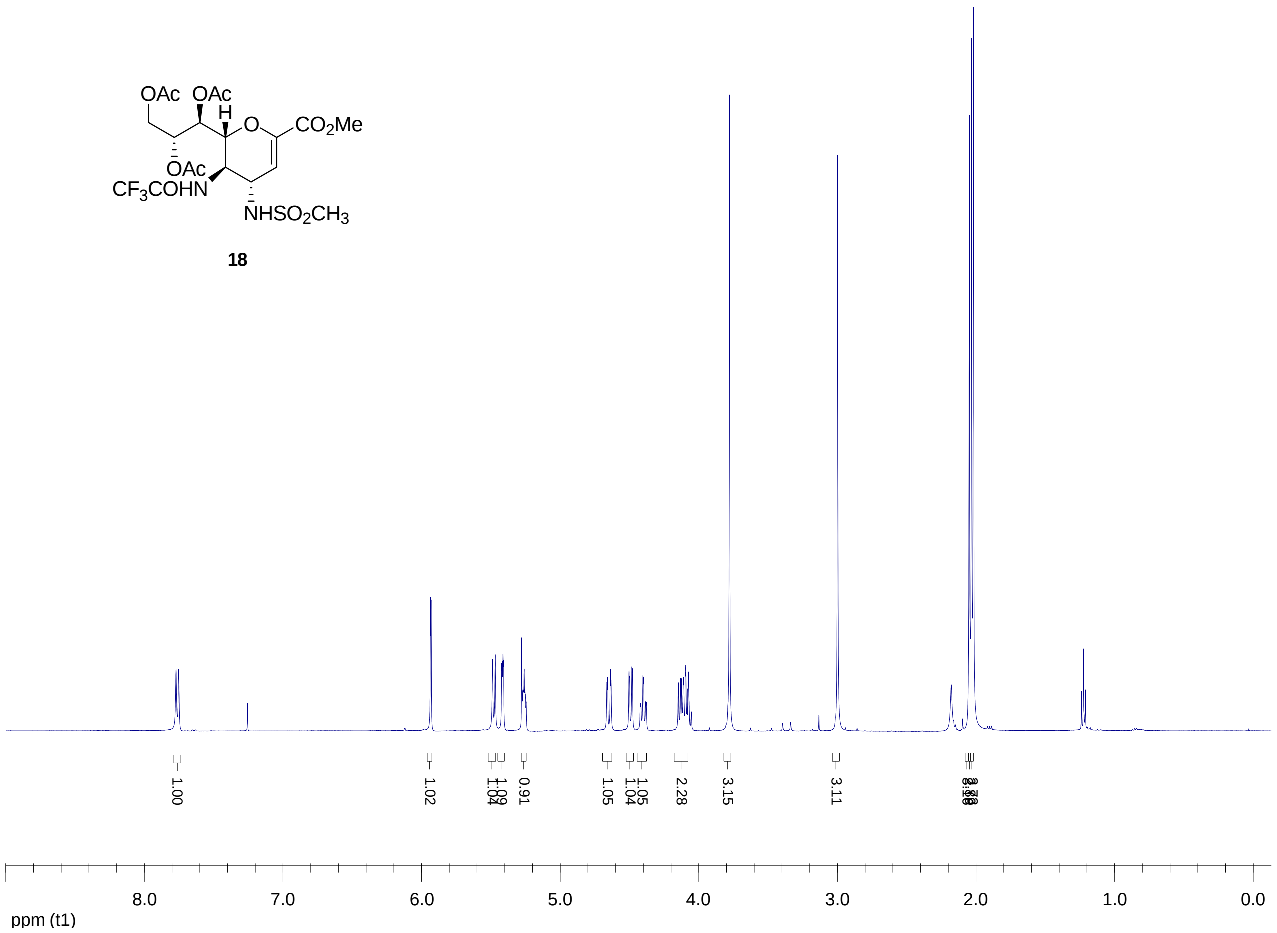
OAc OAc
H
O
CO2Me
OAc
CF3COHN
NHSO2CH3
18
1.00
1.02
1.04
1.09
0.91
1.05
1.04
1.05
2.28
3.15
3.11
8.0
7.0
6.0
5.0
4.0
3.0
2.0
1.0
0.0
ppm (t1)

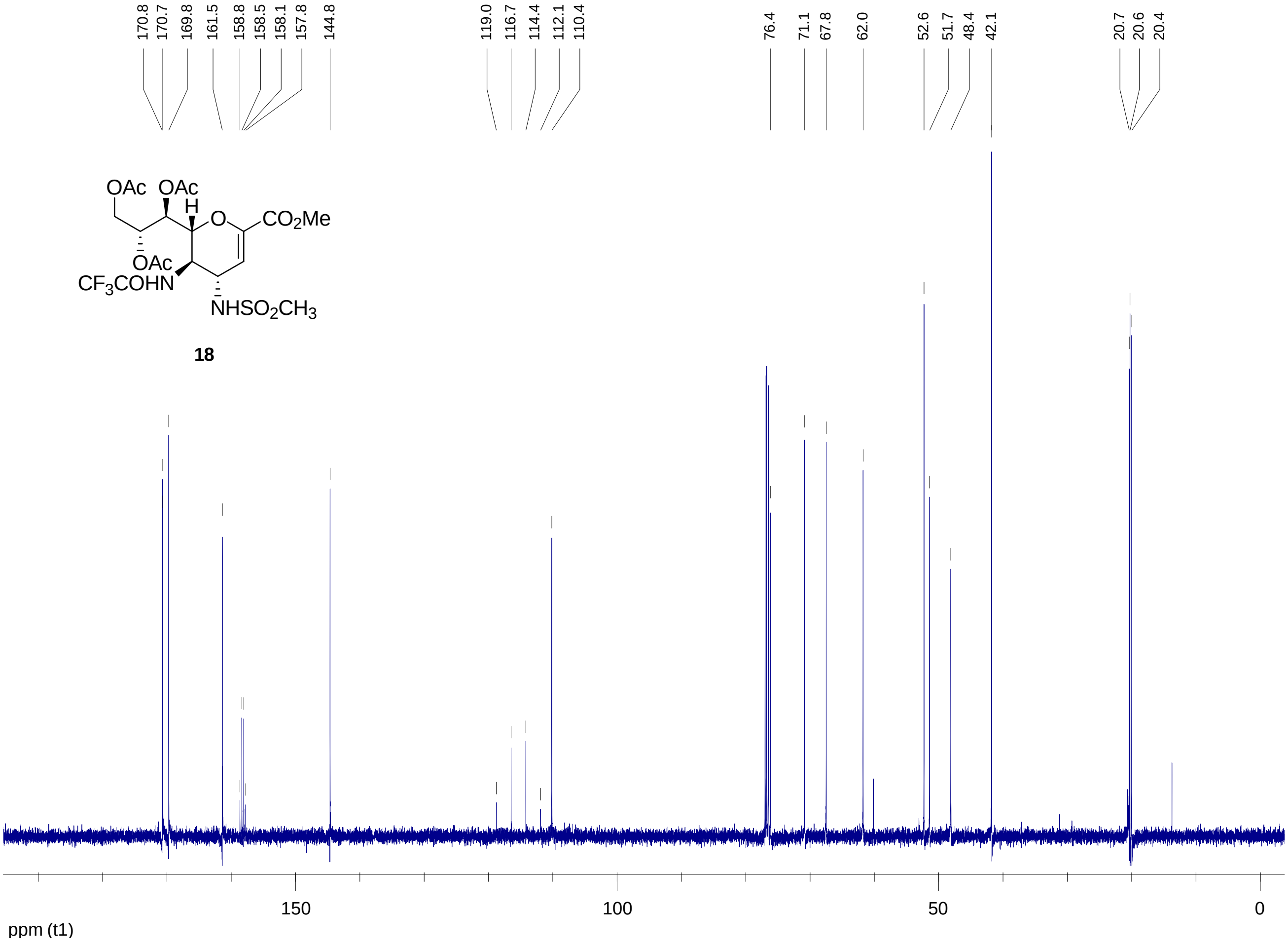
170.8
170.7
169.8
161.5
158.8
158.5
158.1
157.8
144.8
119.0
116.7
114.4
112.1
110.4
76.4
71.1
67.8
62.0
52.6
51.7
48.4
42.1
20.7
20.6
20.4
OAc OAc
H
O
CO2Me
OAc
CF3COHN
NHSO2CH3
18
150
100
50
0
ppm (t1)

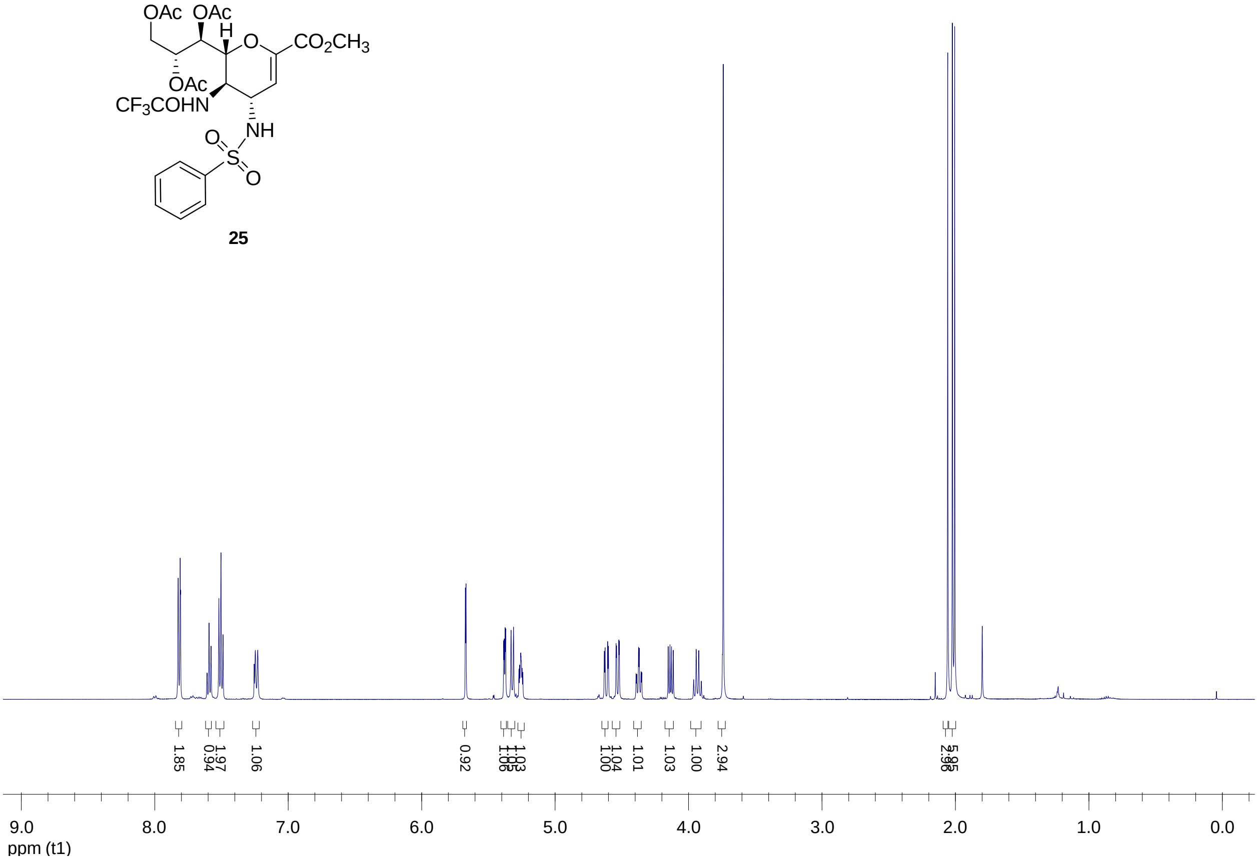
OAc
OAc
H
O
CO_2CH_3
OAc
CF_3COHN
NH
O
S
O
25
1.85
0.94
1.97
1.06
0.92
1.03
1.04
1.06
1.01
1.03
1.00
2.94
9.0
8.0
7.0
6.0
5.0
4.0
3.0
2.0
1.0
0.0
ppm (t1)

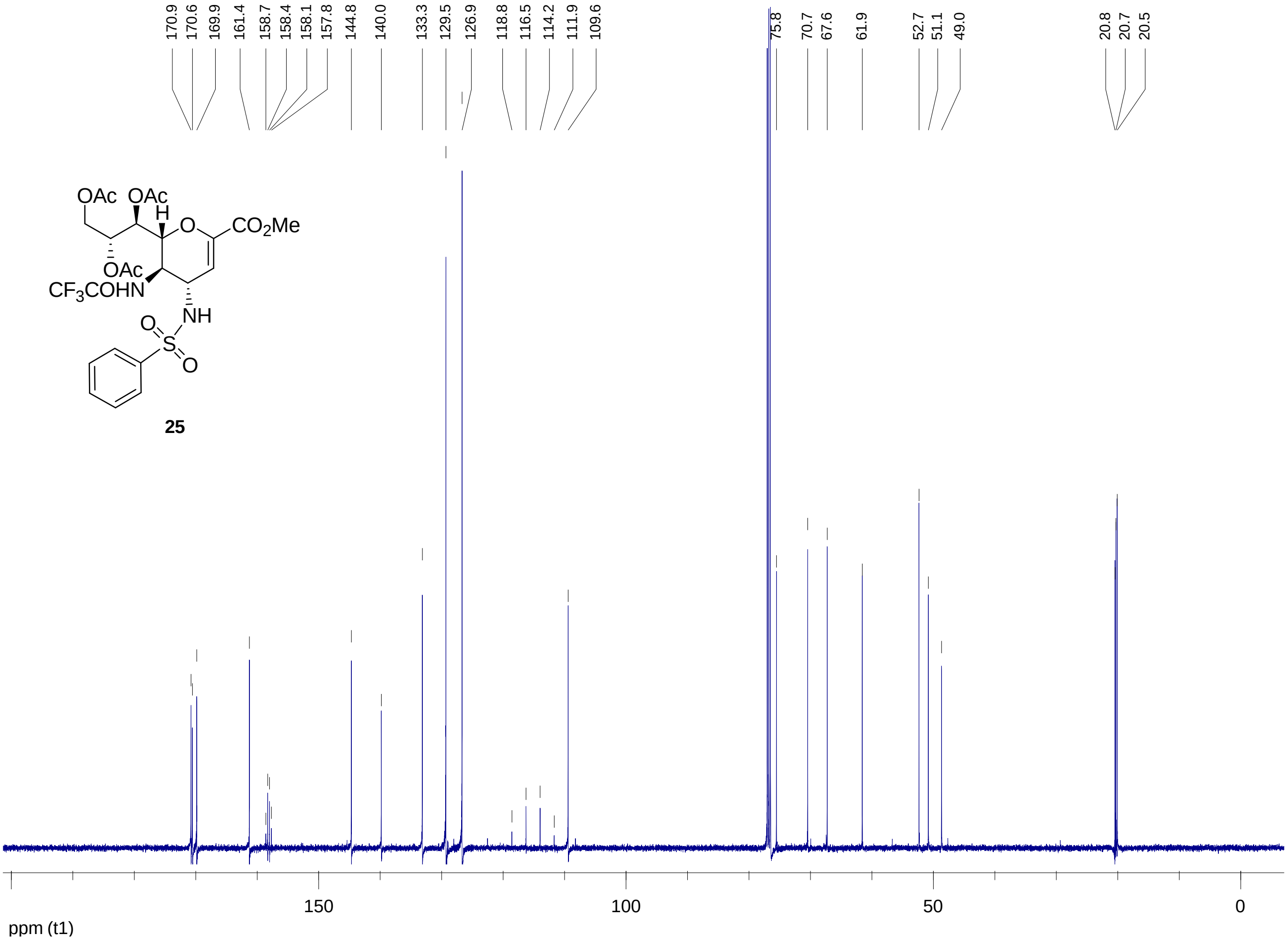
170.9
170.6
169.9
161.4
158.7
158.4
158.1
157.8
144.8
140.0
133.3
129.5
126.9
118.8
116.5
114.2
111.9
109.6
75.8
70.7
67.6
61.9
52.7
51.1
49.0
20.8
20.7
20.5
OAc
OAc
H
O
CO_2Me
OAc
CF_3COHN
NH
O
S
O
25
150
100
50
0
ppm (t1)

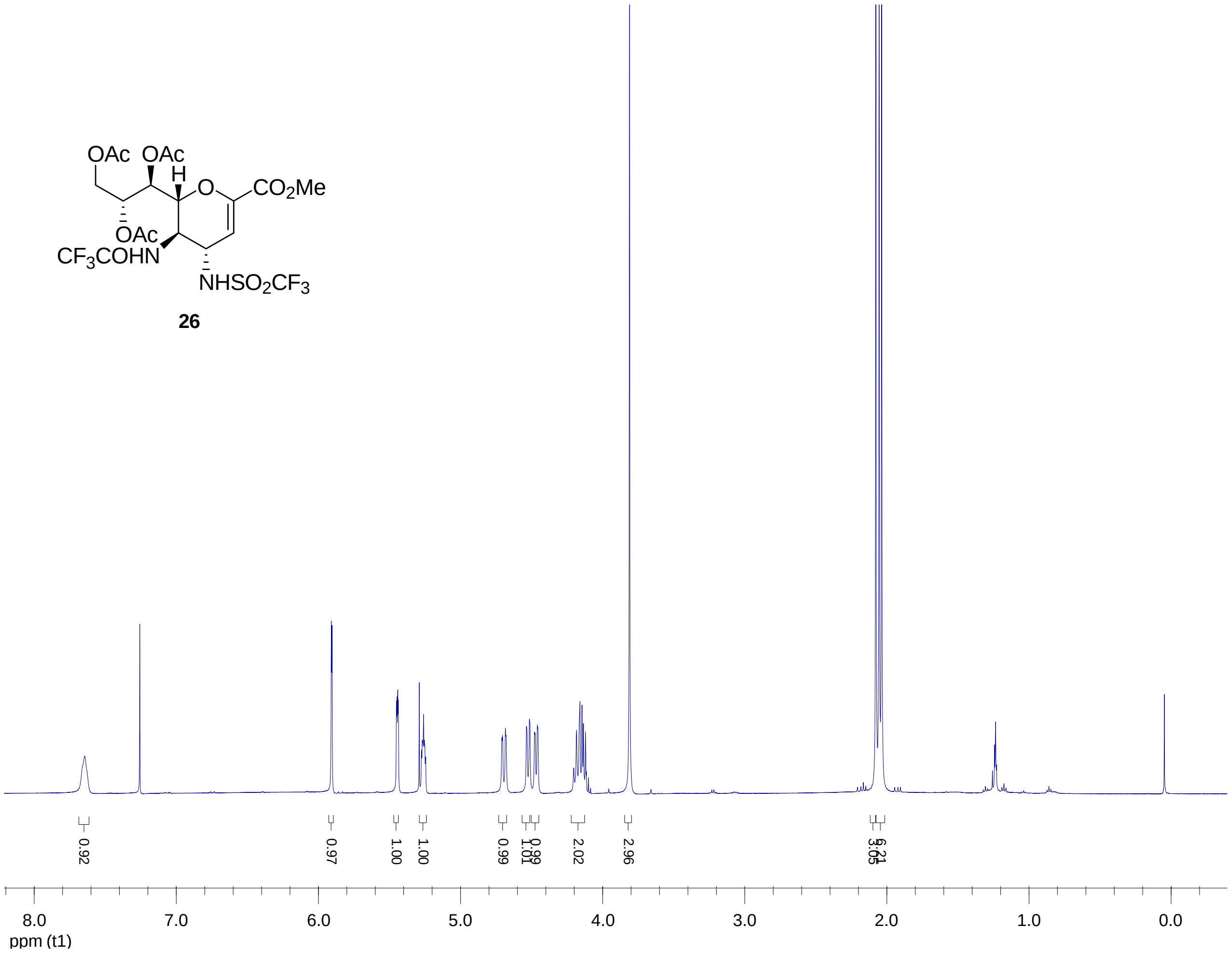

26
0.92
0.97
1.00
1.00
0.99
1.01
0.99
2.02
2.96
3.05
6.21
8.0
7.0
6.0
5.0
4.0
3.0
2.0
1.0
0.0
ppm (t1)

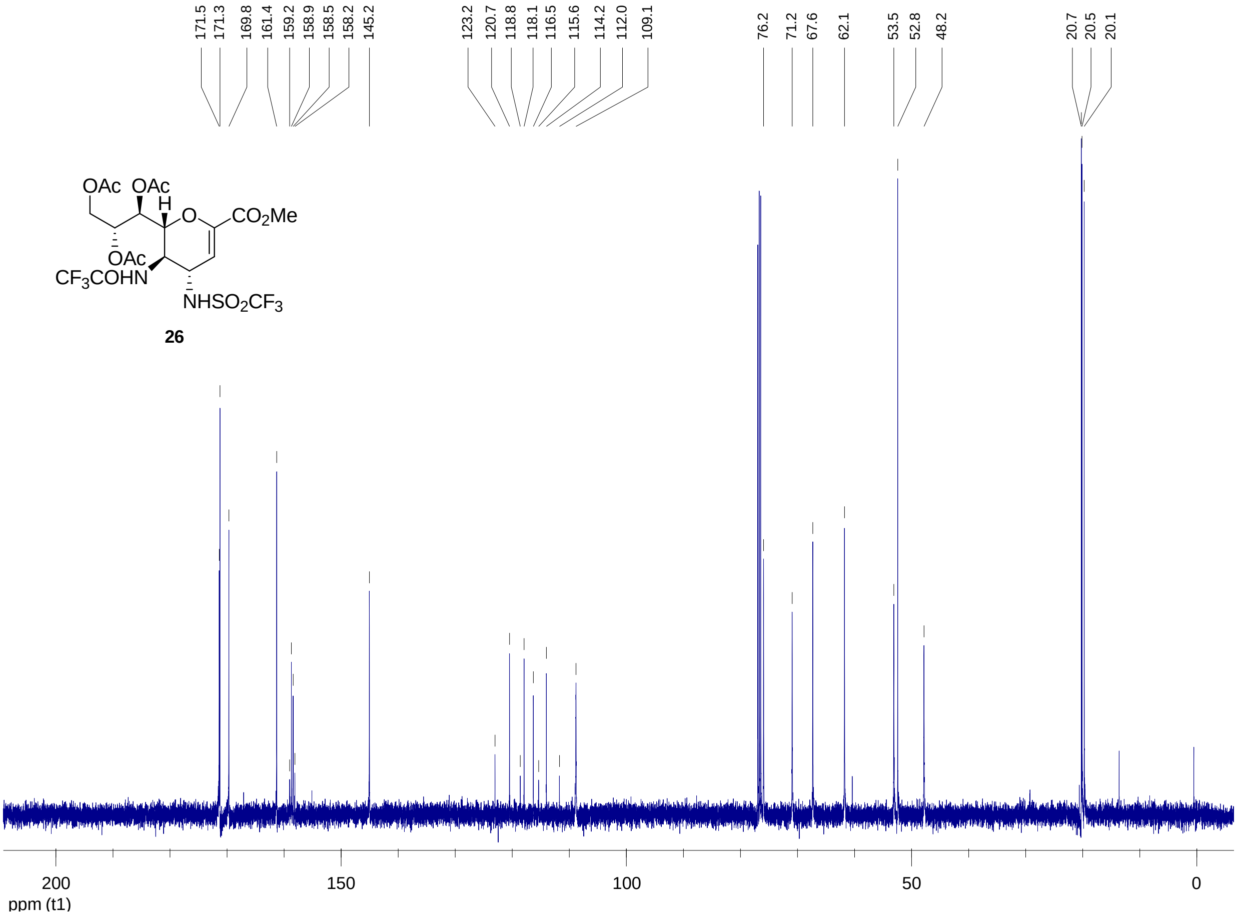

171.5
171.3
169.8
161.4
159.2
158.9
158.5
158.2
145.2
123.2
120.7
118.8
118.1
116.5
115.6
114.2
112.0
109.1
76.2
71.2
67.6
62.1
53.5
52.8
48.2
20.7
20.5
20.1
26
200
150
100
50
0
ppm (t1)

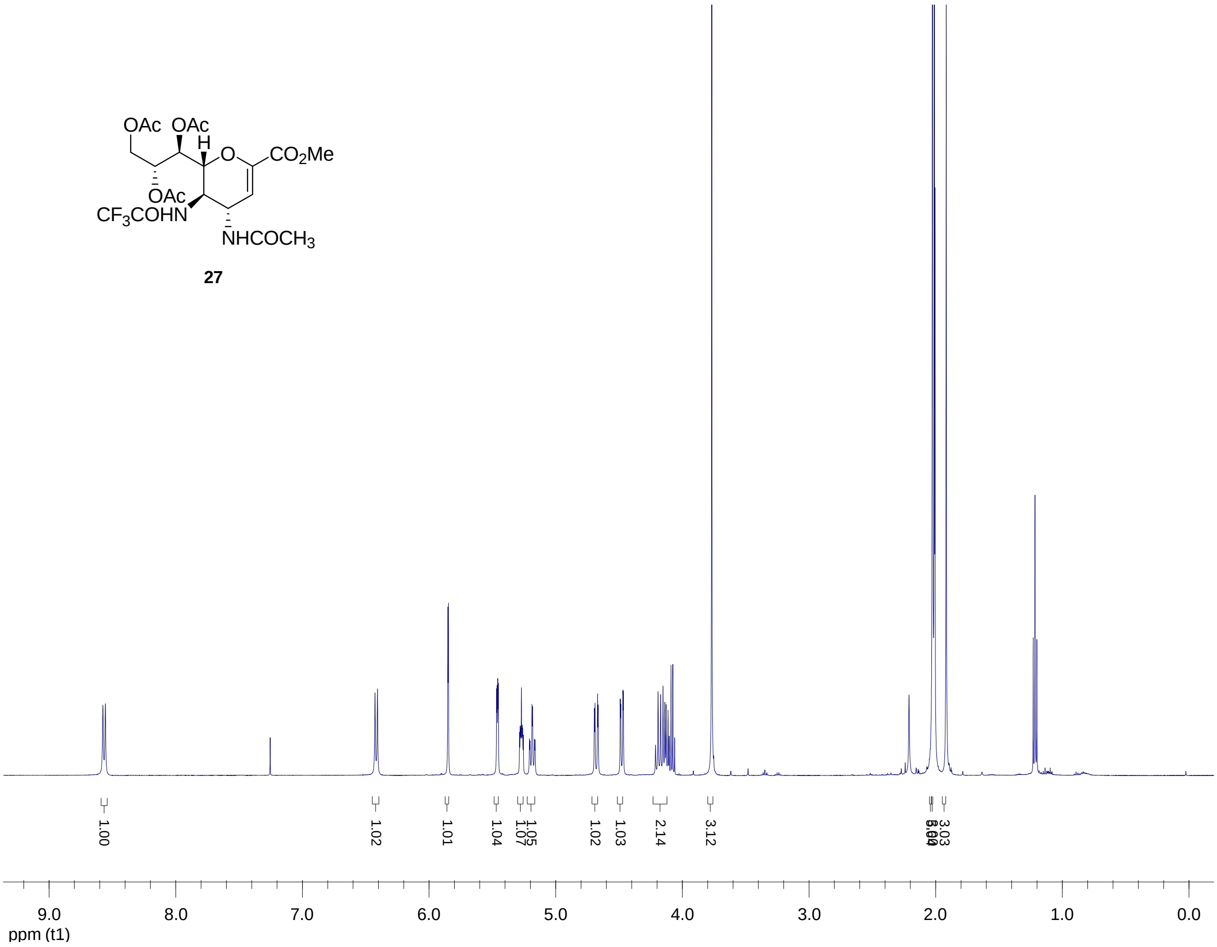
OAc OAc
H
O
CO2Me
OAc
CF3COHN
NHCOCH3
27
1.00
1.02
1.01
1.04
1.07
1.05
1.02
1.03
2.14
3.12
3.03
9.0
8.0
7.0
6.0
5.0
4.0
3.0
2.0
1.0
0.0
ppm (t1)

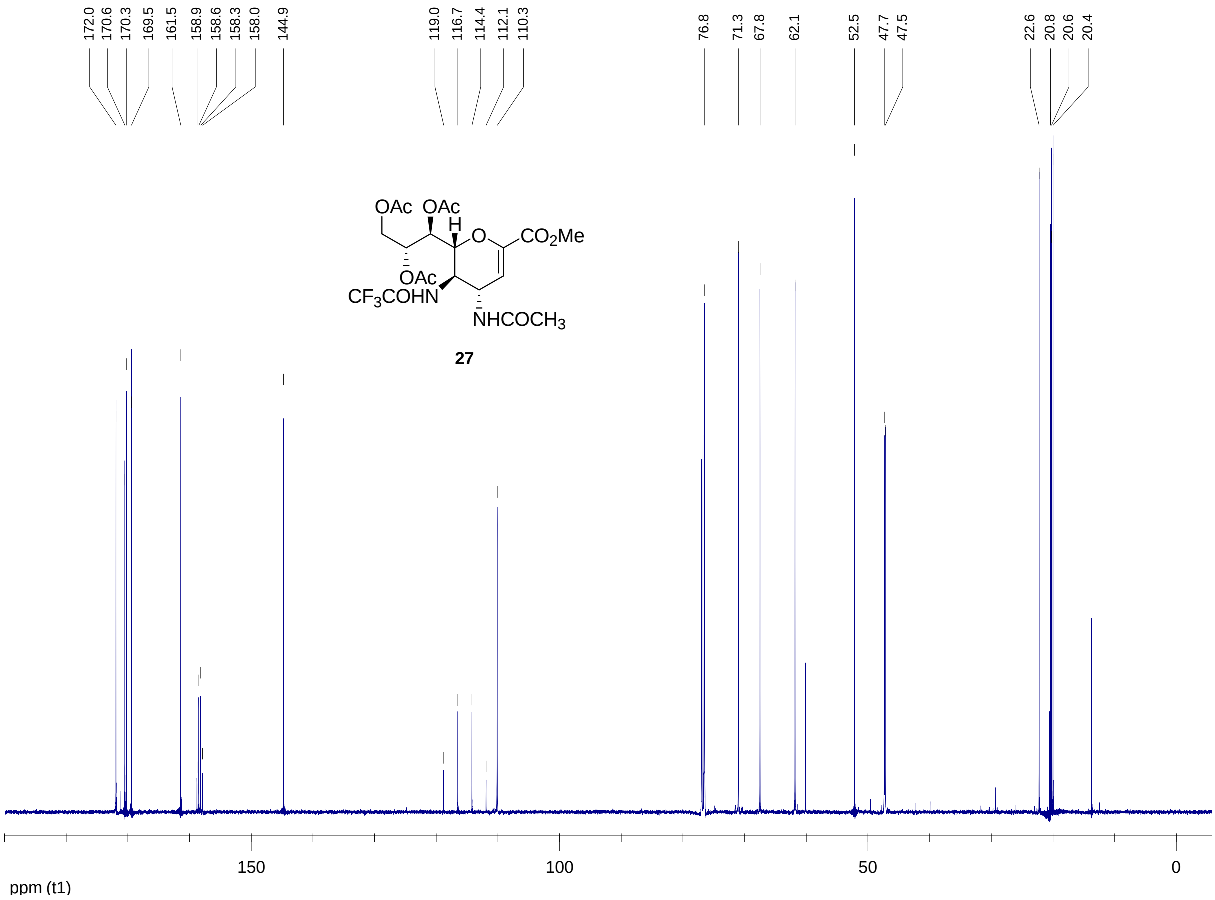
172.0
170.6
170.3
169.5
161.5
158.9
158.6
158.3
158.0
144.9
119.0
116.7
114.4
112.1
110.3
76.8
71.3
67.8
62.1
52.5
47.7
47.5
22.6
20.8
20.6
20.4
OAc OAc
H
O
CO2Me
OAc
CF3COHN
NHCOCH3
27
150
100
50
0
ppm (t1)

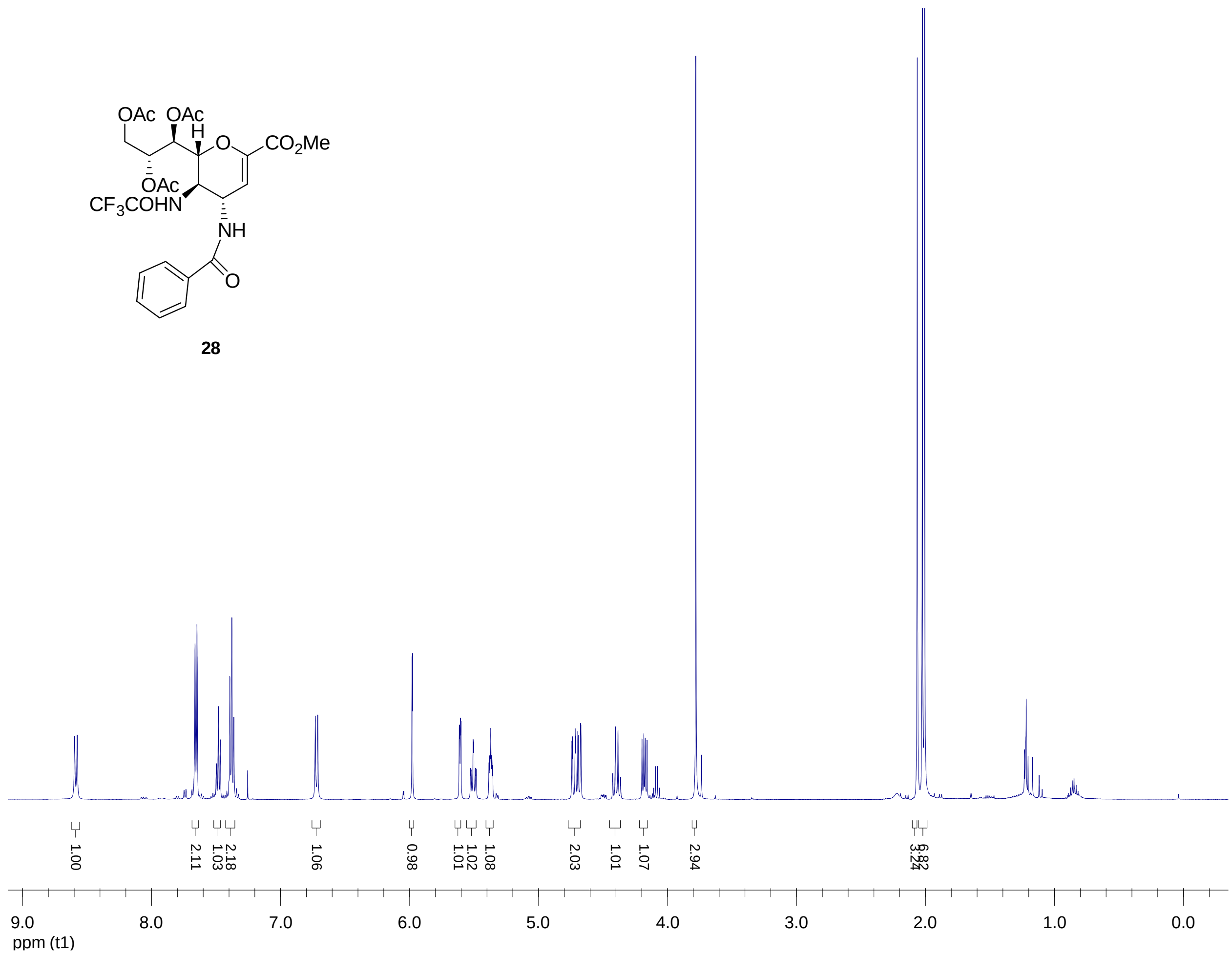
OAc OAc
H
O
CO2Me
OAc
CF3COHN
NH
O
28
1.00
2.11
1.03
2.18
1.06
0.98
1.01
1.02
1.08
2.03
1.01
1.07
2.94
9.0 8.0 7.0 6.0 5.0 4.0 3.0 2.0 1.0 0.0
ppm (t1)

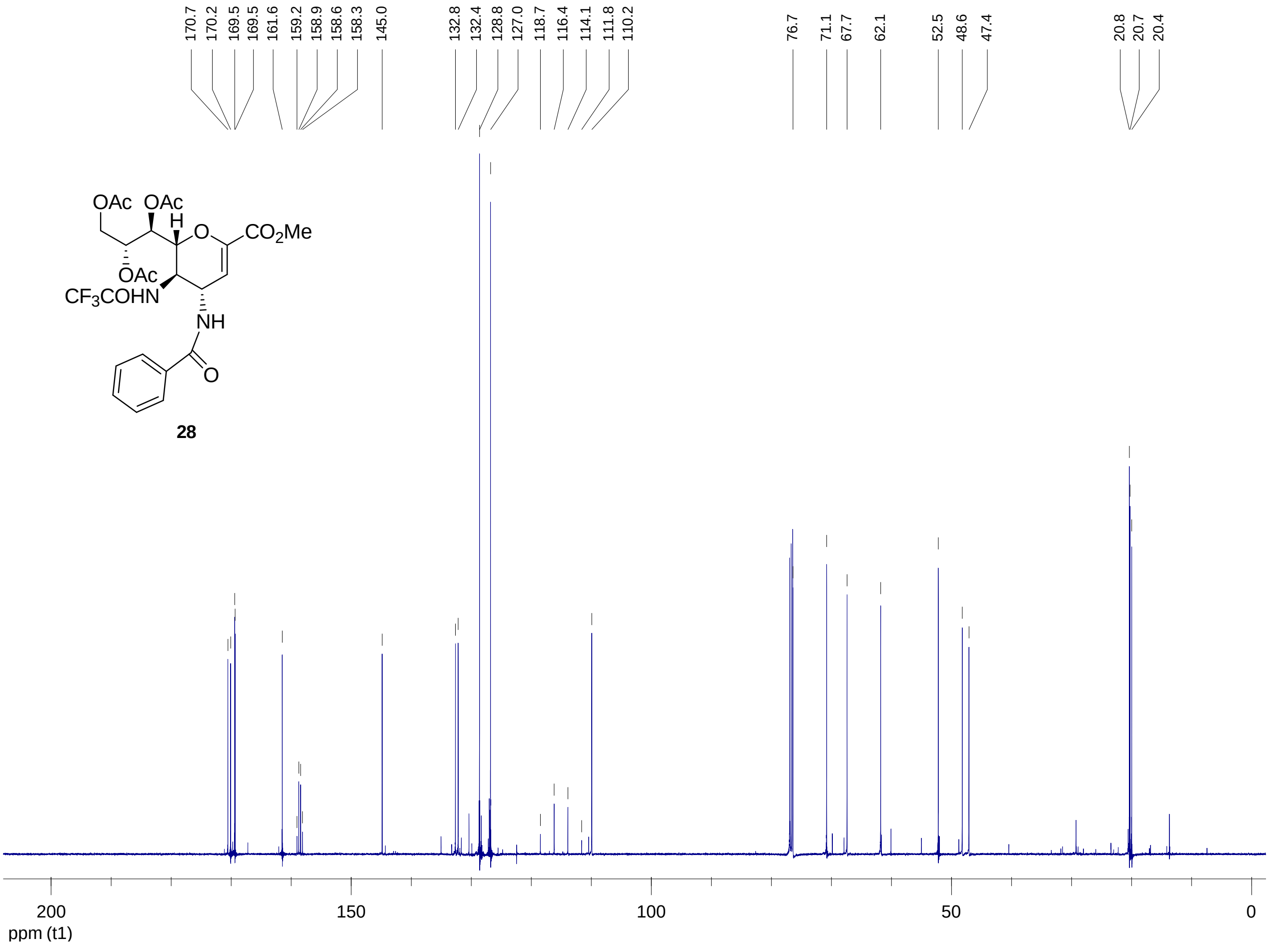
170.7
170.2
169.5
169.5
161.6
159.2
158.9
158.6
158.3
145.0
132.8
132.4
128.8
127.0
118.7
116.4
114.1
111.8
110.2
76.7
71.1
67.7
62.1
52.5
48.6
47.4
20.8
20.7
20.4
OAc OAc
H
O
CO2Me
OAc
CF3COHN
NH
O
28
200 150 100 50 0
ppm (t1)

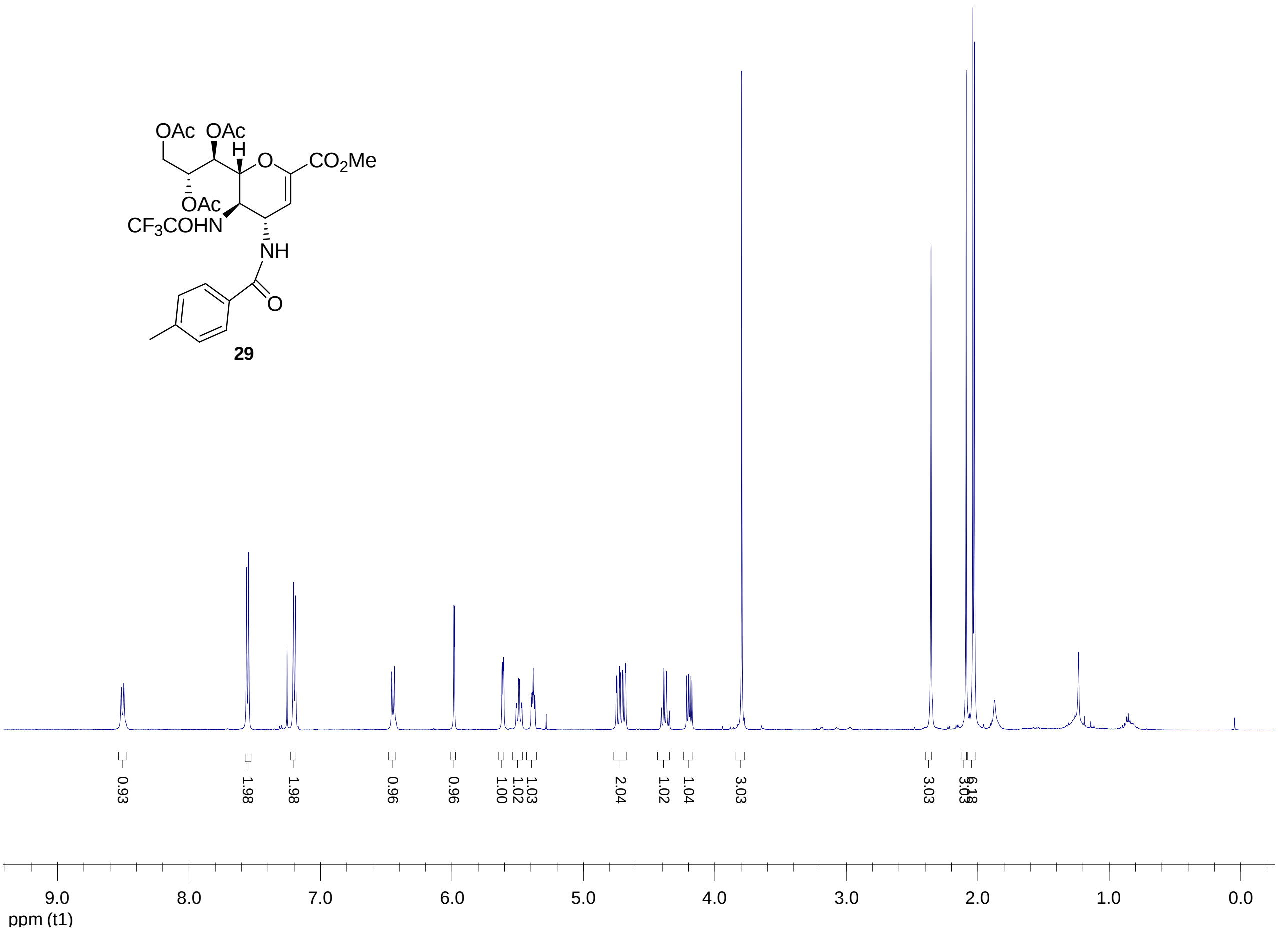
OAc OAc
H
O
CO2Me
OAc
CF3COHN
NH
O
29
0.93
1.98
1.98
0.96
0.96
1.00
1.02
1.03
2.04
1.02
1.04
3.03
3.03
3.03
6.18
9.0
8.0
7.0
6.0
5.0
4.0
3.0
2.0
1.0
0.0
ppm (t1)

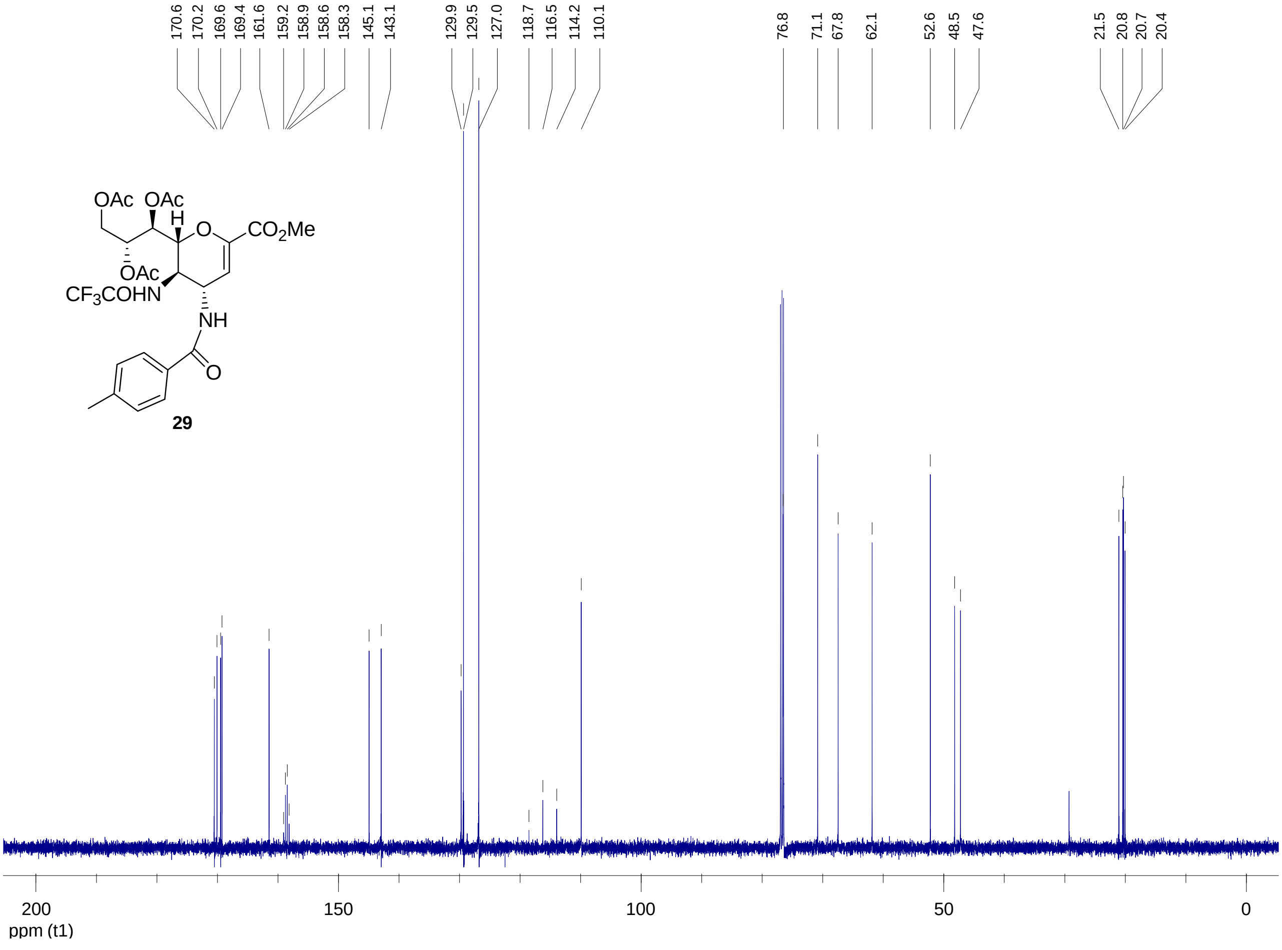
170.6
170.2
169.6
169.4
161.6
159.2
158.9
158.6
158.3
145.1
143.1
129.9
129.5
127.0
118.7
116.5
114.2
110.1
76.8
71.1
67.8
62.1
52.6
48.5
47.6
21.5
20.8
20.7
20.4
OAc OAc
H
O
CO2Me
OAc
CF3COHN
NH
O
29
200
150
100
50
0
ppm (t1)

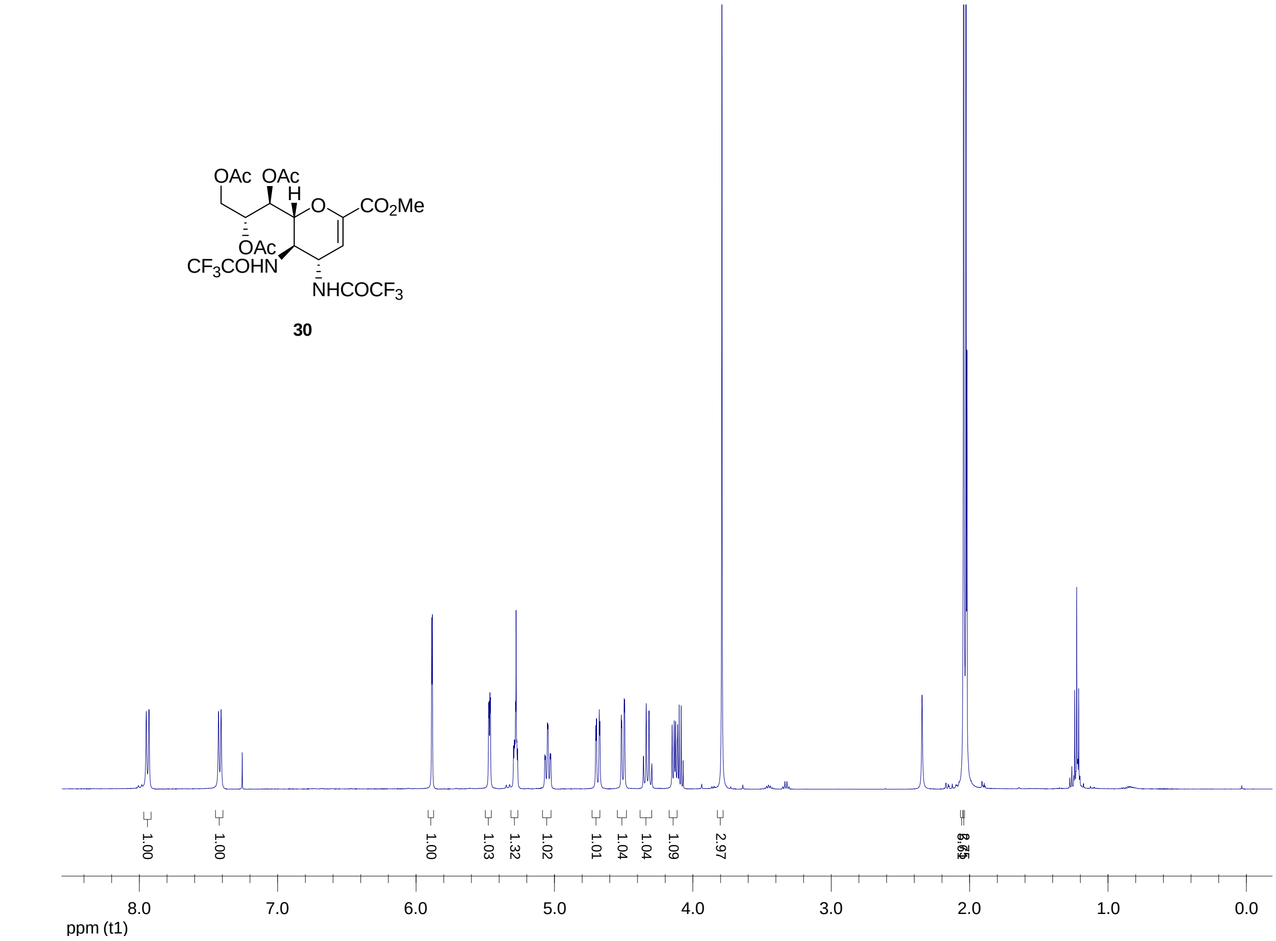

OAc OAc
H
O
CO2Me
OAc
CF3COHN
NHCOCF3
30
1.00
1.00
1.00
1.03
1.32
1.02
1.01
1.04
1.04
1.09
2.97
8.0
7.0
6.0
5.0
4.0
3.0
2.0
1.0
0.0
ppm (t1)

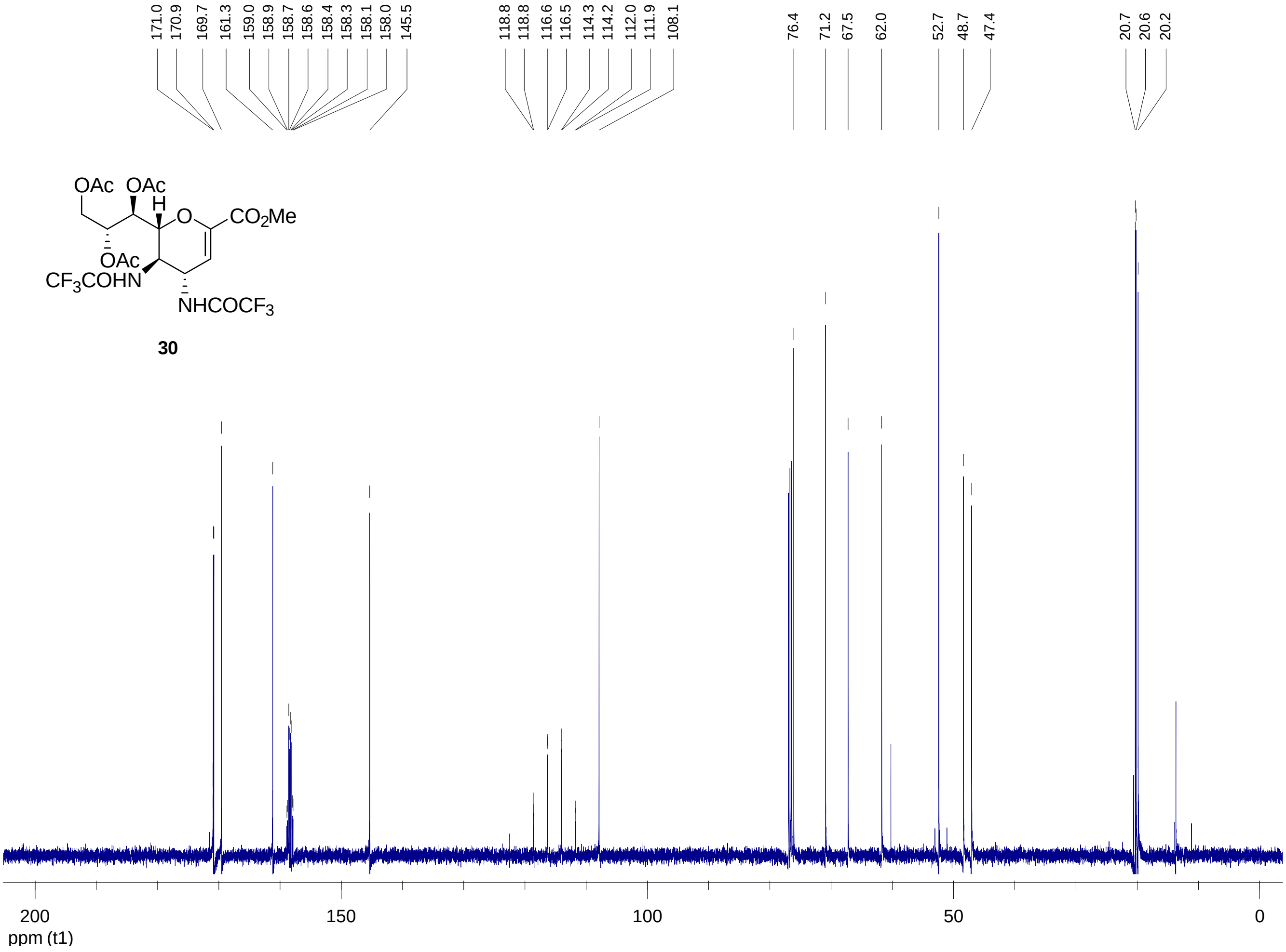

171.0
170.9
169.7
161.3
159.0
158.9
158.7
158.6
158.4
158.3
158.1
158.0
145.5
118.8
118.8
116.6
116.5
114.3
114.2
112.0
111.9
108.1
76.4
71.2
67.5
62.0
52.7
48.7
47.4
20.7
20.6
20.2
OAc OAc
H
O
CO2Me
OAc
CF3COHN
NHCOCF3
30
200
150
100
50
0
ppm (t1)

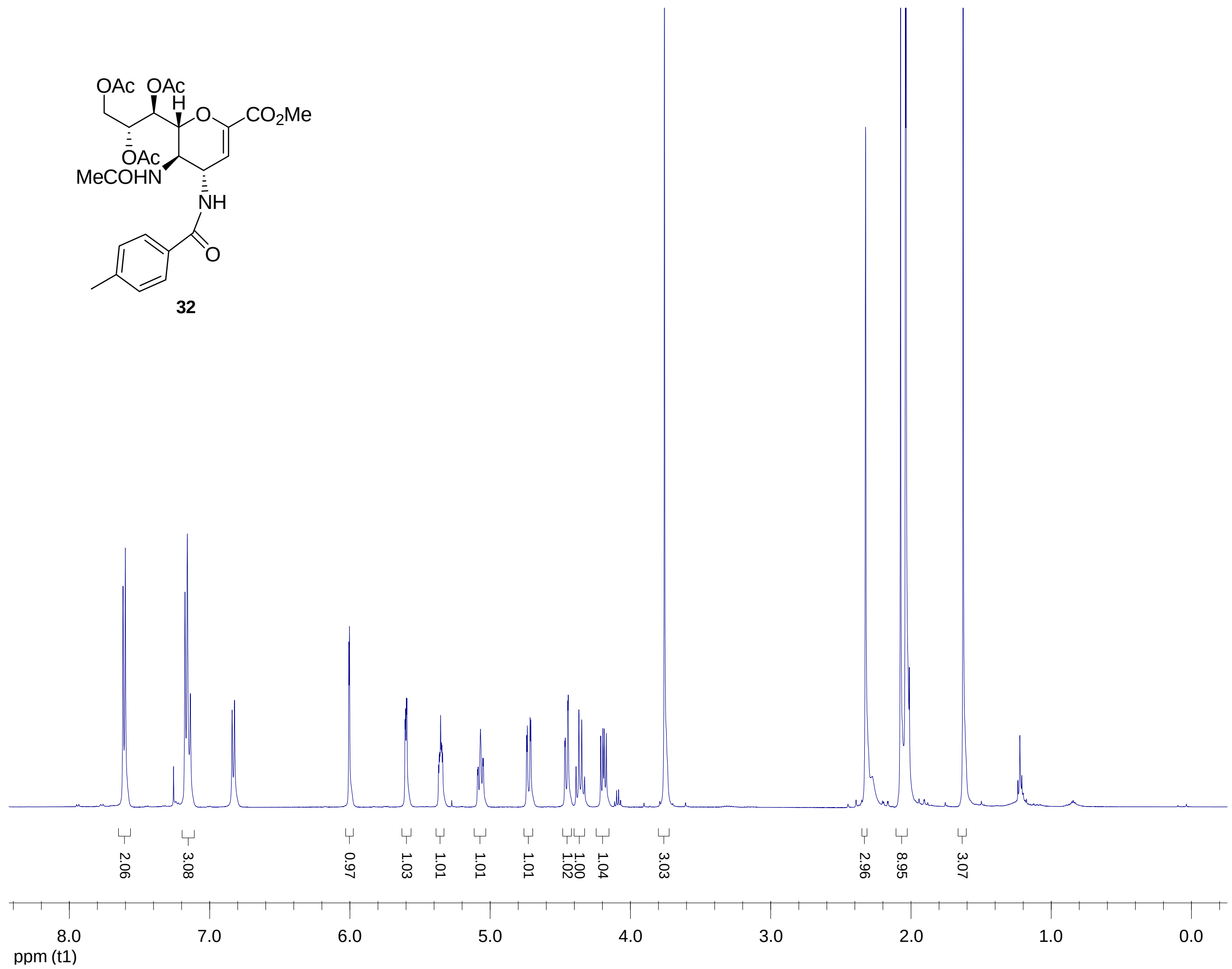
OAc OAc
H
O
CO2Me
OAc
MeCOHN
NH
O
32
2.06
3.08
0.97
1.03
1.01
1.01
1.01
1.02
1.00
1.04
3.03
2.96
8.95
3.07
8.0
7.0
6.0
5.0
4.0
3.0
2.0
1.0
0.0
ppm (t1)

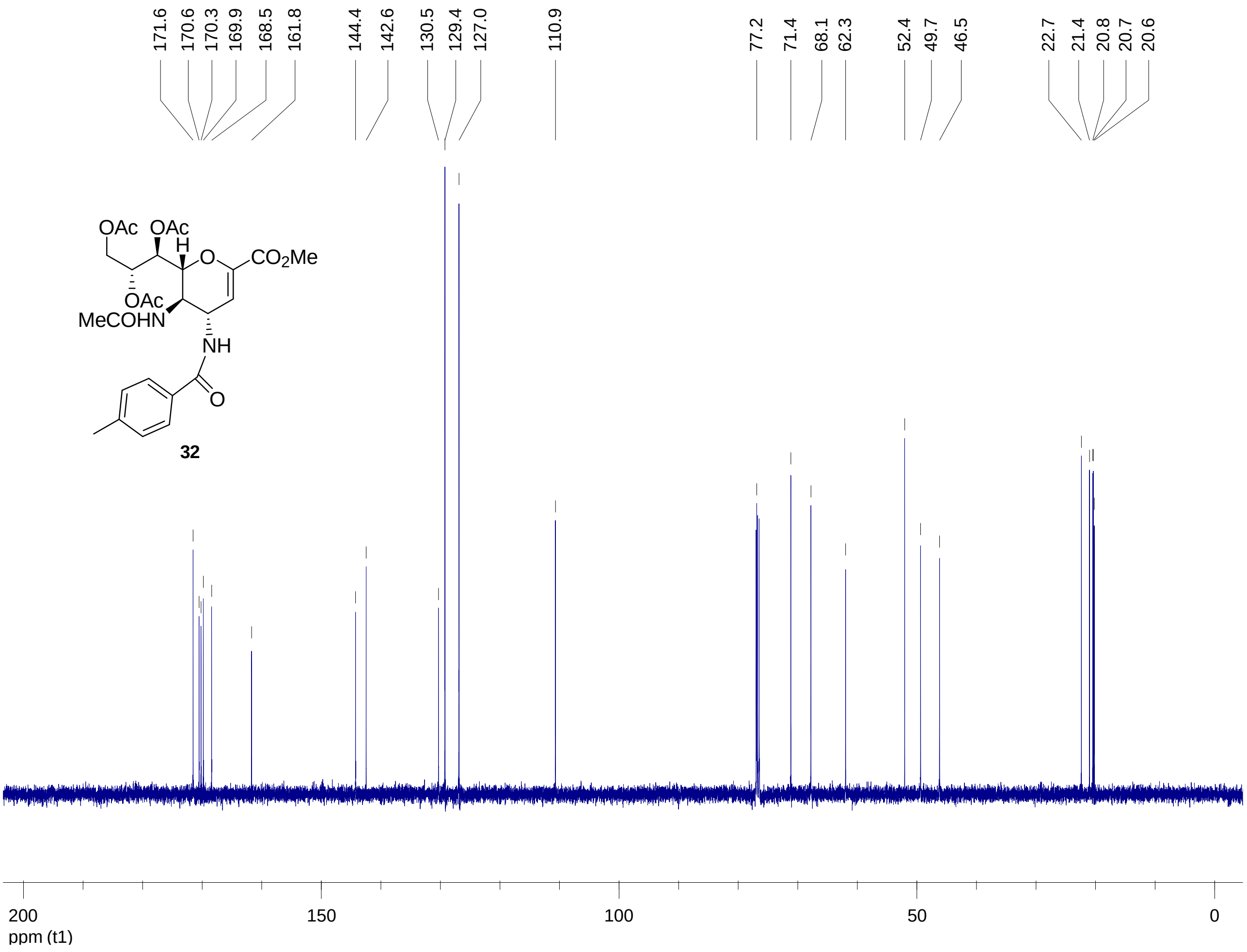
171.6
170.6
170.3
169.9
168.5
161.8
144.4
142.6
130.5
129.4
127.0
110.9
77.2
71.4
68.1
62.3
52.4
49.7
46.5
22.7
21.4
20.8
20.7
20.6
OAc OAc
H
O
CO2Me
OAc
MeCOHN
NH
O
32
200
150
100
50
0
ppm (t1)

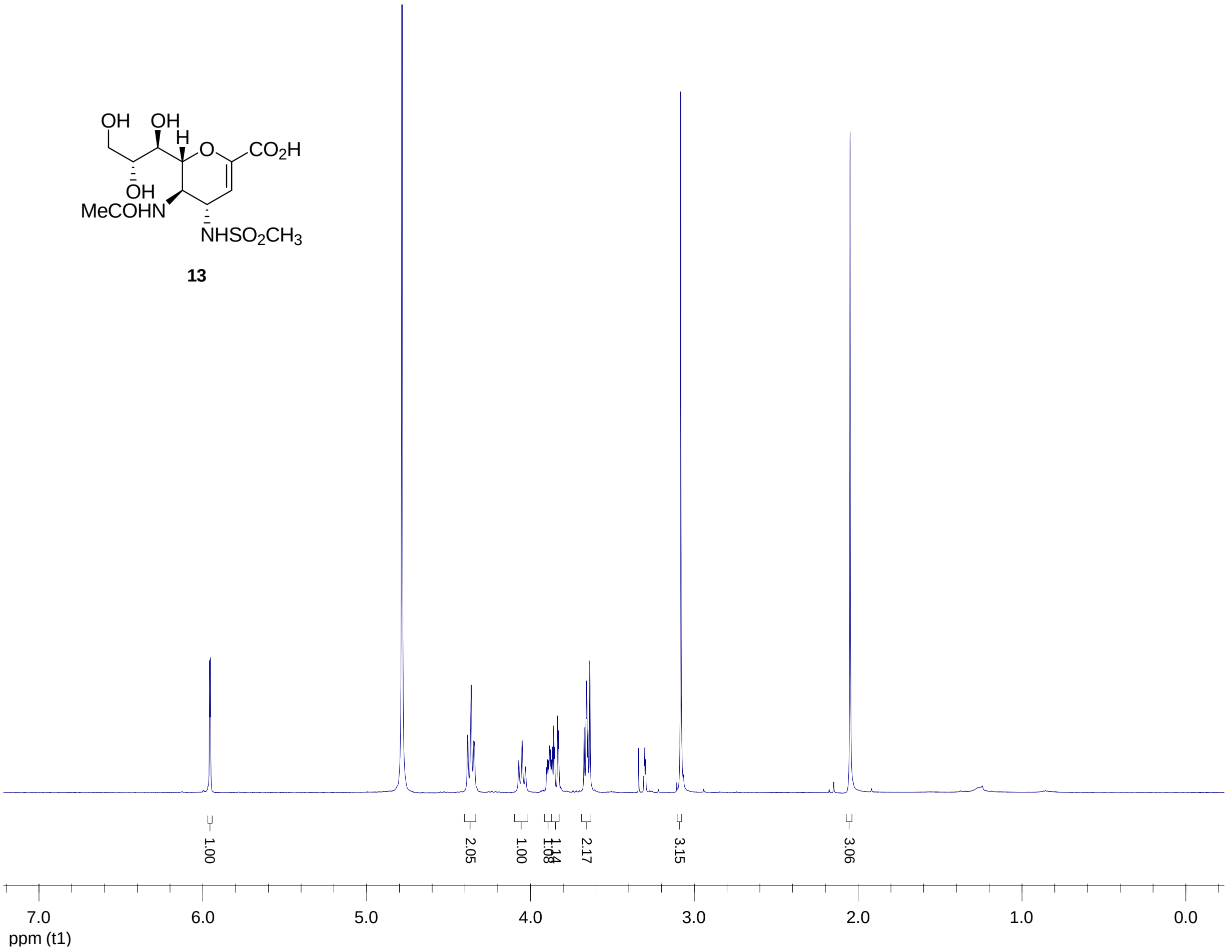
OH OH H O CO2H
OH
MeCOHN
NHSO2CH3
13
1.00
2.05
1.00
1.08
1.14
2.17
3.15
3.06
7.0
6.0
5.0
4.0
3.0
2.0
1.0
0.0
ppm (t1)

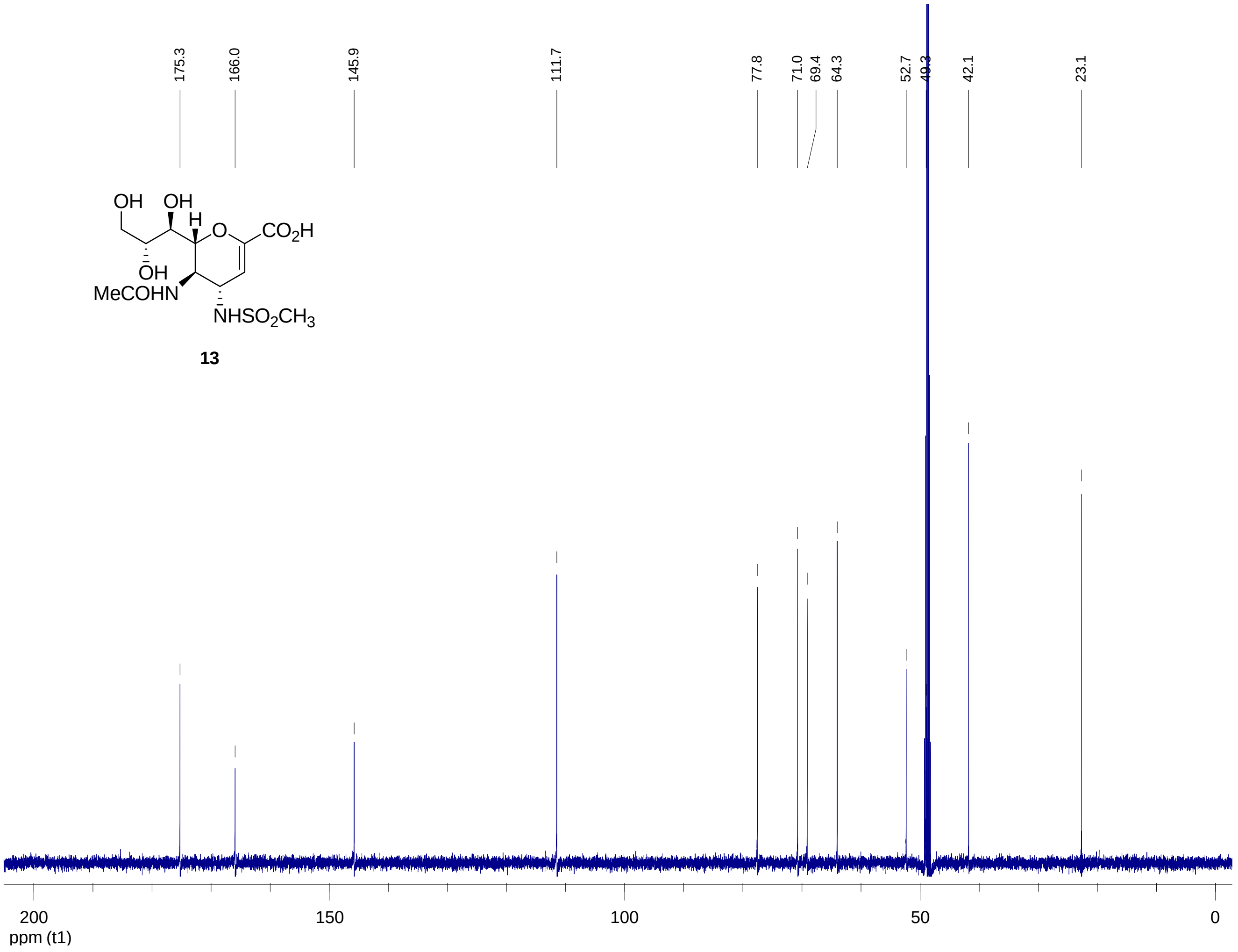
175.3
166.0
145.9
111.7
77.8
71.0
69.4
64.3
52.7
49.3
42.1
23.1
OH OH H O CO2H
OH
MeCOHN
NHSO2CH3
13
200
150
100
50
0
ppm (t1)

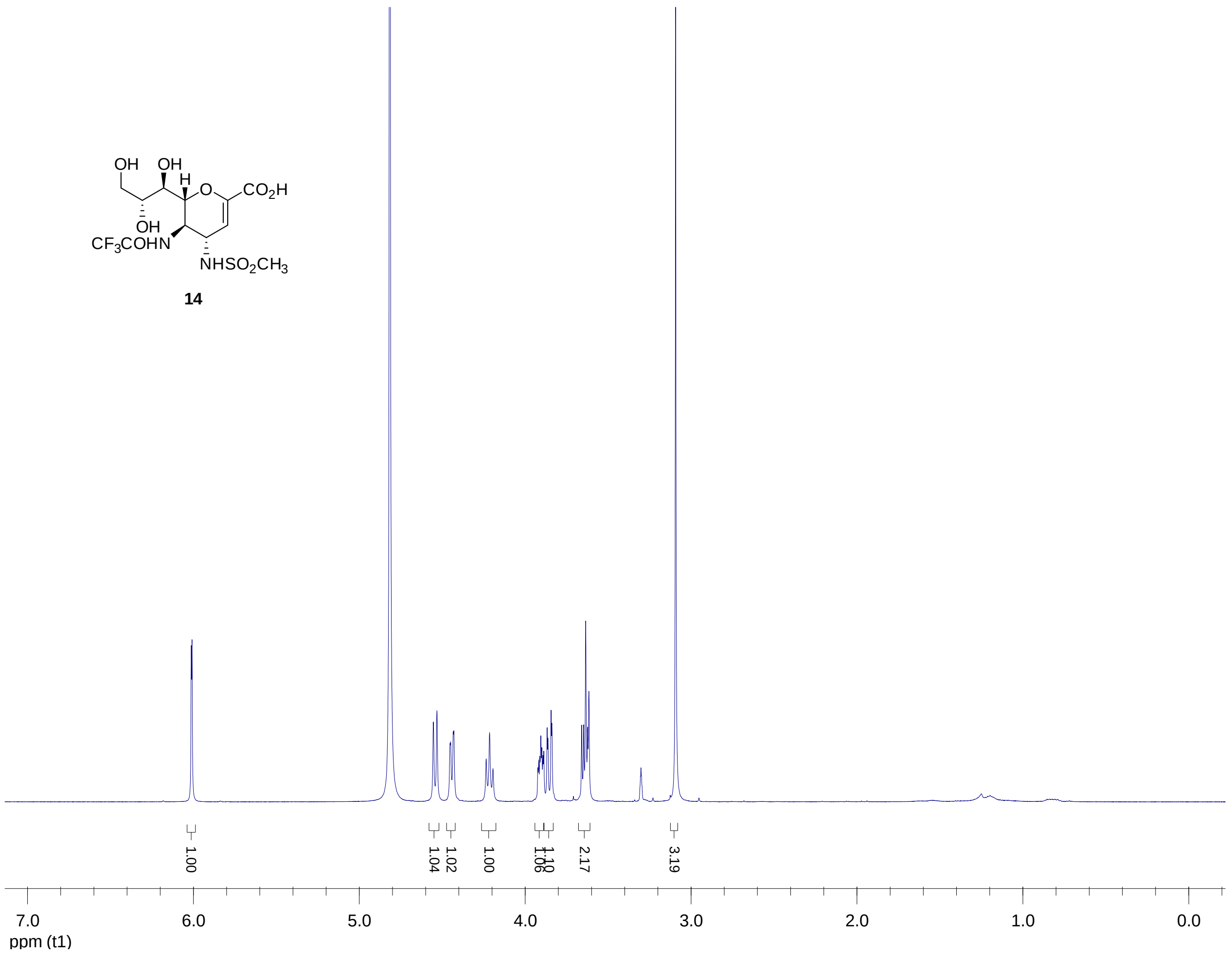

OH OH H O CO2H
OH
CF3COHN
NHSO2CH3
14
1.00
1.04
1.02
1.00
1.06
1.10
2.17
3.19
7.0
6.0
5.0
4.0
3.0
2.0
1.0
0.0
ppm (t1)

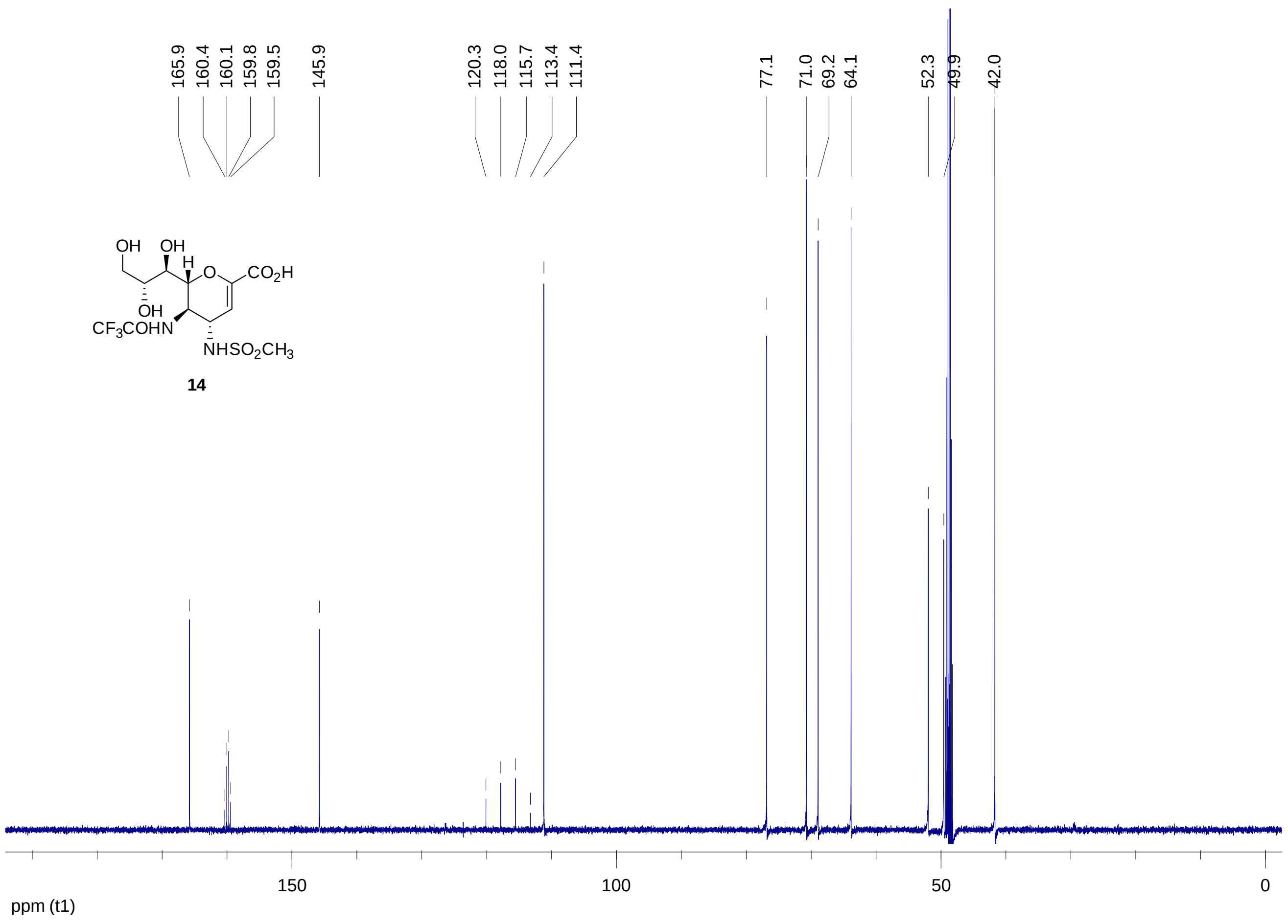

165.9
160.4
160.1
159.8
159.5
145.9
120.3
118.0
115.7
113.4
111.4
77.1
71.0
69.2
64.1
52.3
49.9
42.0
OH OH H O CO2H
OH
CF3COHN
NHSO2CH3
14
150
100
50
0
ppm (t1)

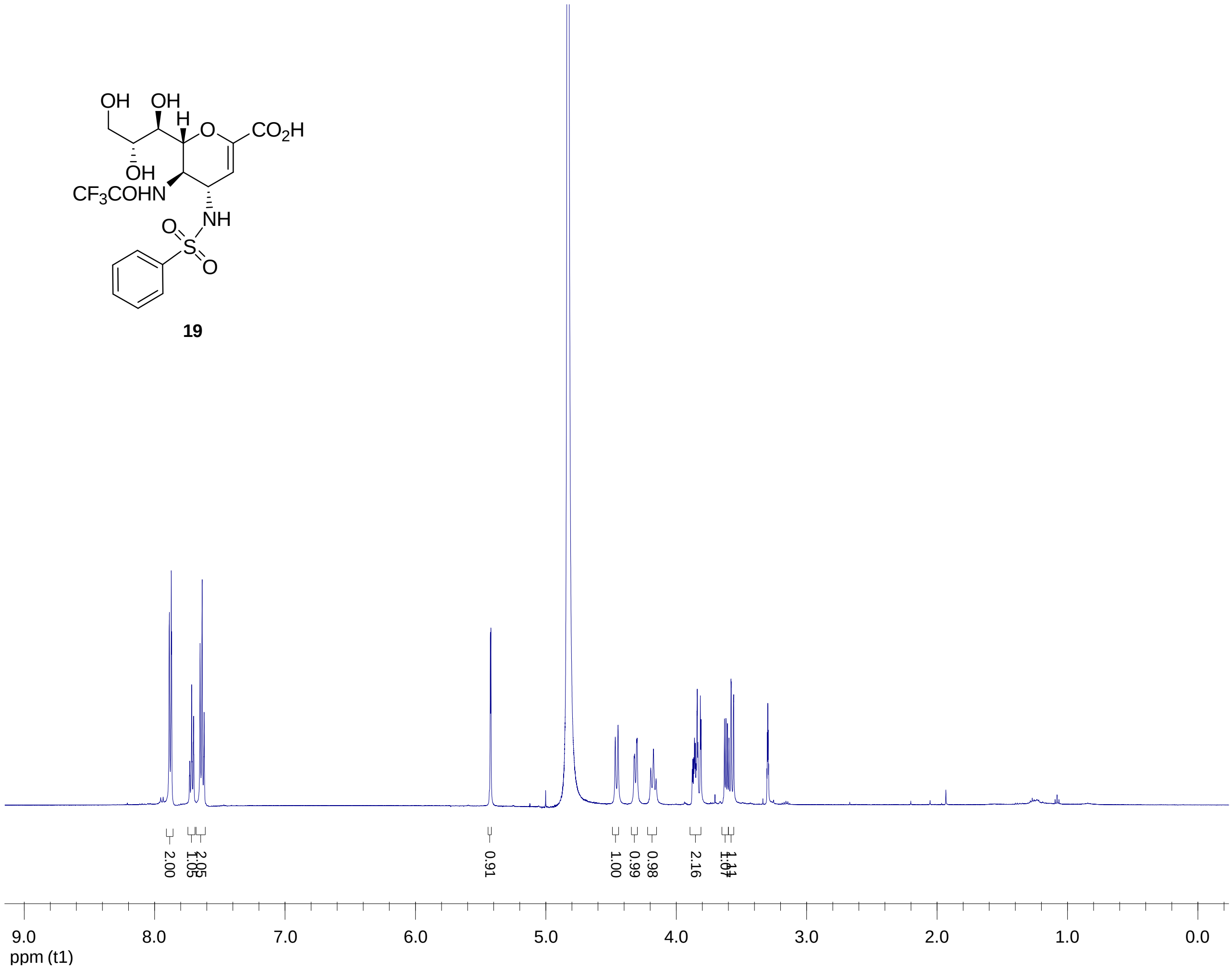
OH OH H O CO_2H
OH
CF_3COHN
NH
O S O
19
2.00
1.05
2.05
0.91
1.00
0.99
0.98
2.16
1.07
1.11
9.0
8.0
7.0
6.0
5.0
4.0
3.0
2.0
1.0
0.0
ppm (t1)

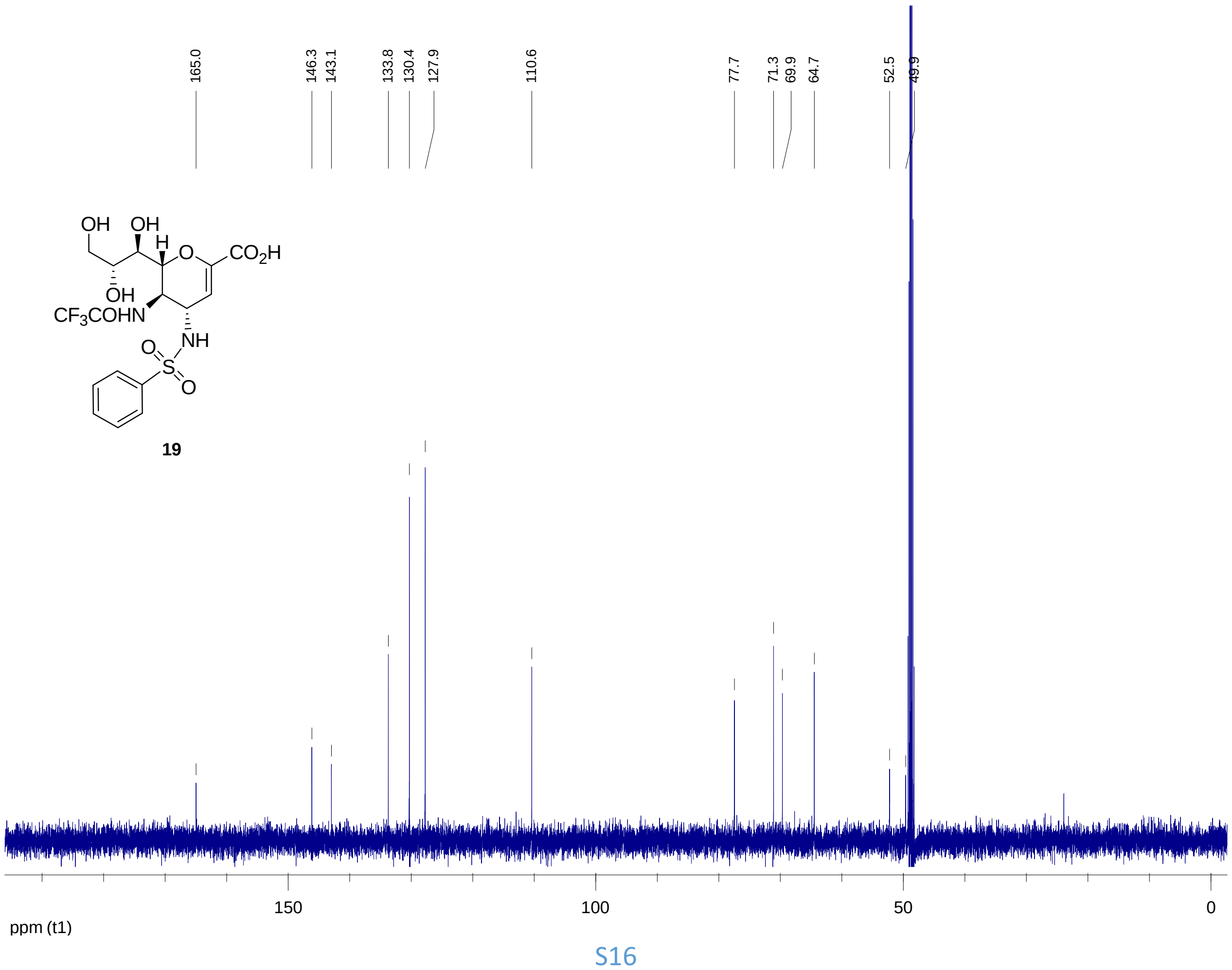
165.0
146.3
143.1
133.8
130.4
127.9
110.6
77.7
71.3
69.9
64.7
52.5
49.9
OH OH H O CO_2H
OH
CF_3COHN
NH
O S O
19
150
100
50
0
ppm (t1)

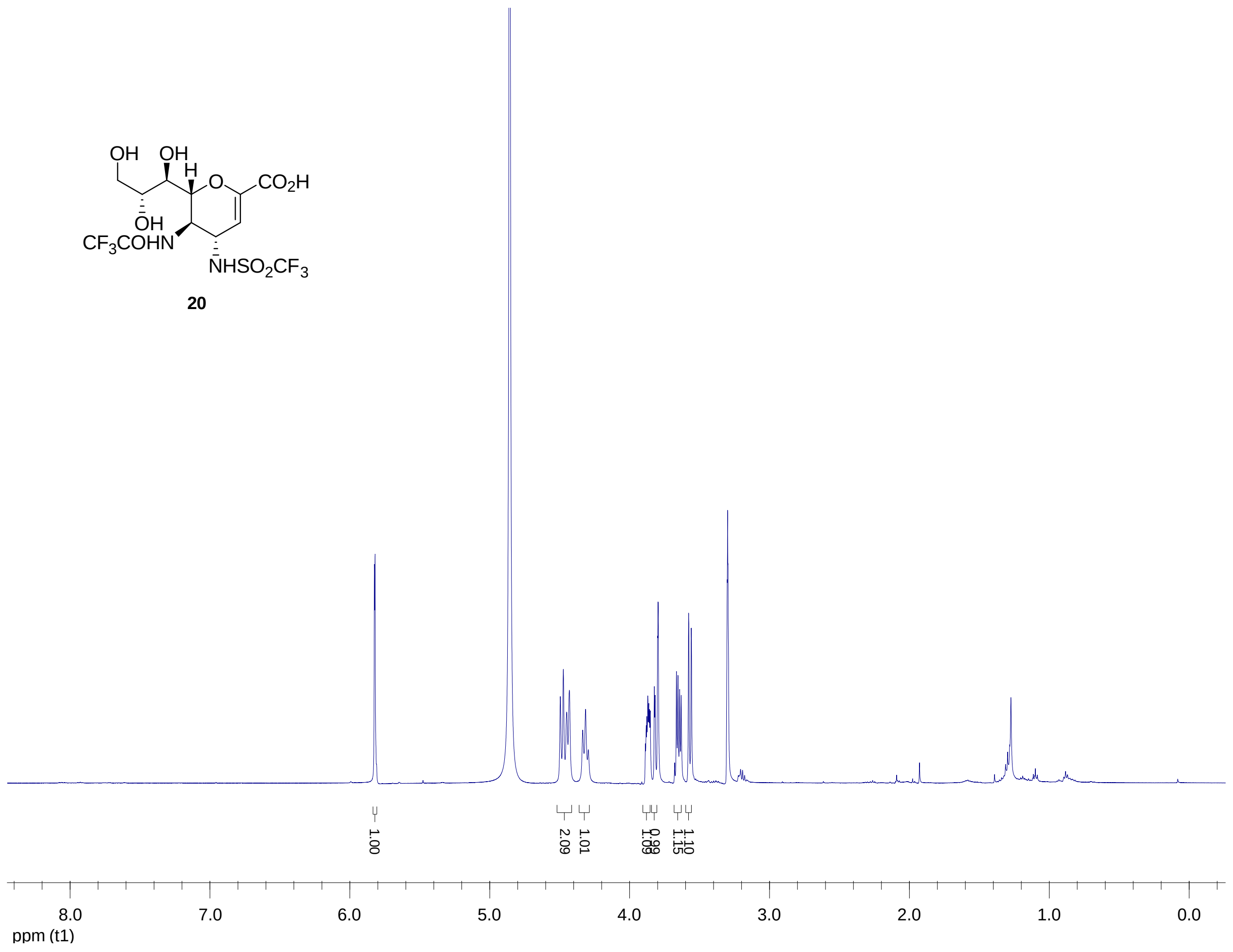

OH
OH
H
O
CO2H
OH
CF3COHN
NHSO2CF3
20
1.00
2.09
1.01
1.09
0.99
1.15
1.10
8.0
7.0
6.0
5.0
4.0
3.0
2.0
1.0
0.0
ppm (t1)

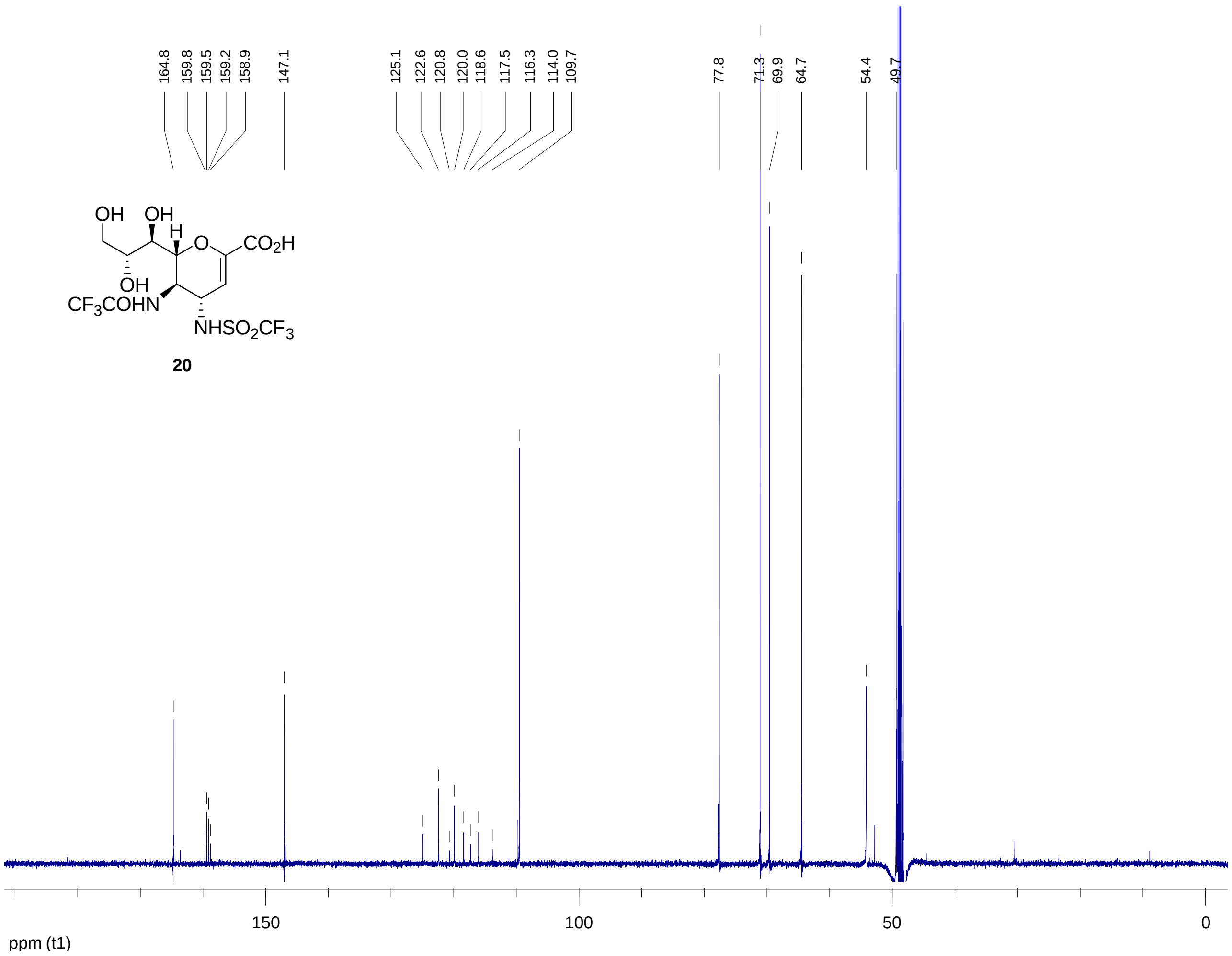

164.8
159.8
159.5
159.2
158.9
147.1
125.1
122.6
120.8
120.0
118.6
117.5
116.3
114.0
109.7
77.8
71.3
69.9
64.7
54.4
49.7
OH
OH
H
O
CO2H
OH
CF3COHN
NHSO2CF3
20
150
100
50
0
ppm (t1)

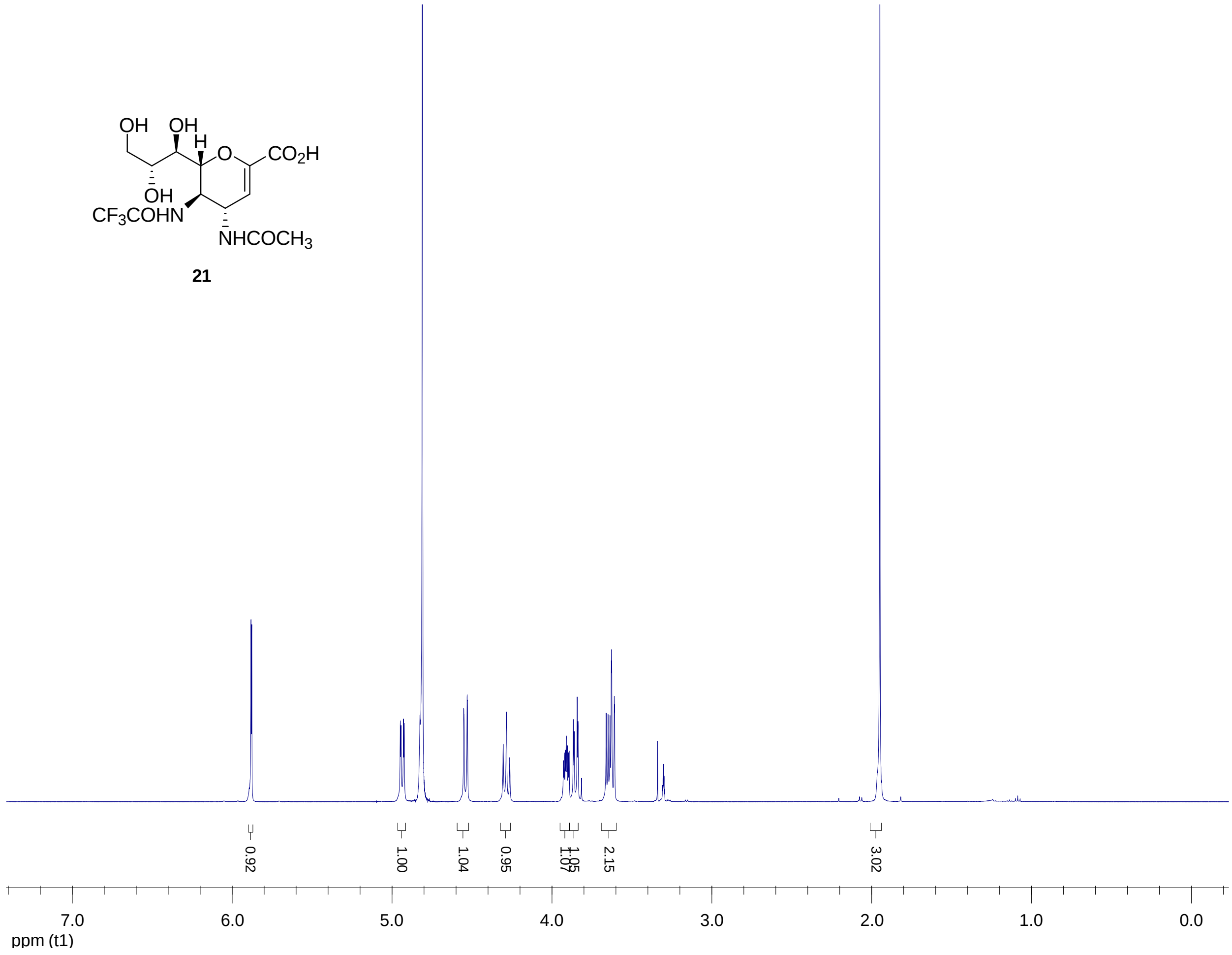
OH OH H O CO2H
OH
CF3COHN
NHCOCH3
21
0.92
1.00
1.04
0.95
1.07
1.05
2.15
3.02
7.0
6.0
5.0
4.0
3.0
2.0
1.0
0.0
ppm (t1)

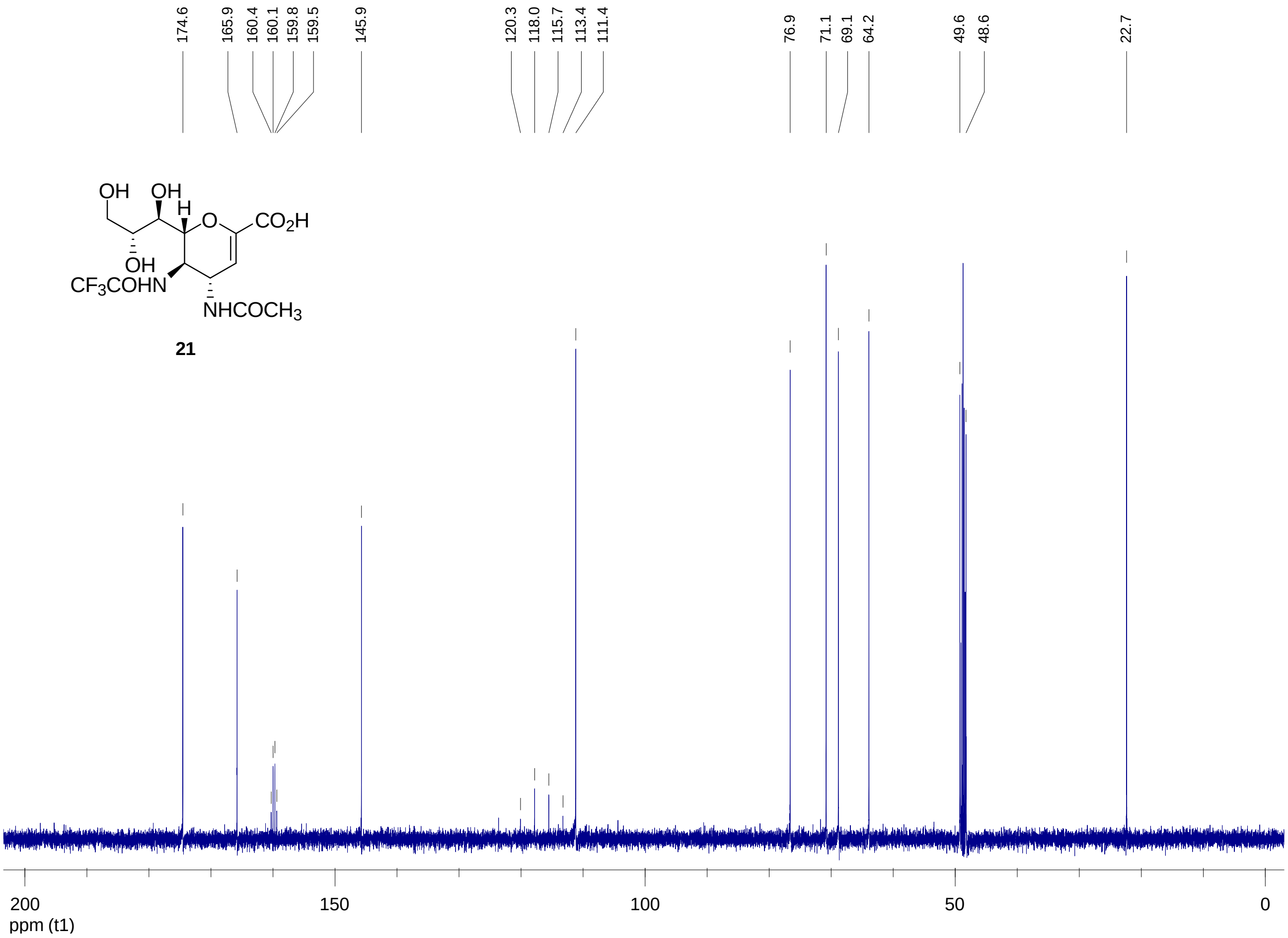
174.6
165.9
160.4
160.1
159.8
159.5
145.9
120.3
118.0
115.7
113.4
111.4
76.9
71.1
69.1
64.2
49.6
48.6
22.7
OH OH H O CO2H
OH
CF3COHN
NHCOCH3
21
200
150
100
50
0
ppm (t1)

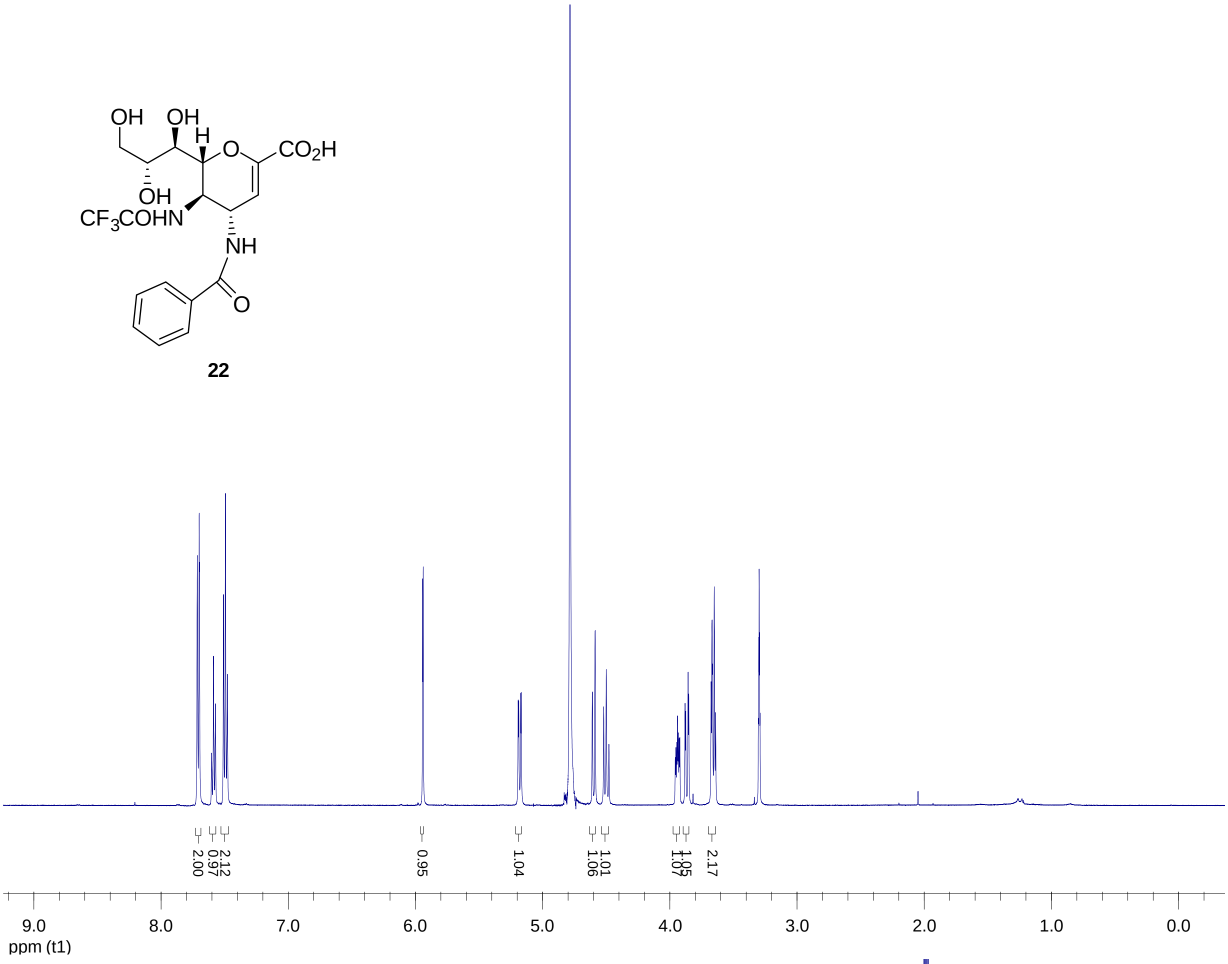
OH
OH
H
O
CO2H
OH
CF3COHN
NH
O
22
2.00
0.97
2.12
0.95
1.04
1.06
1.01
1.07
1.05
2.17
9.0
8.0
7.0
6.0
5.0
4.0
3.0
2.0
1.0
0.0
ppm (t1)

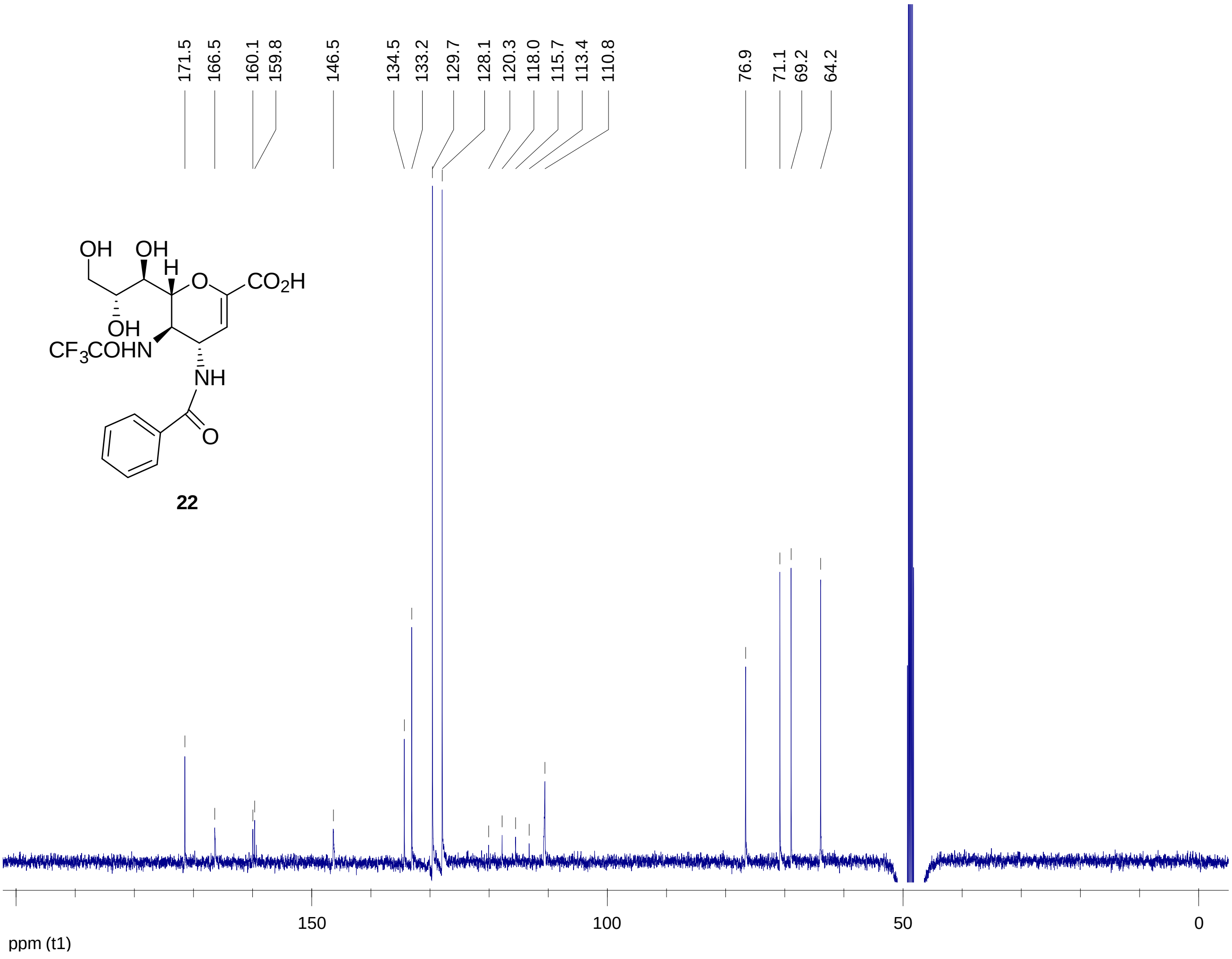
171.5
166.5
160.1
159.8
146.5
134.5
133.2
129.7
128.1
120.3
118.0
115.7
113.4
110.8
76.9
71.1
69.2
64.2
OH
OH
H
O
CO2H
OH
CF3COHN
NH
O
22
150
100
50
0
ppm (t1)

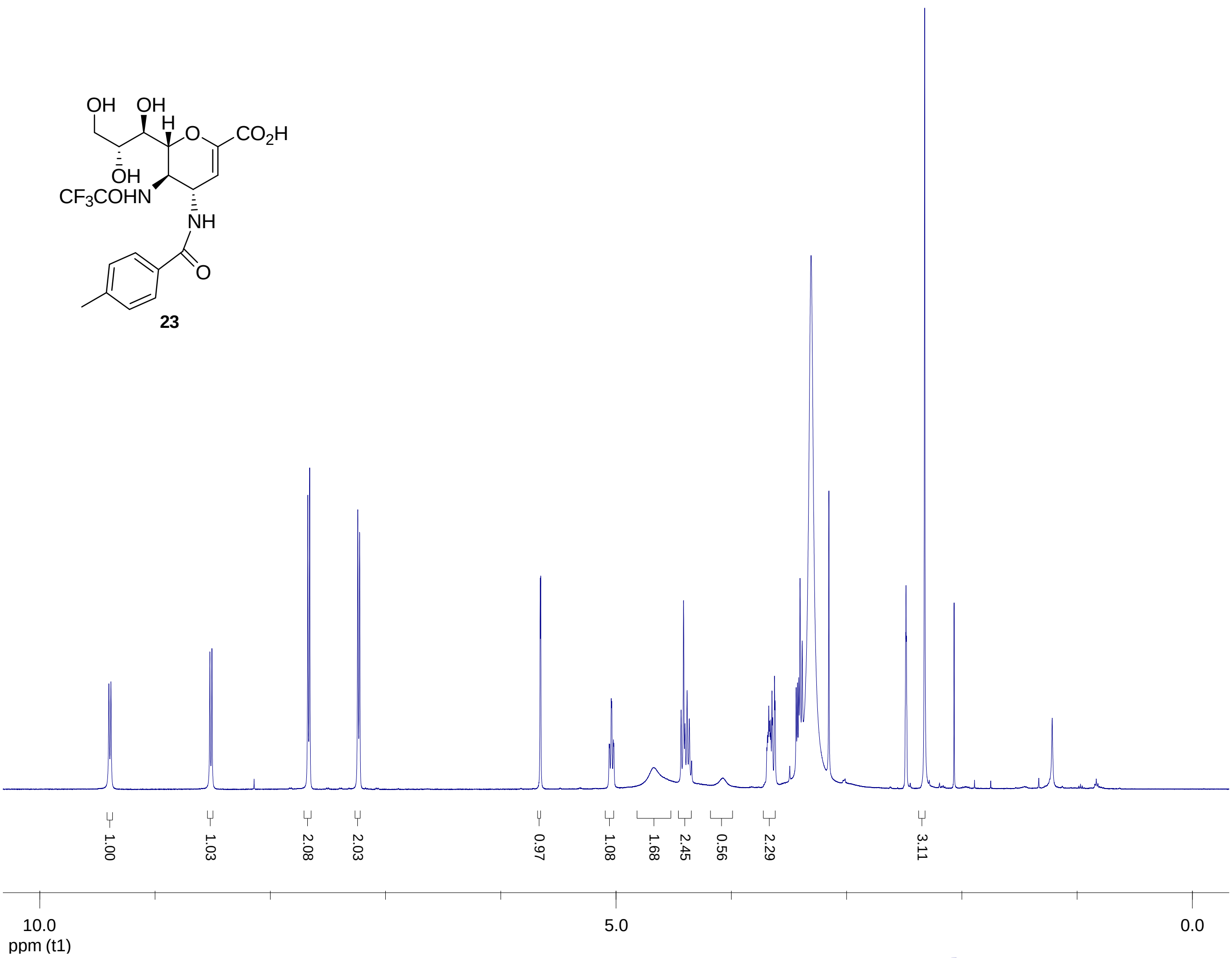
OH OH
H
O
CO2H
OH
CF3COHN
NH
O
23
1.00
1.03
2.08
2.03
0.97
1.08
1.68
2.45
0.56
2.29
3.11
10.0
5.0
0.0
ppm (t1)

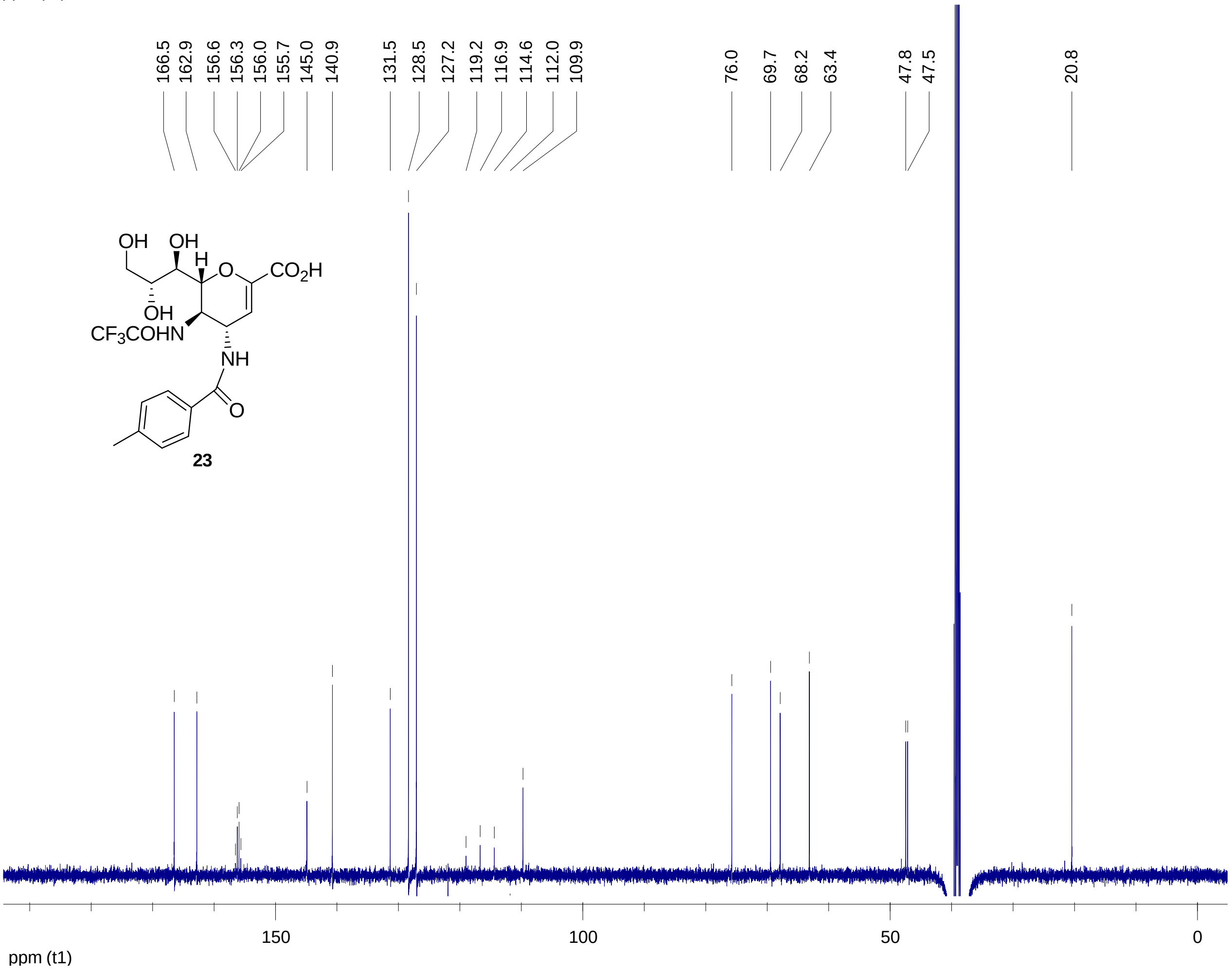
166.5
162.9
156.6
156.3
156.0
155.7
145.0
140.9
131.5
128.5
127.2
119.2
116.9
114.6
112.0
109.9
76.0
69.7
68.2
63.4
47.8
47.5
20.8
OH OH
H
O
CO2H
OH
CF3COHN
NH
O
23
150
100
50
0
ppm (t1)

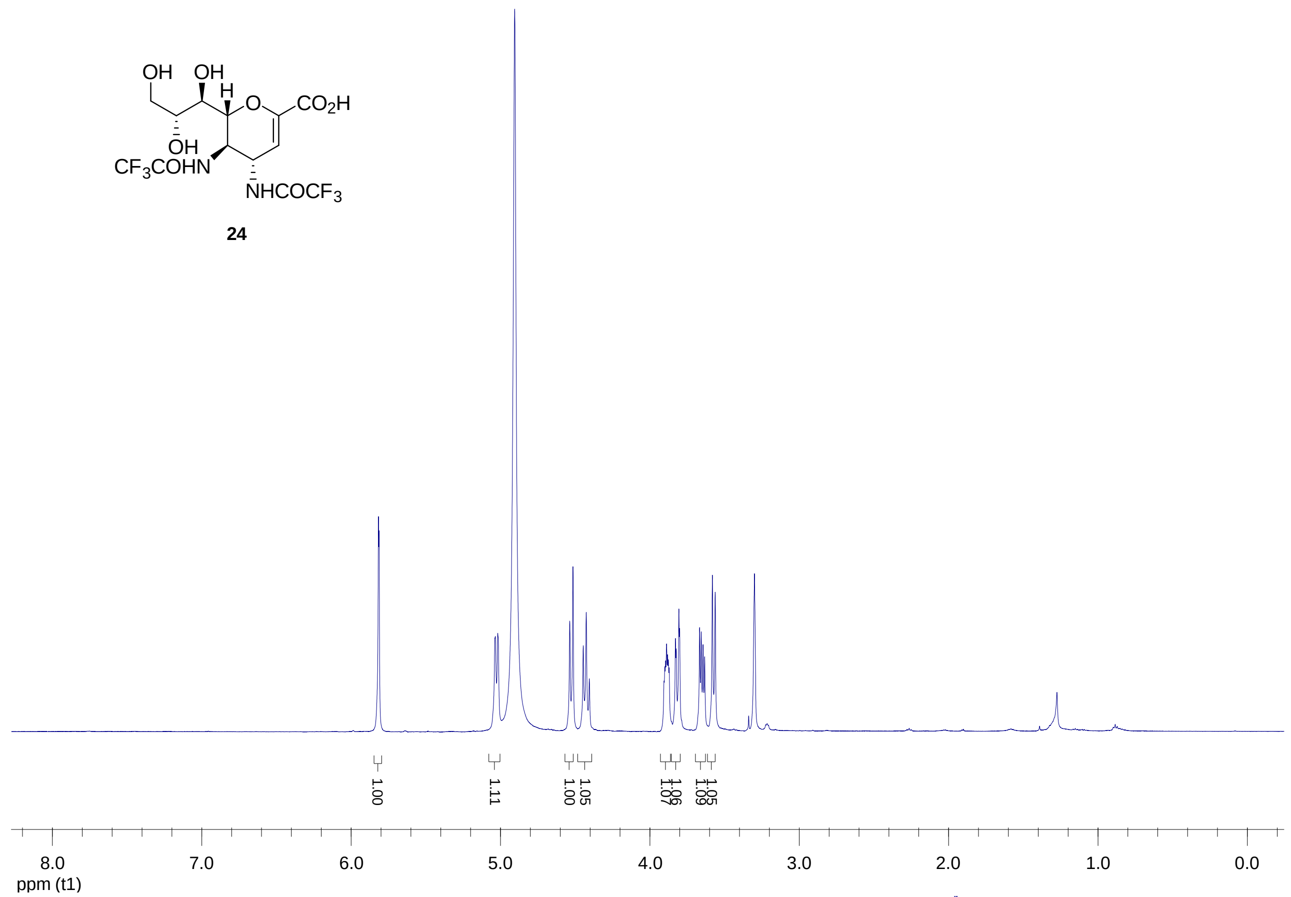
OH OH H O CO2H
OH
CF3COHN
NHCOCF3
24
1.00
1.11
1.00
1.05
1.07
1.06
1.09
1.05
8.0 7.0 6.0 5.0 4.0 3.0 2.0 1.0 0.0
ppm (t1)

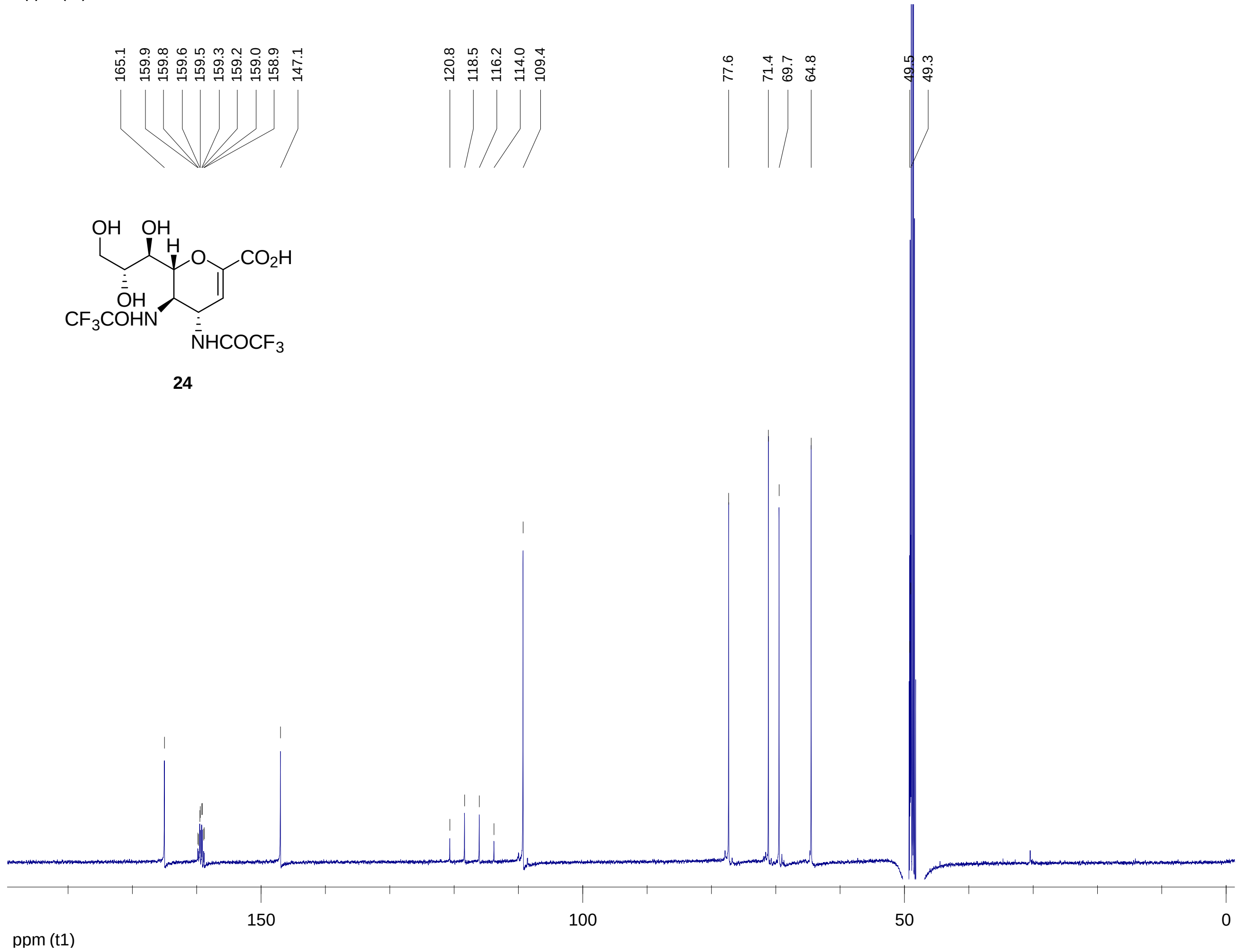
165.1 159.9 159.8 159.6 159.5 159.3 159.2 159.0 158.9 147.1
120.8 118.5 116.2 114.0 109.4
77.6 71.4 69.7 64.8
49.5 49.3
OH OH H O CO2H
OH
CF3COHN
NHCOCF3
24
150 100 50 0
ppm (t1)

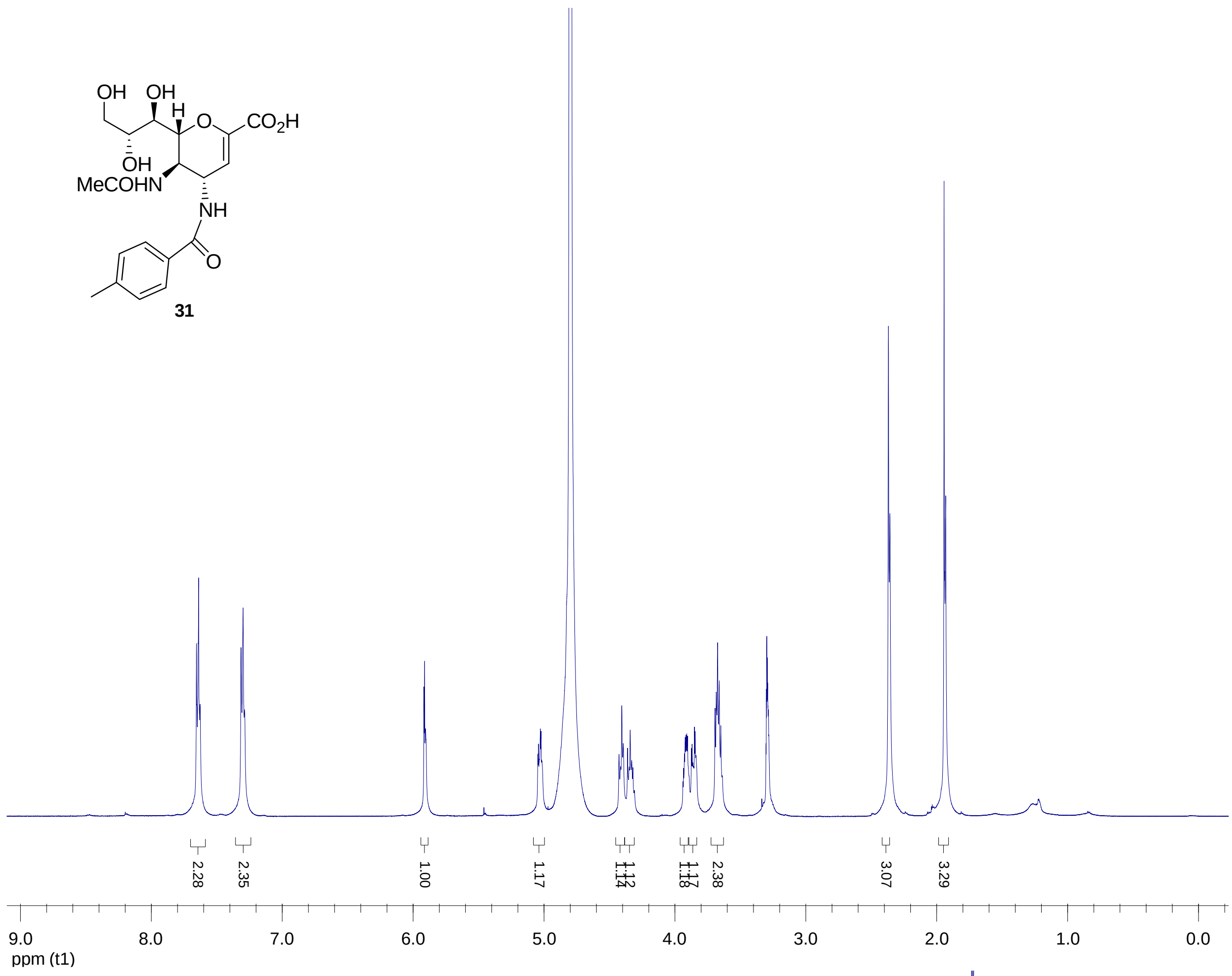
OH
OH
H
O
CO2H
OH
MeCOHN
NH
O
31
2.28
2.35
1.00
1.17
1.12
1.18
2.38
3.07
3.29
9.0
8.0
7.0
6.0
5.0
4.0
3.0
2.0
1.0
0.0
ppm (t1)

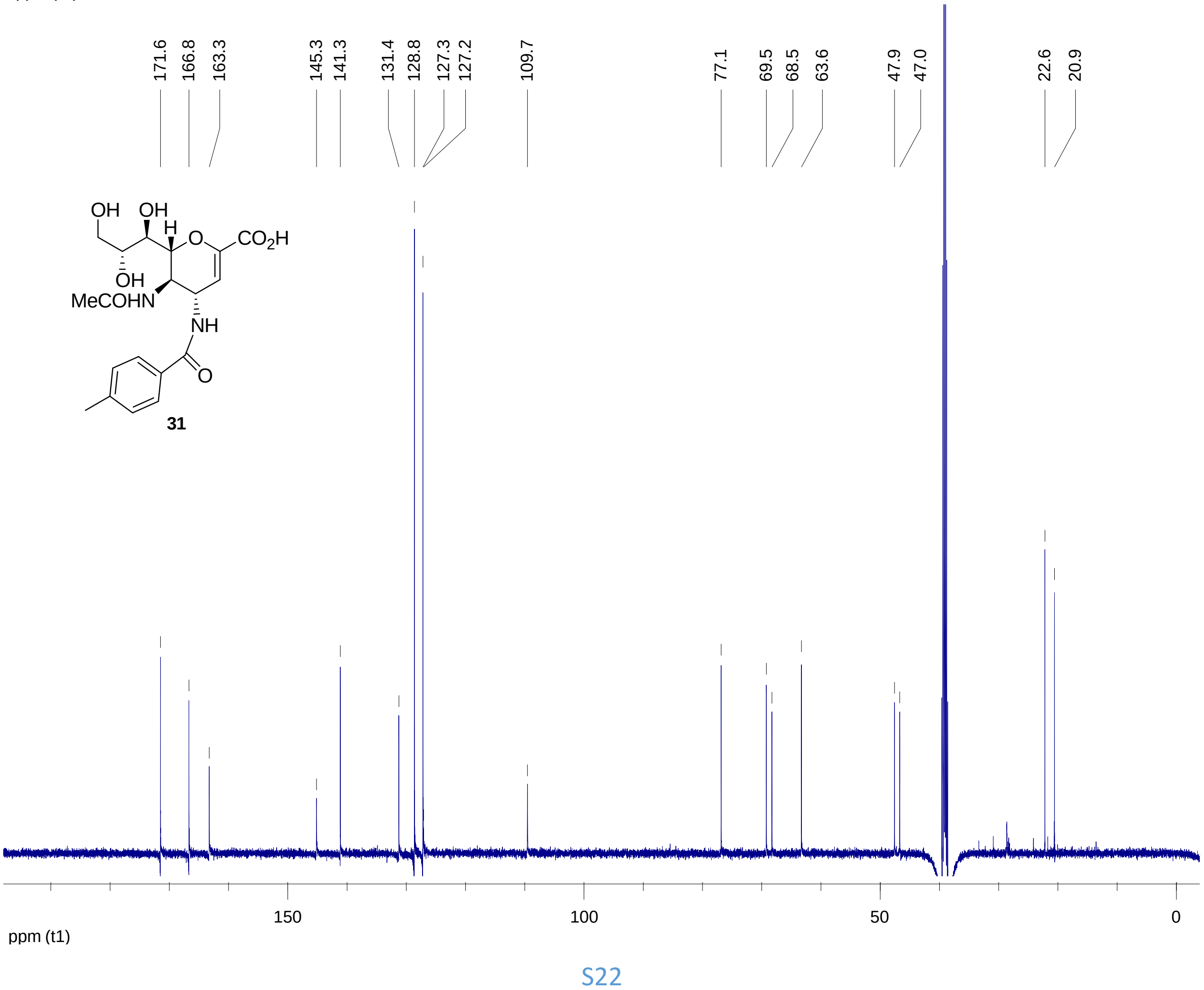
171.6
166.8
163.3
145.3
141.3
131.4
128.8
127.3
127.2
109.7
77.1
69.5
68.5
63.6
47.9
47.0
22.6
20.9
OH
OH
H
O
CO2H
OH
MeCOHN
NH
O
31
150
100
50
0
ppm (t1)
